# Supplementary material for: Overcoming thermostability challenges in mRNA–lipid nanoparticle systems with piperidine-based ionizable lipids
Source: Commun Biol. 2024 May 10;7:556. doi: 10.1038/s42003-024-06235-0 (PMC11087515; doi:10.1038/s42003-024-06235-0)
Supplement: Supplementary file 2 — Supporting Information [file 42003_2024_6235_MOESM2_ESM.docx]

Supporting Information for

**Overcoming Thermostability Challenges in mRNA–Lipid Nanoparticle Systems with Piperidine-Based Ionizable Lipids**

*Kazuki Hashiba^1,*^, Masamitsu Taguchi^1^, Sachiko Sakamoto^1^, Ayaka Otsu^1^, Yoshiki Maeda^1^, Hirofumi Ebe^1^, Arimichi Okazaki^1^, Hideyoshi Harashima^2^, Yusuke Sato^2,*^*

*^1^Nucleic Acid Medicine Business Division, Nitto Denko Corporation, 1-1-2, Shimohozumi, Ibaraki, Osaka, 567-8680, Japan*

*^2^Laboratory for Molecular Design of Pharmaceutics, Faculty of Pharmaceutical Sciences, Hokkaido University, Kita-12, Nishi-6, Kita-Ku, Sapporo 060-0812, Japan*

**Corresponding authors:*

*K. Hashiba*

*E-mail:* [*kazuki.hashiba@nitto.com*](mailto:kazuki.hashiba@nitto.com)

*Y. Sato*

*E-mail:* [*y_sato@pharm.hokudai.ac.jp*](mailto:y_sato@pharm.hokudai.ac.jp)

Contents

[**Supporting figures** 3](#_Toc159513958)

[**Supporting tables** 22](#_Toc159513959)

[**Lipid synthesis** 26](#_Toc159513960)

[**References** 68](#_Toc159513961)

# **Supporting figures**


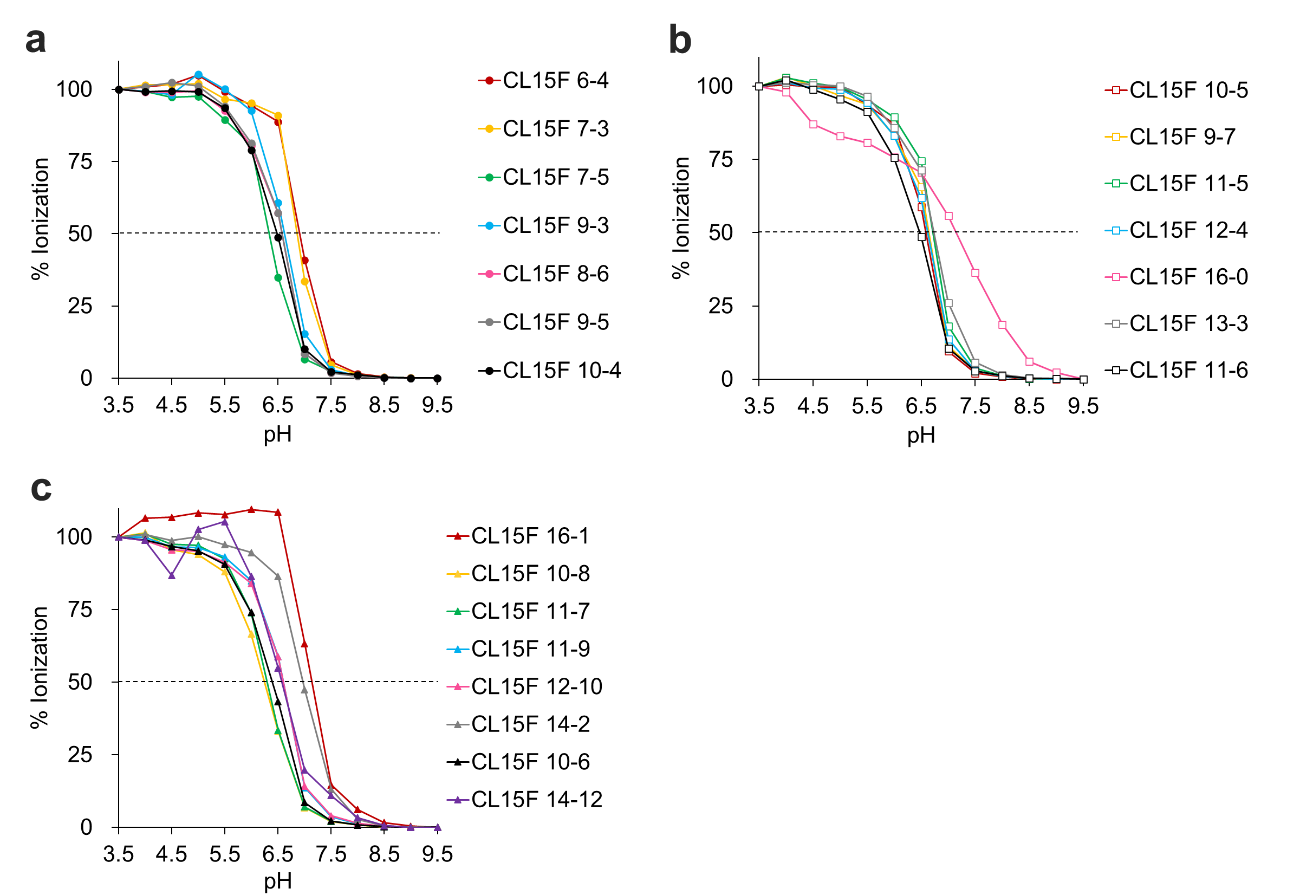


**Figure S1. Ionization ability of CL15-LNPs. Percentage of ionized lipid in different pH environments determined by TNS assay.**

a–c) For easier visibility, the 22 LNP ionization profiles have been divided into three graphs.


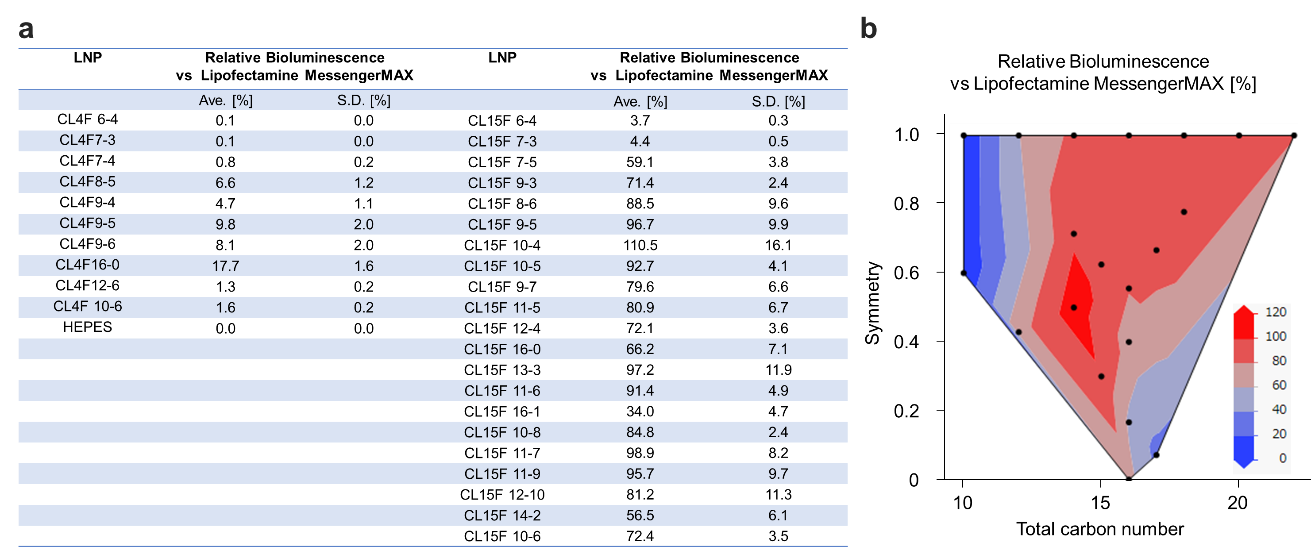


**Figure S2. Impact of CL15 scaffold structure on *in vitro* functional delivery of luciferase mRNA.**

a, b) *In vitro* functional delivery of luciferase mRNA summarized as a table (a) and contour plot (b). The level is expressed as relative activity (%) against MessengerMAX used as a positive control.

The lipid tail structure is described using two parameters (total carbon number and symmetry) based on main chain and side chain lengths of ionizable lipid tail. The total carbon number for the branched tail is calculated as (main chain length)  +  (side chain length). The symmetry of the main chain and side chain is calculated as the ratio of (side chain length) to (main chain length − 2). For example, CL15F 9–7, CL15F 12–4, and CL15F 16–0 have the same total carbon number (16) in each tail. In contrast, they are fully symmetrical (1.0), moderately symmetrical (0.4), and linear (0.0), respectively (y-axis).


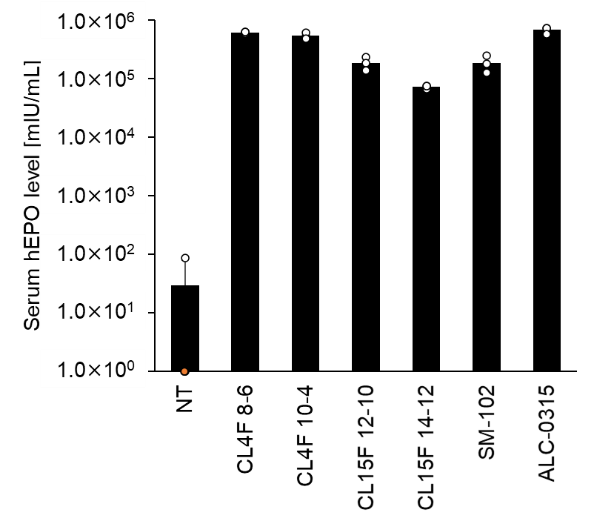


**Figure S3. Serum hEPO levels in mice treated with fresh mRNA/LNPs**

Six hours after intravenous administration of hEPO mRNA carrying LNPs, serum was collected and hEPO level was quantified using a Human EPO ELISA Kit (Thermo Fisher Scientific, MA, USA). Orange highlight indicates serum hEPO level lower than 1.0 mIU/mL. Mean ± SD.


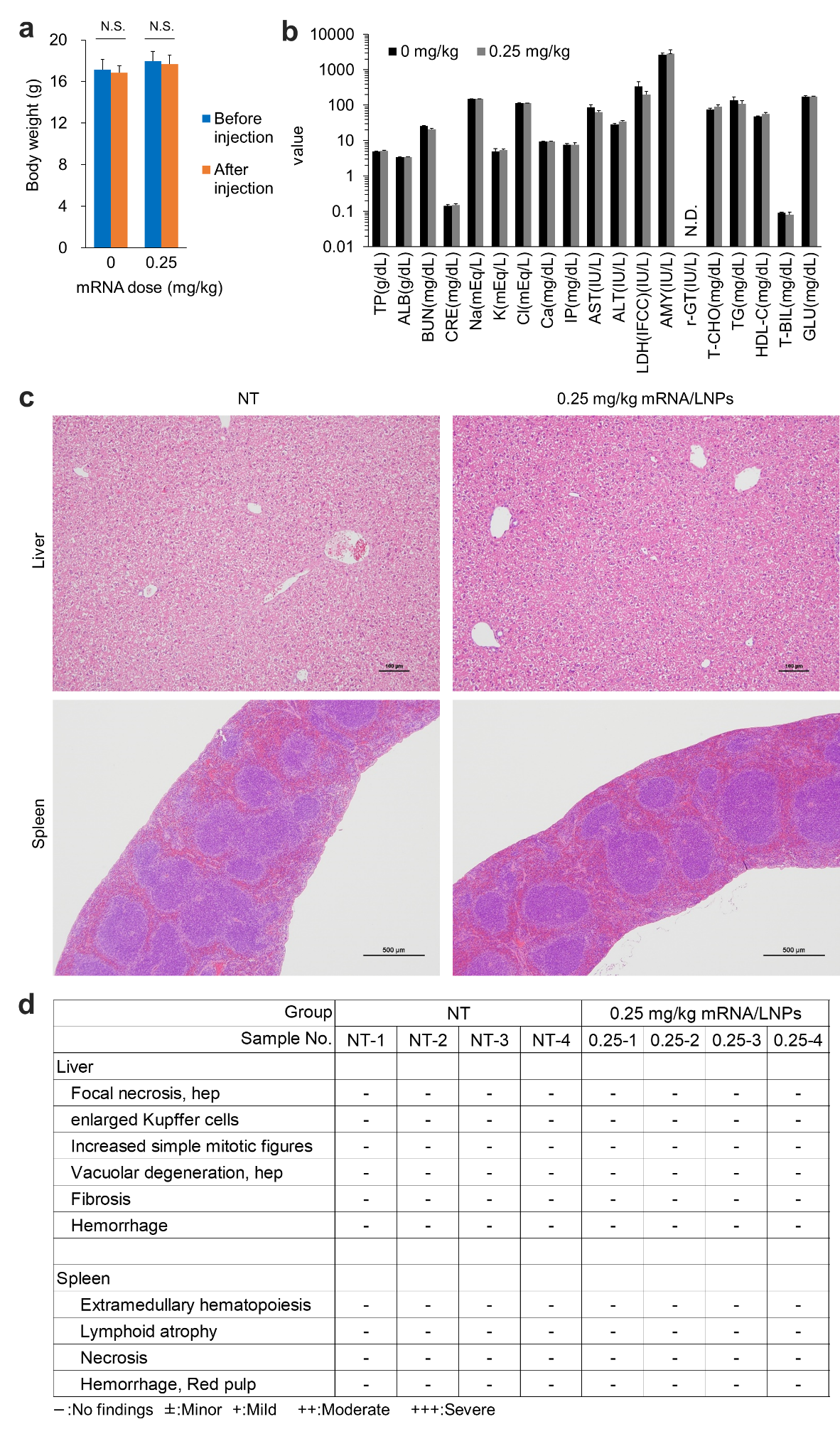


**Figure S4. Single dose toxicity studies *in vivo***

a–c) Body weight measurement (a), hematological test (b), hematoxylin-eosin staining of liver and spleen (c), and histopathological analysis (d) was performed 24 h after i.v. administration of CL15F 14-12 LNPs (0.25 mg/kg FLuc mRNA per mouse, n = 4 biologically independent balb/c mice per group). a) Body weight change was not significant based on paired Student’s t-test. b) Serum chemistry parameters were measured at Oriental yeast Co., Ltd (Shiga, Japan). Serum chemistry parameters were not significantly changed based on unpaired Student’s t-test. TP: total protein, ALB: albumin, BUN: blood urea nitrogen, CRE: creatinine, IP: inorganic phosphates, AST: aspartate transaminase, ALT: alanine transaminase, LDH: lactose dehydrogenase, AMY: amylase, γ-GT: gamma-glutamyl transpeptidase, T-CHO: total cholesterol, TG: triglyceride, HDL-C: high density lipoprotein cholesterol, T-BIL: total bilirubin, GLU: glucose. c) Liver and spleen were fixed in Mildform 10N and 3 µm slices were stained with hematoxylin-eosin. Scale bars represent 100 µm and 500 µm for liver and spleen, respectively. d) Histopathological analysis of liver and spleen was performed at the Sapporo General Pathology Laboratory Co., Ltd. (Hokkaido, Japan). Mean ± SD.


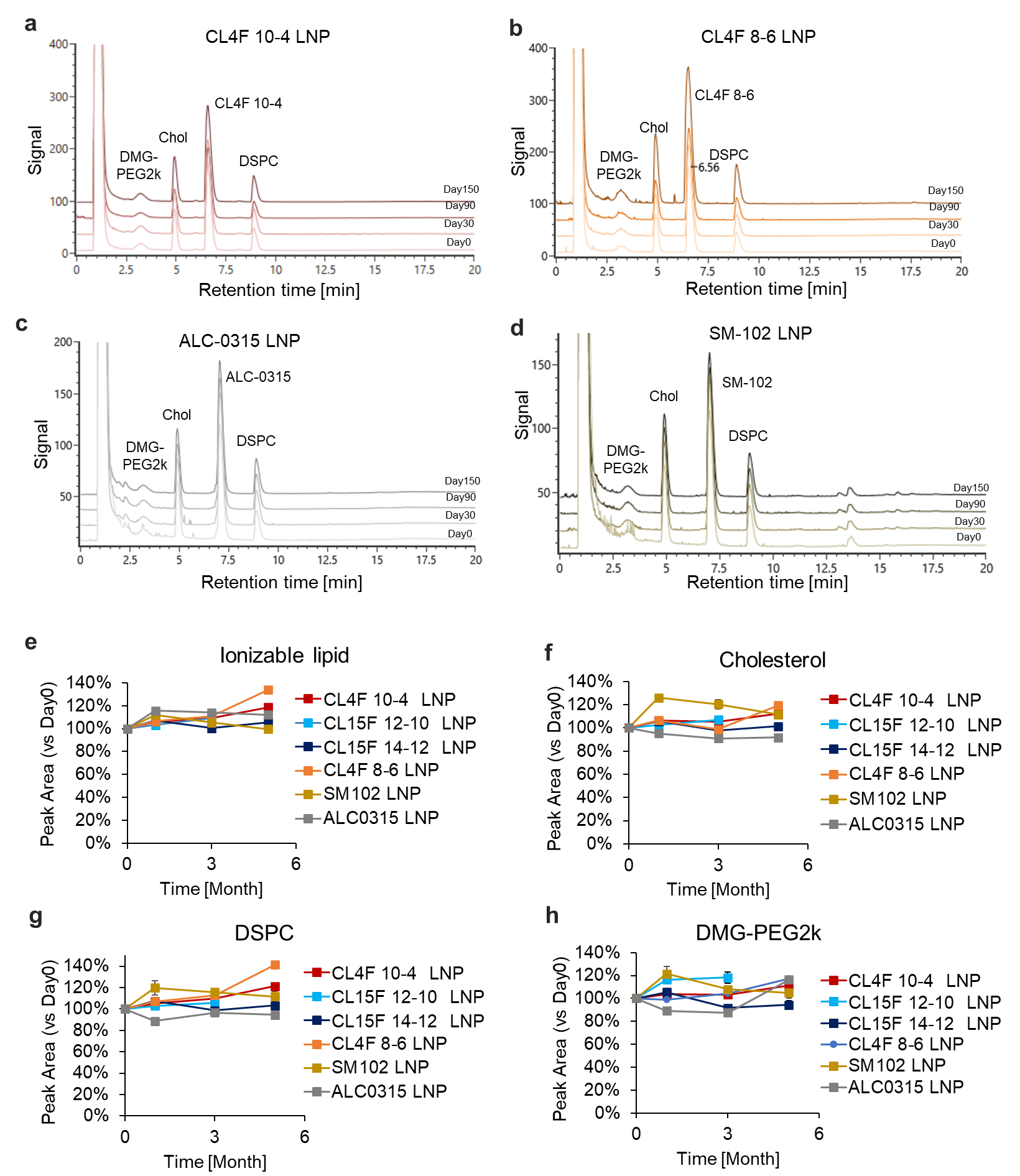


**Figure S5. Relative changes in lipid integrity over time**

a–d) hEPO mRNA/LNPs were diluted 100-fold with 70% isopropanol and lipid integrity was analyzed using a Waters BioAccord System with a charged aerosol detector (CAD). The separation was carried out using an ACQUITY UPLC BEH C18 Column, 130 Å, 1.7 µm, 2.1 mm, 100 mm and a gradient of 80–100% isopropanol/acetonitrile (62:33) in water with 5 mM ammonium acetate over 15 min and held at 100% isopropanol/acetonitrile (62:33) with 5 mM ammonium acetate for 5 min at 0.3 mL/min. Injection volume was 2.0 µL and the column temperature was 60 °C. e–h) Relative changes in each component were calculated from the peak areas based on the CAD spectra. To accurately quantify DMG-PEG2k, 10 µL of samples was injected. Mean ± SD.


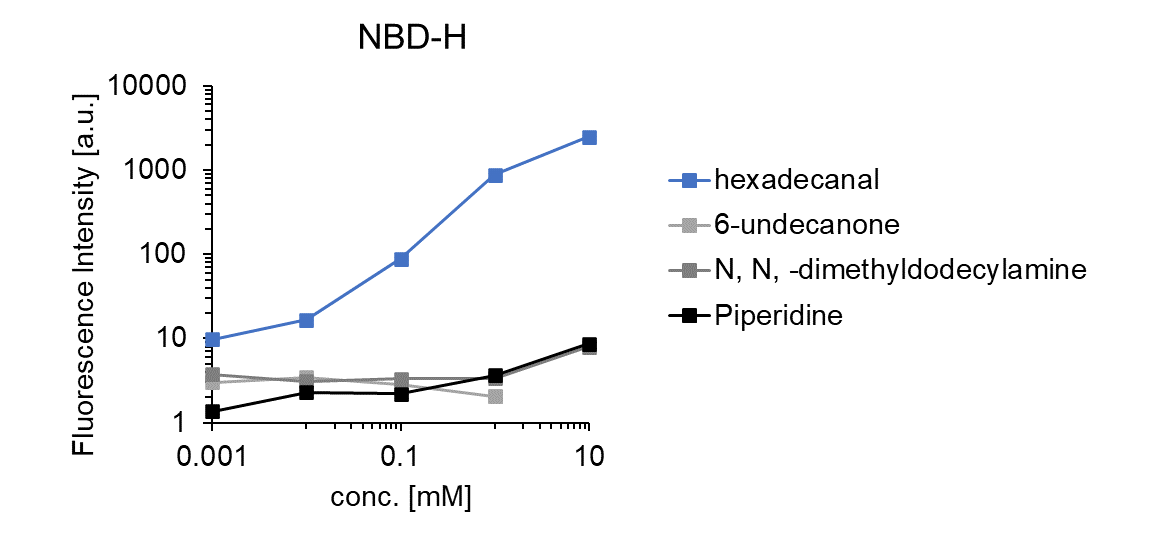


**Figure S6. NBD-H emits fluorescence in response to aldehydes**

NBD-H acetonitrile solution (250 µM) containing 0.025% trifluoroacetic acid (TFA) was prepared; 285 µL of NBD-H solution was added to 15 µL of 40 mM lipid in ethanol and incubated for 60 min at 25 °C. After incubation, fluorescence intensity was measured using a Varioskan LUX Multimode Microplate Reader (Thermo Fisher Scientific, USA) at an excitation wavelength of 470 nm and an emission wavelength of 550 nm in black 96-well plates at a total volume of 200 µL.

**
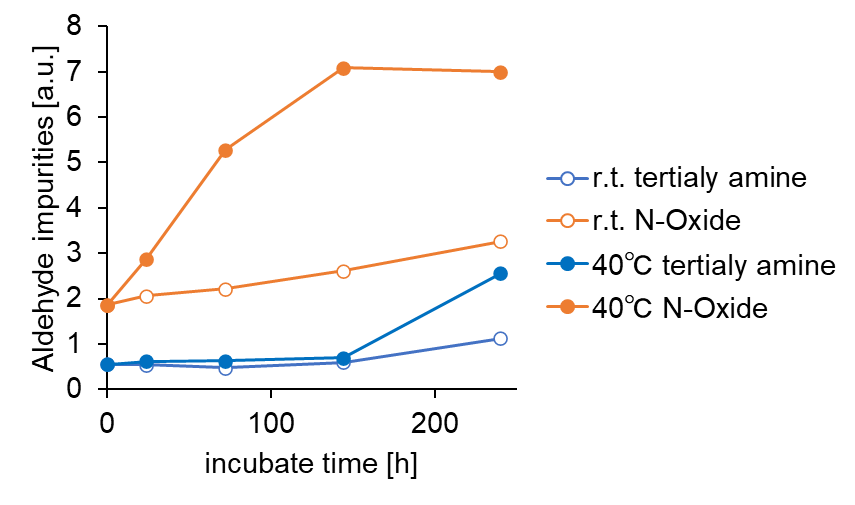
**

**Figure S7. *N*-oxidized ionizable lipids are the main drivers of aldehyde production**

CL4F16-1 (4 mM) or corresponding *N*-oxide ethanol solution was incubated with 50 mM sodium acetate (pH 5.3) at a ratio of 1:3 at room temperature or 40 °C; 285 µL of NBD-H solution was added to 15 µL of the samples and incubated for 60 min at 25 °C. After incubation, the fluorescence intensity was measured.


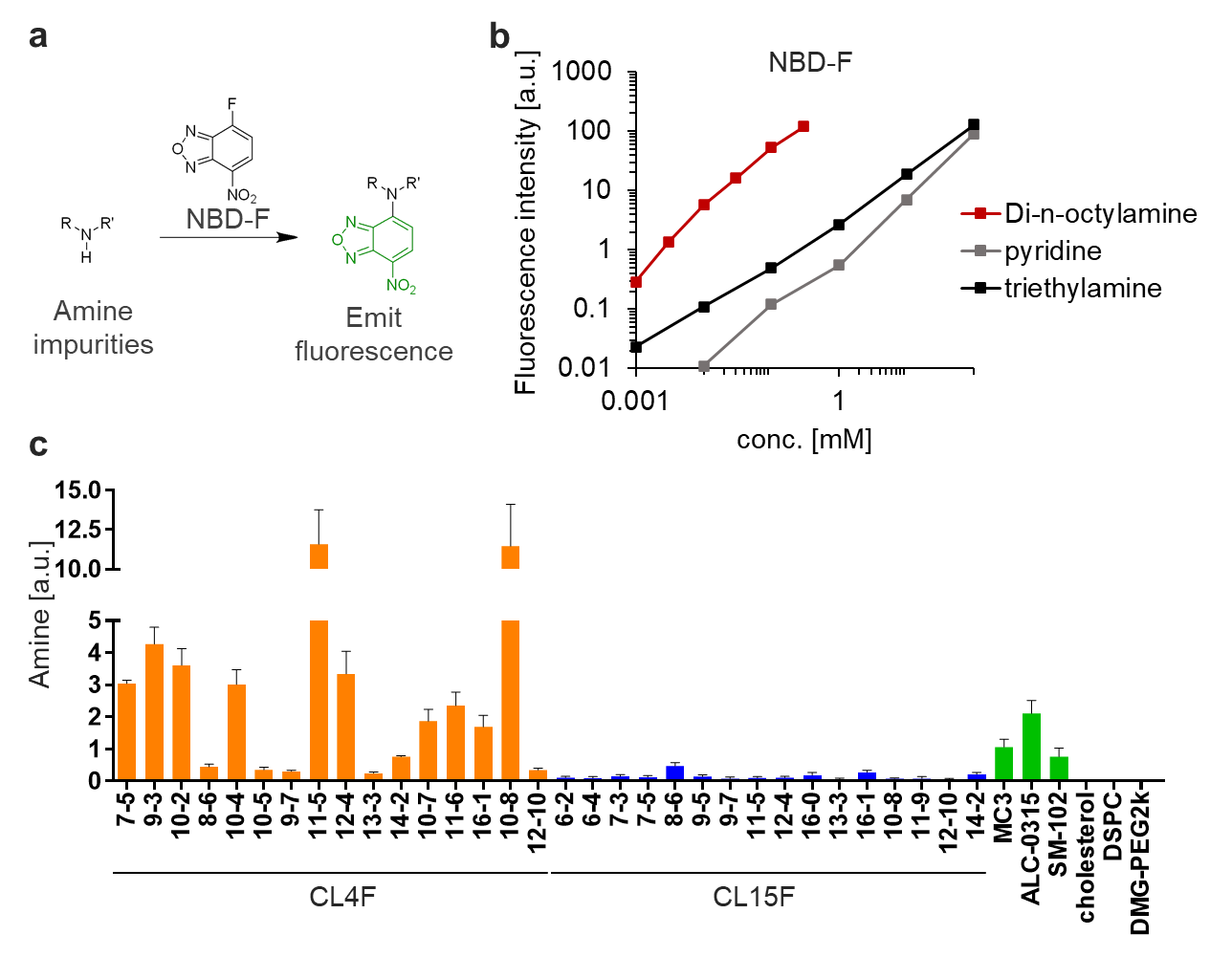


**Figure S8. NBD-F emits fluorescence in response to primary and secondary amines**

a) NBD-F (4-fluoro-7-nitro-2,1,3-benzoxadiazole) reacts with primary or secondary amine impurities in ionizable lipids to emit fluorescence^1^. b) NBD-F was diluted with acetonitrile to a final concentration of 100 mM; 10 µL of NBD-F solution was added to 30 µL of 4 mM lipid in ethanol and incubated for 1 min at 60 °C. After incubation, the samples were cooled on ice; this was followed by the addition of 150 µL of 50 mM hydrochloric acid ethanol solution. Fluorescence intensity was measured using a Varioskan LUX Multimode Microplate Reader (Thermo Fisher Scientific, MA, USA) with an excitation wavelength of 470 nm and an emission wavelength of 530 nm in black 96-well plates in a total volume of 150 µL. When mixed with NBD-F, triethylamine emitted a slight fluorescence, probably because of contamination with a small amount of dipropylamine and isopropylethylamine in the product. c) Relative amount indicating that almost all CL15F lipids had a small amount of amine impurities compared to CL4F lipids and other ionizable lipids. Mean ± SD.


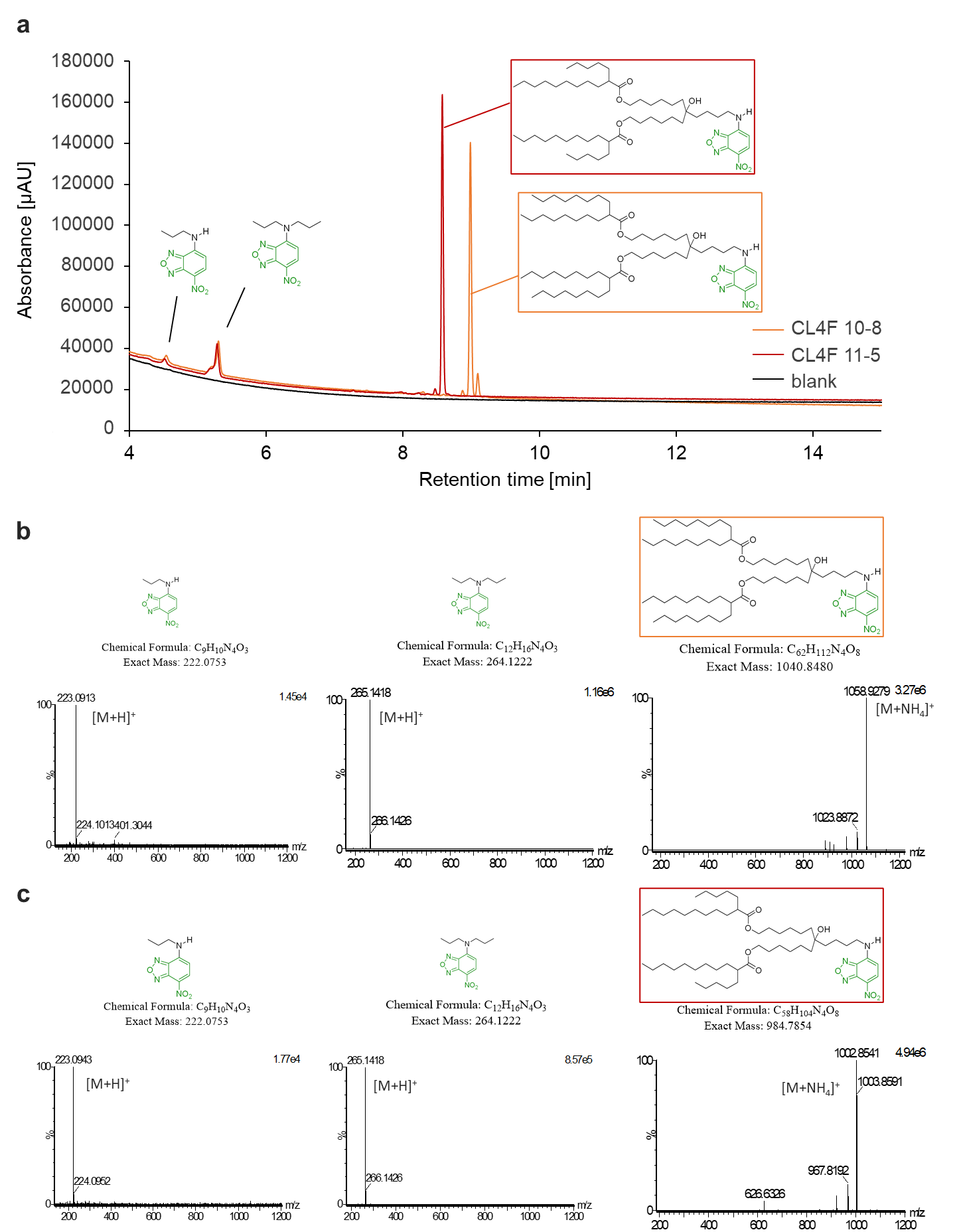


**Figure S9. Identification of NBD-F-labelled compounds in CL4F 10-8 and CL4F 11-5**

a) For primary or secondary amine labelling, 1 µL of lipid ethanol solution was added to 0.5 mL of 1.09 mM NBD-F ethanol solution and 0.5 mL of 1.0 mM NaOH ethanol solution and incubated for 5 min at 60 °C for derivatization. The sample was analyzed using HPLC after cooling on ice for 5 min and acidification with 4 mL of 20 mM hydrochloric acid ethanol solution. Chromatographic analysis was carried out using an Acquity UPLC system (Waters). Separation was achieved on an Acquity UPLC BEH C4 analytical column (100 mm × 2.1 mm, 1.7 μm, Waters) at a column temperature of 50 °C. Mobile phase A was 10 mM ammonium acetate and mobile phase B was acetonitrile. Separation was accomplished using a step-gradient with a 3.5-min gradient from 60–95% B and hold at 95% B, delivered at a flow rate of 0.2 mL/min with an injection volume of 2 μL. The labelled amine was detected using UV at 490 nm. CL4F 10-8 with NBD-F label (orange), CL4F 11-5 with NBD-F label (red), and NBD-F baseline (black). Fatty secondary amines from CL4F 10-8 and CL4F 11-5 eluted at 9.0 and 8.6 min, respectively. b, c) Full MS scans (ESI-positive) were acquired in the Xevo G2-XS QTOF over m/z 50–2000 range. High resolution mass measurement, along with the pattern of isotopes, allowed for detecting the exact chemical composition of NBD-F-labelled compounds in CL4F 10-8 (b) and CL4F 11-5 (c) with high reliability.

**
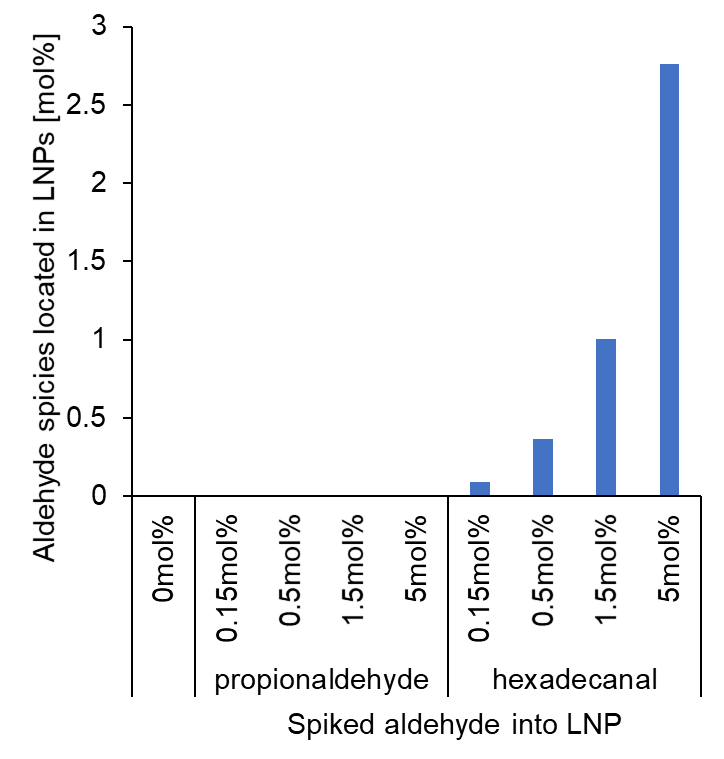
**

**Figure S10. Fatty aldehydes can localize in mRNA/LNPs**

CL15F 10-8 LNPs were formulated after spiking propionaldehyde and hexadecanal into the lipid mixture at different concentrations. The amount of residual aldehyde in the purified mRNA/LNPs was evaluated using NBD-H, as described above. Hexadecanal was trapped inside the LNPs, whereas propionaldehyde was not. The fluorescence intensity derived from NBD-H increased with increasing aldehyde spike levels. These data indicated that fatty aldehyde species localize in mRNA/LNPs, whereas hydrophilic aldehydes do not.

**
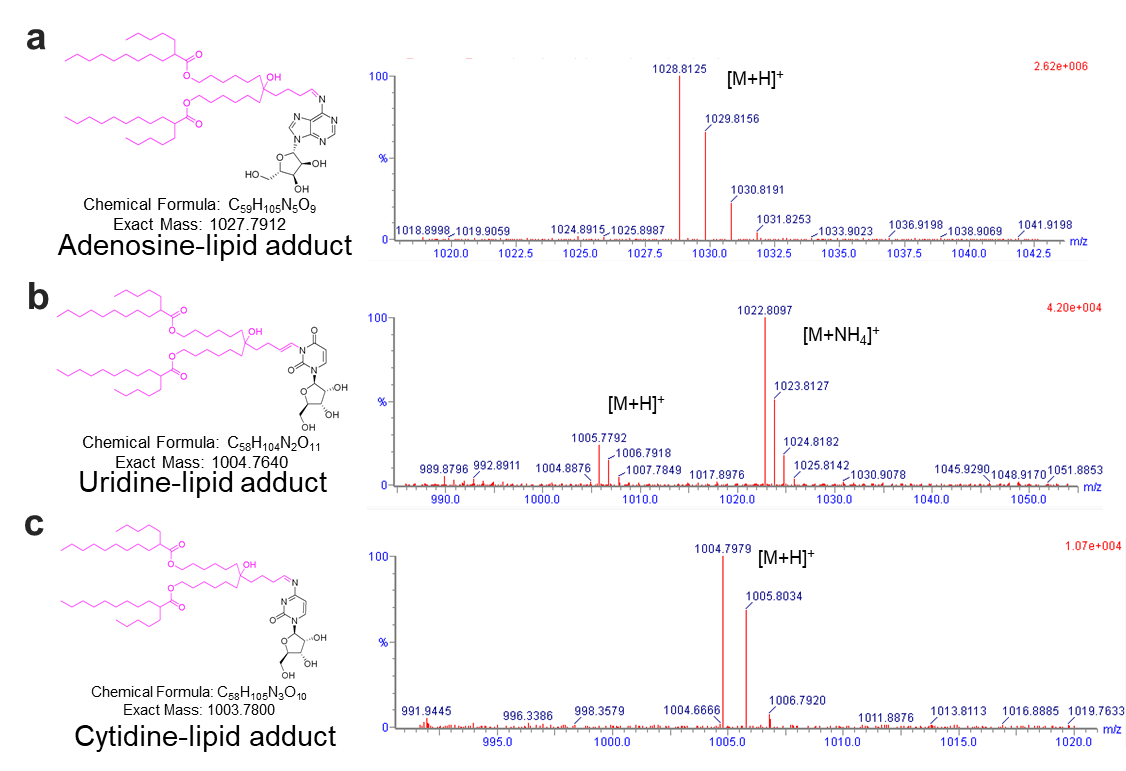
**

**Figure S11. Identification of aldehyde-modified nucleosides in CL4F 11-5**

a–c) Chromatographic analysis was performed using the Acquity UPLC system (Waters, Milford, MA, USA) to monitor the modification of nucleosides with lipid-derived impurities in a simplified system. Separation was achieved on an Acquity UPLC BEH Amide analytical column (100 mm × 2.1 mm, 1.7 μm, Waters, USA) at a column temperature of 40 °C. Full MS scans (ESI-positive) were acquired using Waters ZQ2000 over an m/z range of 50–2000 with a 30 V cone voltage. High-resolution mass spectrometry, along with isotope patterns, allowed for determining the exact chemical composition of aldehyde-modified nucleosides with high reliability.

**
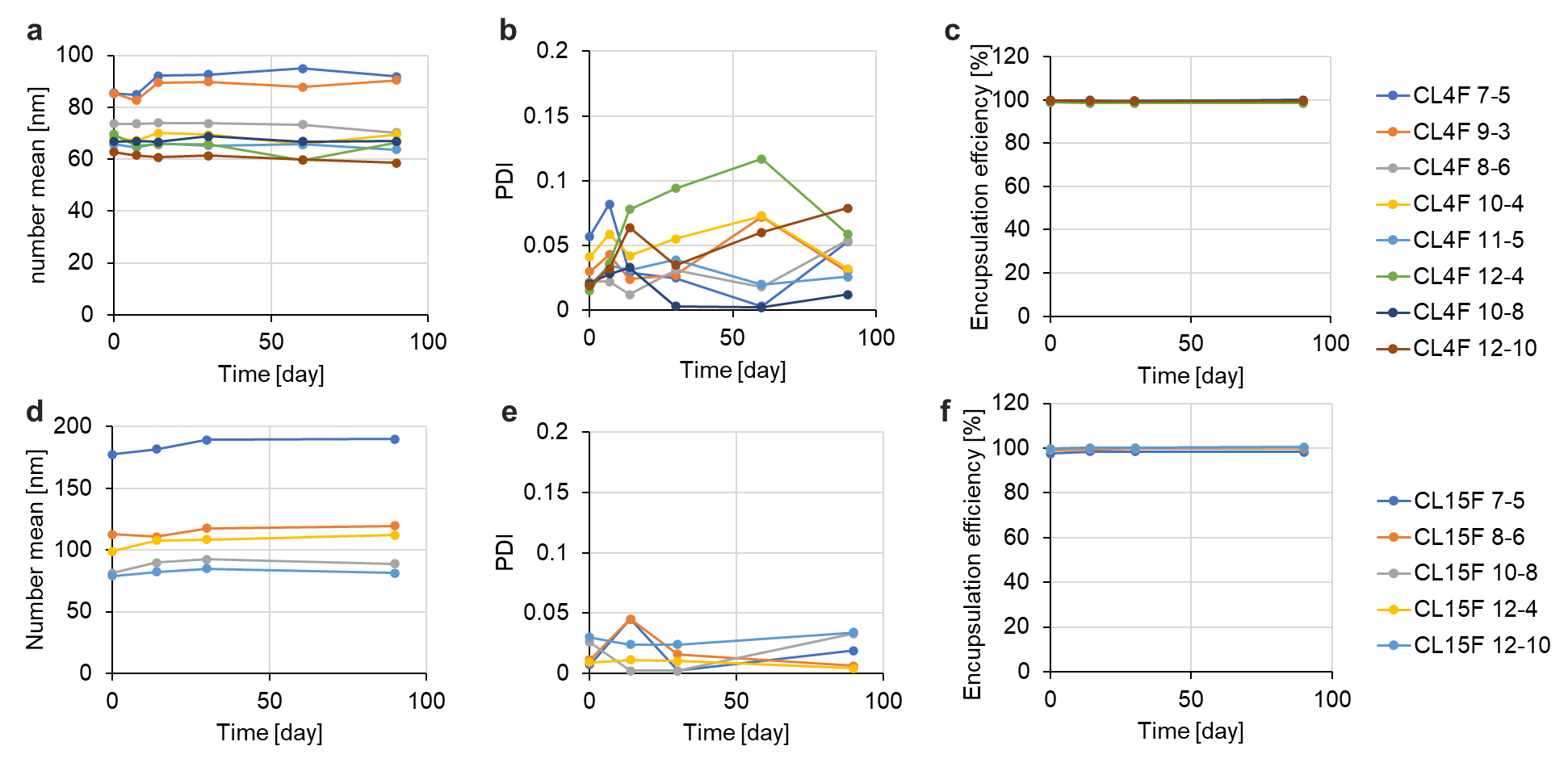
**

**Figure S12. Physicochemical properties of Fluc mRNA carrying LNPs are maintained for at least 3 months when stored at 4 °C**

a–c) Particle size, PDI, and encapsulation efficiency of CL4F LNPs. d–f) Particle size, PDI, and encapsulation efficiency of CL15F LNPs.

**
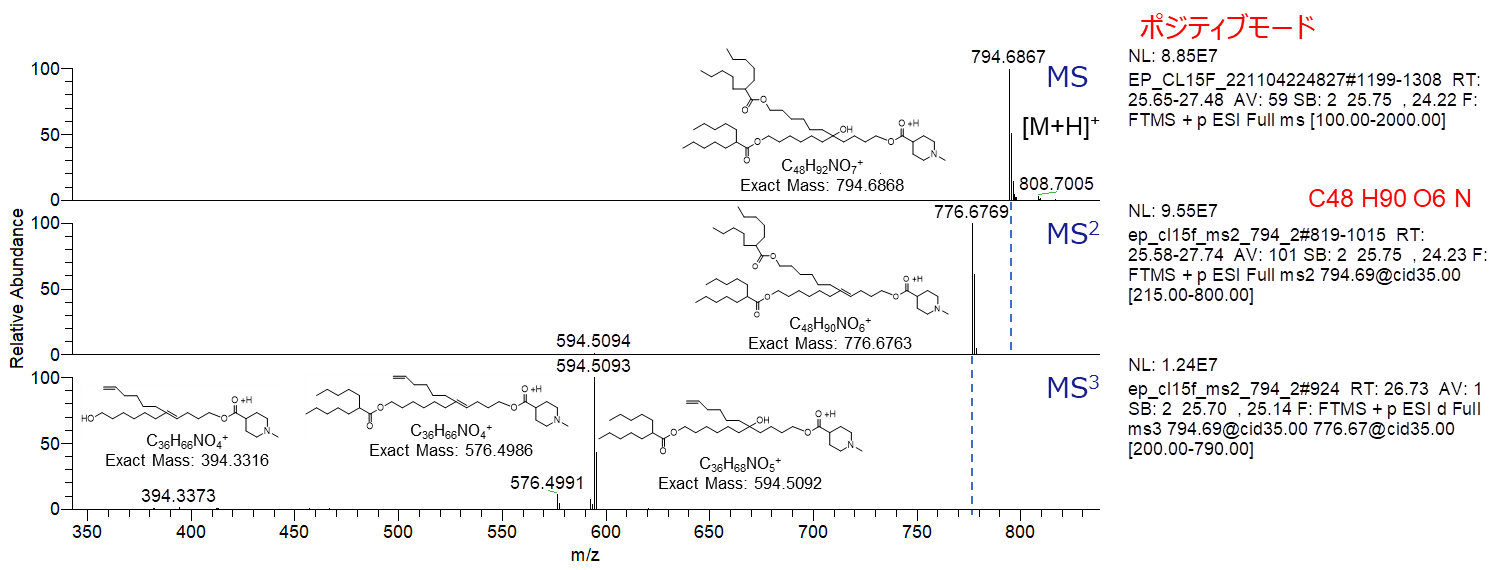
**

**Figure S13. CID MS^n^ spectra of main peaks in CL15F 7-5**

The impurity structure was analyzed using an UltiMate3000 instrument (Thermo Fisher Scientific) with an ACQUITY UPLC BEH C18 Column (130 Å, 1.7 µm, 2.1 mm, 100 mm) and a gradient of 50 to 95% isopropanol/acetonitrile (62:33) in water with 5 mM ammonium acetate over 10 min and held at 95% isopropanol/acetonitrile (62:33) in water with 5 mM ammonium acetate for 10 min at 0.3 mL/min. Injection volume was 1.0 or 0.2 µL and the column temperature was 60 °C. Mass spectral data were acquired using an LTQ Orbitrap XL in the positive electrospray ionization (ESI) mode. The electrospray voltage was 3.0 kV and the ion transfer capillary temperature was 350 °C. Full MS scans were acquired using an Orbitrap mass analyzer over an m/z range of 100–2000 with a resolution of 60,000. CID MS^n^ spectra were acquired using a linear ion trap in an Orbitrap mass analyzer with a resolution of 30000.

**
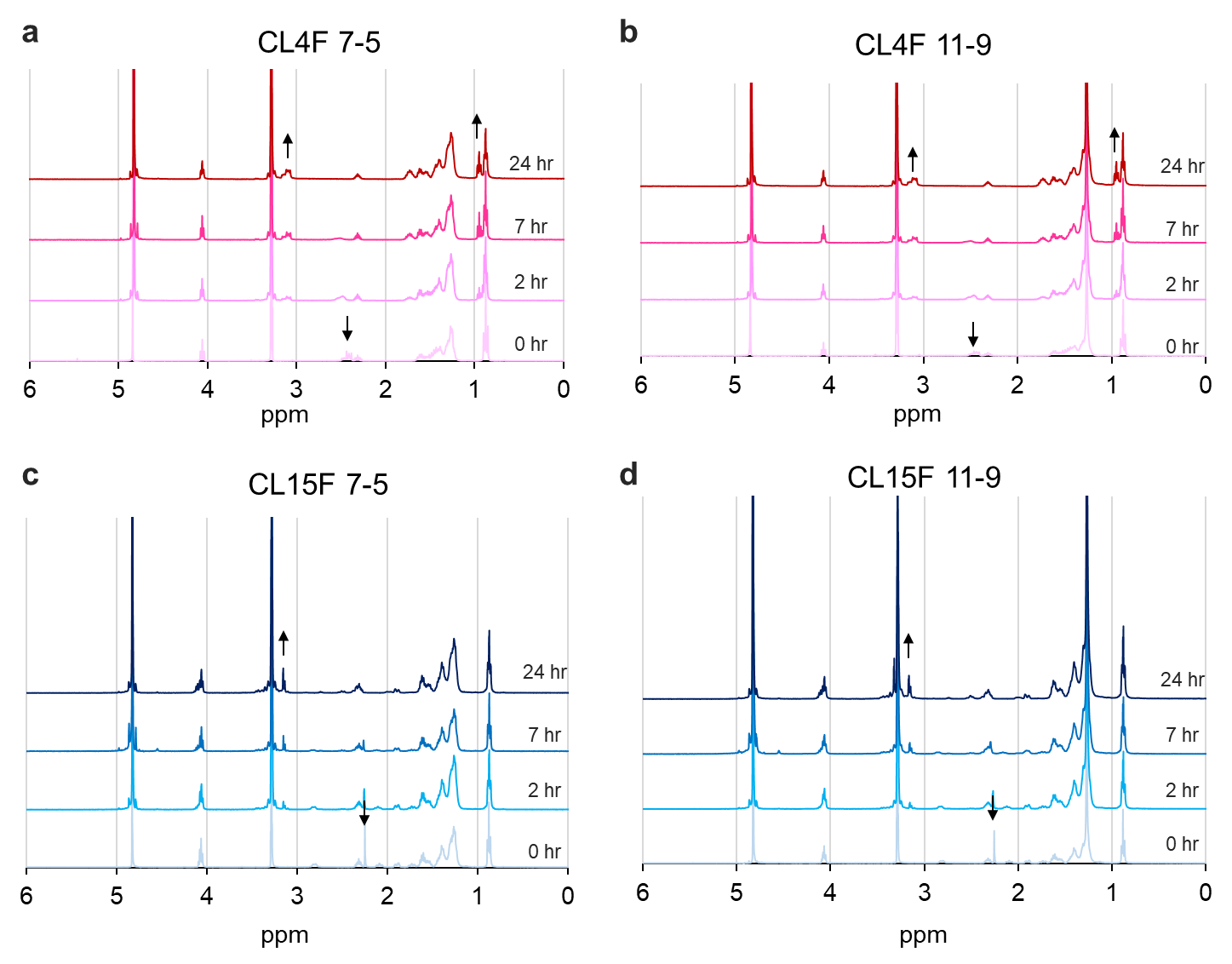
**

**Figure S14. Monitoring oxidation kinetics of CL4F 7-5, CL4F 11-9, CL15F 7-5, and CL15F 11-9 by ^1^H NMR spectroscopy in CD_3_OD at 298 K**

a–d) Four equivalents of hydrogen peroxide was added to CL4F 7-5 (a), CL4F 11-9 (b), CL15F 7-5 (c), and CL15F 11-9 (d) and stirred at 50 °C. ^1^H NMR spectrum of each reaction mixture was acquired using methanol-d_4_. When the oxidation progressed, the peak at 2.40−2.60 ppm corresponded to the methylene group of CL4F lipids. The peak at 2.25−2.30 ppm corresponding to the methyl group attached to the nitrogen atom of CL15F lipids disappeared, and the peak at 3.05−3.18 ppm corresponding to the methyl/methylene group attached to the oxidized nitrogen atom appeared, as indicated by the arrows.

**
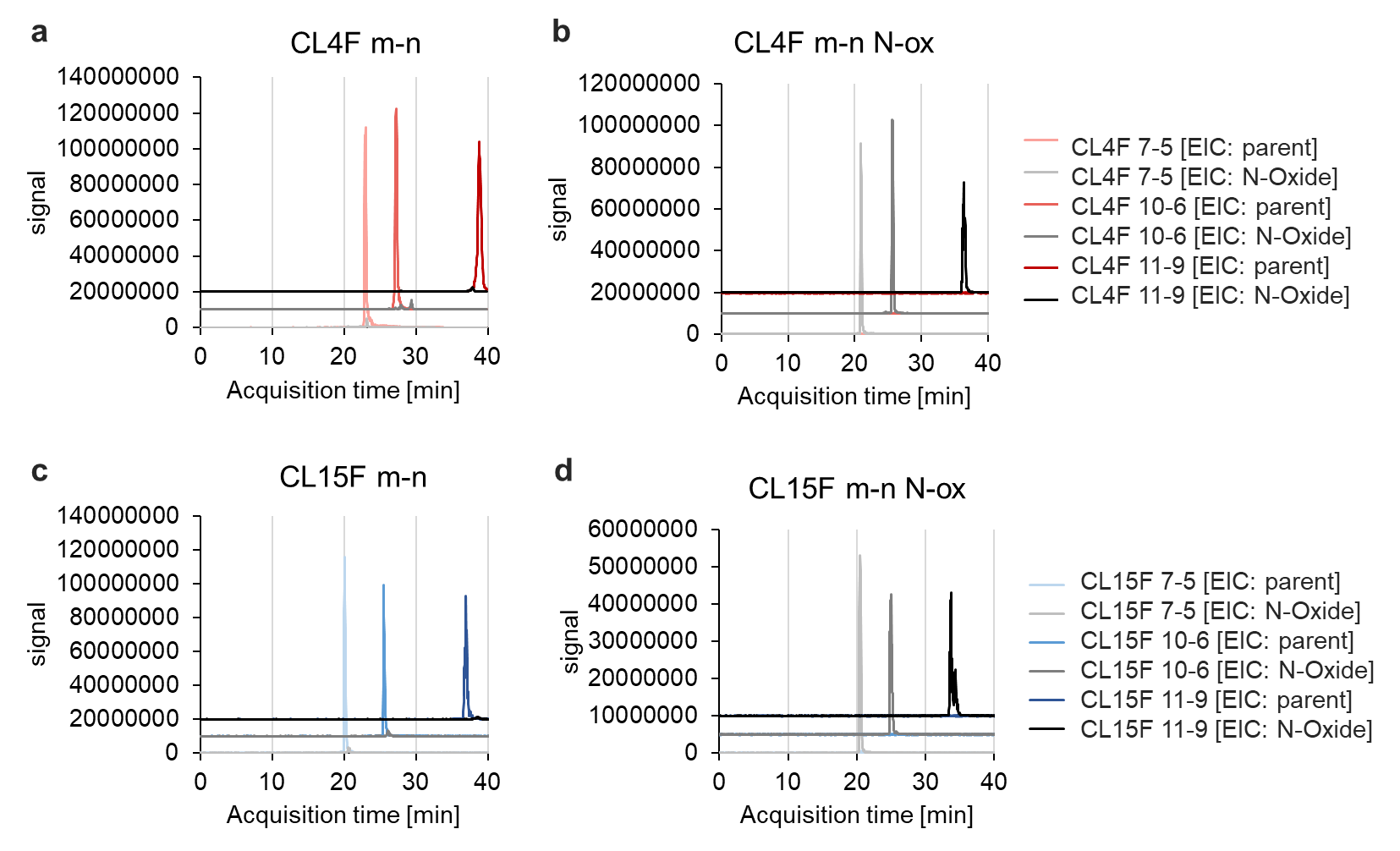
**

**Figure S15. LC/MS verified the oxidation of ionizable lipids**

a–d) EICs of selected m/z values corresponding to each parent lipid and *N*-oxide before (a, c) and after oxidation (b, d). Before oxidation (CL4F m-n and CL15F m-n), the parent lipid was the primary component; *N*-oxide was present at low levels. After oxidation (CL4F m-n *N*-ox and CL15F m-n *N*-ox), only nitrogen oxide was detected.


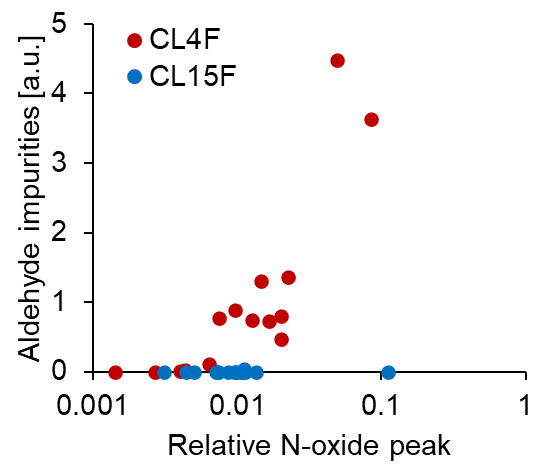


**Figure S16. Regardless of the quantity of oxidized lipids, few aldehyde impurities were detected in CL15F**

For lipids with unsaturated fatty acids, such as MC3, two oxidation processes are assumed: epoxidation of the double bond and oxidation of the polar head. Lipids with saturated branched fatty acids were used in this study; therefore, only the polar head was oxidized. Thus, we compared the peak area of the extracted ion chromatogram of the oxidized form standardized to that of the parent lipid with the relative aldehyde impurity estimated using NBD-H. Aldehyde impurities correlated with the *N*-oxide in CL4F lipids; however, almost no aldehyde impurities were detected, independent of *N*-oxide, in CL15F lipids.

# **Supporting tables**

**Table S1.** Physicochemical properties of the OVA mRNA carrying LNPs

|  | Number mean [nm] | PDI | EE | ζ-potential [mV] |
| --- | --- | --- | --- | --- |
| CL15F 7-5 | 165.0 | 0.028 | 96.9 | -4.27 |
| CL15F 9-3 | 174.9 | 0.013 | 93.3 | -6.97 |
| CL15F 9-5 | 121.2 | 0.004 | 98.7 | -7.42 |
| CL15F 10-6 | 94.8 | 0.002 | 99.3 | -4.93 |
| CL15F 14-2 | 105.3 | 0.082 | 95.8 | -6.23 |
| CL15F 11-7 | 86.1 | 0.002 | 99.6 | -3.27 |
| CL15F 11-9 | 84.9 | 0.033 | 99.5 | -3.49 |
| CL15F 12-4 | 93.4 | 0.055 | 99.1 | -3.30 |
| ALC-0315 | 52.4 | 0.056 | 98.7 | -3.77 |
| SM-102 | 57.1 | 0.052 | 98.5 | 0.80 |

**Table S2.** ζ-Average, polydispersity index (PDI), and mRNA encapsulation efficiency for LNPs carrying hEPO-encoded mRNA

|  |  |  | 4 °C | | -80 °C | |
| --- | --- | --- | --- | --- | --- | --- |
|  | Day | 0 | 30 | 150 | 30 | 150 |
| CL4F 8-6 | Number mean [nm] | 68.35 | 78.87 | 91.80 | 78.59 | 79.18 |
|  | PDI | 0.069 | 0.072 | 0.142 | 0.052 | 0.028 |
|  | EE [%] | 97.49 | 96.23 | 80.11 | 95.37 | 92.59 |
| CL4F 10-4 | Number mean [nm] | 69.29 | 75.91 | 76.58 | 71.69 | 66.79 |
|  | PDI | 0.086 | 0.062 | 0.082 | 0.096 | 0.088 |
|  | EE [%] | 96.92 | 96.22 | 85.78 | 94.24 | 89.19 |
| CL15F 12-10 | Number mean [nm] | 82.08 | 88.32 | 93.48 | 99.16 | 93.49 |
|  | PDI | 0.056 | 0.085 | 0.042 | 0.008 | 0.033 |
|  | EE [%] | 99.04 | 98.62 | 97.65 | 98.06 | 97.13 |
| CL15F 14-12 | Number mean [nm] | 82.68 | 89.56 | 91.75 | 94.48 | 88.86 |
|  | PDI | 0.056 | 0.064 | 0.038 | 0.062 | 0.016 |
|  | EE [%] | 99.23 | 98.54 | 97.88 | 98.15 | 97.62 |
| SM-102 | Number mean [nm] | 60.86 | 67.96 | 68.92 |  |  |
|  | PDI | 0.095 | 0.117 | 0.098 |  |  |
|  | EE [%] | 97.93 | 95.92 | 89.77 |  |  |
| ALC-0315 | Number mean [nm] | 50.84 | 57.32 | 64.49 |  |  |
|  | PDI | 0.099 | 0.108 | 0.123 |  |  |
|  | EE [%] | 97.26 | 97.73 | 89.01 |  |  |

**Table S3.** Physicochemical properties of the FLuc mRNA carrying cyclic LNPs are maintained for at least 2 months when stored at 4 °C.

|  |  | Number mean [nm] | PDI | EE | ζ-potential [mV] |
| --- | --- | --- | --- | --- | --- |
| CL6F 14-12 | Day0 | 65.47 | 0.108 | 98.5 | 8.12 |
|  | Day60 | 62.19 | 0.079 | 98.7 | -10.3 |
| CL16F 14-12 | Day0 | 63.61 | 0.097 | 98.5 | -6.97 |
|  | Day60 | 66.33 | 0.066 | 98.4 | -5.94 |
| CL17F 14-12 | Day0 | 64.14 | 0.071 | 98.3 | -6.76 |
|  | Day60 | 60.68 | 0.075 | 99.1 | -6.18 |

# **Lipid synthesis**

*General information*

All simple chemicals were purchased from Tokyo Chemical Industry (Tokyo, Japan) or FUJIFILM Wako Pure Chemical Corporation (Osaka, Japan) and used without further purification. All reactions were monitored using thin-layer chromatography on precoated thin-layer chromatography (TLC) plates (Millipore) and stained with bromocresol green solution or p-anisaldehyde solution. Products were purified using a Biotage Selekt automated chromatography system. ^1^H NMR spectra were obtained using JEOL ECZ400, ECP400 (Tokyo, Japan) or Bruker AVANCE 300 MHz (MA, USA), and the chemical shifts were expressed in parts per million (ppm) with respect to the residual solvent peak. The following abbreviations are used to express multiplication: s = singlet, d = doublet, t = triplet, and m = multiplet. All final compounds were identified by reverse-phase UPLC-MS using a Waters Acquity UPLC instrument with an ACQUITY UPLC BEH C18 Column (130 Å, 1.7 µm, 2.1 mm, 100 mm) and a gradient of 50 to 95% isopropanol/acetonitrile (62:33) in water with 5 mM ammonium acetate over 20 min and held at 95% isopropanol/acetonitrile (62:33) in water with 5 mM ammonium acetate for 20 min at 0.2 mL/min. Injection volume was 1.0 µL and the column temperature was 40 °C. To analyze long tail lipids such as 12-10 and 14-12, the column temperature was set at 60 °C. Mass spectral data were acquired using an ACQUITY QDa in the positive electrospray ionization (ESI) mode. All compounds were provided as mixtures of all possible stereoisomers.

*Synthesis of CL15F m-n lipids*

*
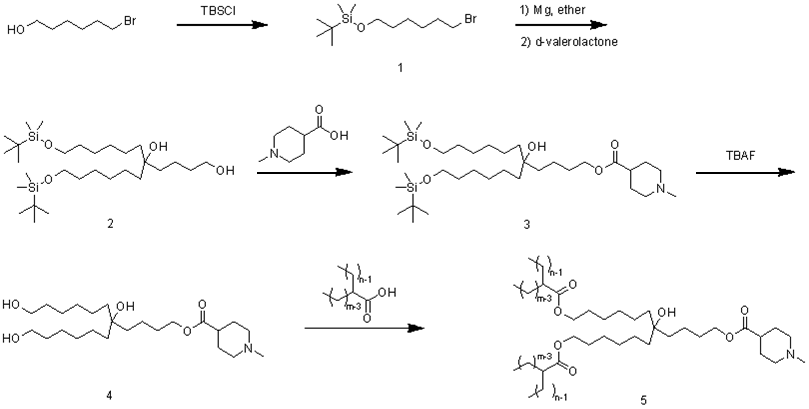
*

*Synthesis of ((6-bromohexyl)oxy)(tert-butyl)dimethylsilane (1)*

6-Bromo-1-hexanol (50.6 g, 279 mmol) was dissolved in anhydrous DCM (300 mL), and the mixture was cooled on ice. TEA (59.7 mL, 431 mmol) and tert-butyldimethylchlorosilane (TBSCl) (45.8 g, 304 mmol) were added to the mixture. The resulting mixture was stirred overnight at ambient temperature. After the evaporation of the reaction mixture, the residue was diluted with ethyl acetate and washed with brine. The organic phase was then dried over Na_2_SO_4_. Evaporation of the solvent gave 70.0 g (85%) of compound 1 as a yellow oily residue.

^1^H NMR (400 MHz, CDCl_3_, ppm) δ: 0.04 (s, 6H), 0.89 (s, 9H), 1.31-1.55 (m, 6H), 1.86 (m, 2H), 3.40 (t, 2H), 3.60 (t, 2H).

*Synthesis of 11-((tert-butyldimethylsilyl)oxy)-5-(6-((tert-butyldimethylsilyl)oxy)hexyl)undecane-1,5-diol (2)*

A solution of compound 1 (70.0 g, 237 mmol) in 75 mL of ether was added dropwise to a suspension of Mg turnings (6.3 g, 259 mmol) with three crystals of iodine in 15 mL of anhydrous ether under argon while maintaining the reaction under gentle reflux. The resulting mixture was stirred at ambient temperature for 2 h. The mixture was cooled to 0° C. The cloudy mixture was added dropwise at 0 °C in a solution of δ-valerolactone (10.92 g, 109 mmol) in 10.0 mL of ether. An exothermic reaction was observed, and the reaction mixture was refluxed. The mixture was stirred at ambient temperature for 30 min. The reaction was quenched by adding an aqueous solution of saturated citric acid (100 mL) dropwise at 0 °C. The organic phase was washed with water and brine and dried over anhydrous Na_2_SO_4_. Evaporation of the solvent yielded a yellow oily residue. The residue was purified using flash chromatography (SiO_2_, dichloromethane/MeOH). This gave 46.0 g (79.2%) of compound 2 as a colorless oil.

^1^H NMR (400 MHz, CDCl_3_, ppm) δ: 0.04 (s, 12H), 0.88 (s, 18H), 1.21-1.60 (m, 26H), 3.59 (t, 4H), 3.66 (t, 2H).

*Synthesis of 11-((tert-butyldimethylsilyl)oxy)-5-(6-((tert-butyldimethylsilyl)oxy)hexyl)-5-hydroxyundecyl 1-methylpiperidine-4-carboxylate (3)*

Compound 2 (14.7 g, 27.5 mmol) was dissolved in anhydrous DCM (150 mL) and 1-methylpiperidine-4-carboxylic acid hydrochloride (5.434 g, 30.25 mmol); dimethylaminopyridine (DMAP) (336 mg, 2.75 mmol) and EDCI (6.33 g, 33.0 mmol) were added to this mixture. The reaction mixture was stirred overnight at ambient temperature. After evaporation of the reaction mixture, the residue was suspended in ethyl acetate (200 mL) and washed with NaOH solution (0.5 M, 200 mL) and brine (100 mL). The organic phase was then dried over Na_2_SO_4_. Evaporation of the solvent gave compound 3 (20.0 g) as a pale-yellow oily residue.

^1^H NMR (400 MHz, CDCl_3_, ppm) δ: 0.04 (s, 12H), 0.88 (s, 18H), 1.15-2.10 (m, 32H), 2.23 (m, 4H), 2.78 (m, 2H), 3.57 (t, 4H), 4.08 (t, 2H).

*Synthesis of 5,11-duhydroxy-5-(6-hydroxyhexyl)undecyl 1-methylpiperidine-4-carboxylate (4)*

Acetic acid (5.72 mL, 100 mmol) and tetrabutylammonium fluoride (TBAF) in THF (1.0 M, 80 mL) were added to compound 3 (20.0 g, 30.4 mmol) at 0 °C. The reaction mixture was stirred overnight at room temperature. After evaporation of the reaction mixture, the residue was suspended in an aqueous solution of saturated citric acid and washed with dichloromethane. After the aqueous phase was diluted with ethyl acetate, compound 4 was extracted into the organic phase by basification of the aqueous layer with 5 N NaOH. The organic phase was washed with brine and dried over sodium sulfate (Na_2_SO_4_). Evaporation of the solvent gave 18.7 g crude product as a yellow oily residue. The residue was purified using flash chromatography (SiO_2_, dichloromethane/MeOH). This resulted in 7.0 g (54.0%) of compound 4 as a colorless oil.

^1^H NMR (400 MHz, CDCl_3_, ppm) δ: 1.25-1.78 (m, 28H), 1.89 (m, 2H), 2.09 (t, 2H), 2.25 (s, 3H), 2.33 (m, 1H), 2.82 (m, 2H), 3.62 (t, 4H), 4.08 (t, 2H).

*Synthesis of CL15F 6-2 (5)*

Branched fatty acids were synthesized, as previously described^2^. 2-Ethylhexanoic acid (6-2) as a fatty acid (2.4 mmol) was added to 5,11-duhydroxy-5-(6-hydroxyhexyl)undecyl 1-methylpiperidine-4-carboxylate (430 mg, 1.0 mmol) in anhydrous DCM (5 mL). DMAP (12.2 mg, 0.1 mmol) and EDCI-HCl (576 mg, 3.0 mmol) were added to the mixture and the reaction mixture was stirred at 25 °C overnight. After evaporation of the solvent, the residue was suspended in ethyl acetate, washed with a 0.5 N NaOH solution, and then brine. The organic phase was then dried over Na_2_SO_4_. Evaporation of the solvent yielded a crude yellow oily residue. The residue was purified using flash chromatography [ODS, H_2_O (0.1% trifluoroacetic acid)/acetonitrile:isopropanol = 50:50 (0.1% TFA)] and (SiO_2_, DCM/MeOH). This gave CL15F 6-2 (360 mg, 52.7%) as a colorless oil.

^1^H NMR (400 MHz, CD_3_OD, ppm) δ: 0.87 (m, 12H), 1.25-1.65 (m, 43H), 1.73 (m, 2H), 1.88 (m, 2H), 2.09 (m, 2H), 2.23 (m, 5H), 2.33 (m, 1H), 2.80 (m, 2H), 4.07 (m, 6H). UPLC/CAD: RT = 12.74 min. MS (ESI): m/z calculated for C_40_H_76_NO_7_ (M+H)^+^, 682.56; found, 682.55.

*Synthesis of CL15F 6-4 (6)*

Using a procedure analogous to that described for the synthesis of CL15F 6-2 (5), CL15F 6-4 (6) (318 mg, 64.9%) was obtained as a colorless oil from 2-buthylhexanoic acid (6-4) as a fatty acid (1.59 mmol).

^1^H NMR (400 MHz, CD_3_OD, ppm) δ: 0.88 (m, 12H), 1.22-1.65 (m, 51H), 1.74 (t, 2H), 1.89 (d, 2H), 2.09 (t, 2H), 2.25 (s, 3H), 2.32 (m, 3H), 2.80 (m, 2H), 4.08 (m, 6H).

UPLC/CAD: RT = 16.91 min. MS (ESI): m/z calcd for C_44_H_84_NO_7_ (M+H)^+^, 738.62; found, 738.61.

*Synthesis of CL15F 7-3 (7)*

Using a procedure analogous to that described for the synthesis of CL15F 6-2 (5), CL15F 7-3 (7) (429 mg, 58.1%) was obtained as a colorless oil from 2-propylheptanoic acid (7-3) as a fatty acid (2.4 mmol).

^1^H NMR (400 MHz, CD_3_OD ppm) δ: 0.89 (m, 12H), 1.22-1.65 (m, 51H), 1.74 (t, 2H), 1.89 (d, 2H), 2.09 (t, 2H), 2.25 (s, 3H), 2.34 (m, 3H), 2.79 (d, 2H), 4.06 (m, 6H).

UPLC/CAD: RT = 17.49 min. MS (ESI): m/z calcd for C_44_H_84_NO_7_ (M+H)^+^, 738.62; found, 738.65.

*Synthesis of CL15F 7-5 (8)*

Using a procedure analogous to that described for the synthesis of CL15F 6-2 (5), CL15F 7-5 (8) (480 mg, 60.4%) was obtained as a colorless oil from 2-pentylheptanoic acid (7-5) as a fatty acid (2.4 mmol).

^1^H NMR (400 MHz, CD_3_OD, ppm) δ: 0.88 (m, 12H), 1.22-1.62 (m, 59H), 1.74 (t, 2H), 1.89 (d, 2H), 2.09 (t, 2H), 2.25 (s, 3H), 2.34 (m, 3H), 2.80 (d, 2H), 4.08 (m, 6H).

UPLC/CAD: RT = 20.05 min. MS (ESI): m/z calcd for C_48_H_92_NO_7_ (M+H)^+^, 794.69; found, 794.71.

*Synthesis of CL15F 9-3 (9)*

Using a procedure analogous to that described for the synthesis of CL15F 6-2 (5), CL15F 9-3 (9) (273 mg, 39.4%) was obtained as a colorless oil from 2-propylnonanoic acid (9-3) as a fatty acid (2.10 mmol).

^1^H NMR (300 MHz, CD_3_OD, ppm) δ: 0.91 (m, 12H), 1.28-1.67 (m, 59H), 1.75 (t, 2H), 1.90 (d, 2H), 2.10 (t, 2H), 2.27 (s, 3H), 2.37 (m, 3H), 2.80 (d, 2H), 4.08 (m, 6H).

UPLC/CAD: RT = 20.73 min. MS (ESI): m/z calcd for C_48_H_92_NO_7_ (M+H)^+^, 794.69; found, 794.68.

*Synthesis of CL15F 8-6 (10)*

Using a procedure analogous to that described for the synthesis of CL15F 6-2 (5), CL15F 8-6 (10) (580 mg, 68.2%) was obtained as a colorless oil from 2-hexyloctanoic acid (8-6) as a fatty acid (2.4 mmol).

^1^H NMR (400 MHz, CD_3_OD, ppm) δ: 0.87 (m, 12H), 1.25-1.64 (m, 67H), 1.81 (m, 2H), 1.95 (m, 2H), 2.10 (m, 2H), 2.31 (m, 6H), 2.84 (d, 2H), 4.08 (m, 6H).

UPLC/CAD: RT = 23.39 min. MS (ESI): m/z calcd for C_52_H_100_NO_7_ (M+H)^+^, 850.75; found, 850.77.

*Synthesis of CL15F 9-5 (11)*

Using a procedure analogous to that described for the synthesis of CL15F 6-2 (5), CL15F 9-5 (11) (390 mg, 64.6%) was obtained as a colorless oil from 2-pentylnonanoic acid (9-5) as a fatty acid (1.70 mmol).

^1^H NMR (400 MHz, CD_3_OD, ppm) δ: 0.89 (m, 12H), 1.20-1.67 (m, 67H), 1.72 (t, 2H), 1.90 (d, 2H), 2.09 (t, 2H), 2.25 (s, 3H), 2.32 (m, 3H), 2.80 (d, 2H), 4.08 (m, 6H).

UPLC/CAD: RT = 23.33 min. MS (ESI): m/z calcd for C_52_H_100_NO_7_ (M+H)^+^, 850.75; found, 850.74.

*Synthesis of CL15F 10-4 (12)*

Using a procedure analogous to that described for the synthesis of CL15F 6-2 (5), CL15F 10-4 (12) (221 mg, 43.2%) was obtained as a colorless oil from 2-butyldecanoic acid (10-4) as a fatty acid (1.45 mmol).

^1^H NMR (300 MHz, CD_3_OD, ppm) δ: 0.90 (m, 12H), 1.20-1.67 (m, 67H), 1.72 (t, 2H), 1.90 (d, 2H), 2.09 (t, 2H), 2.27 (s, 3H), 2.34 (m, 3H), 2.80 (d, 2H), 4.08 (m, 6H).

UPLC/CAD: RT = 22.99 min. MS (ESI): m/z calcd for C_52_H_100_NO_7_ (M+H)^+^, 850.75; found, 850.77.

*Synthesis of CL15F 10-5 (13)*

Using a procedure analogous to that described for the synthesis of CL15F 6-2 (5), CL15F 10-5 (13) (352 mg, 40.1%) was obtained as a colorless oil from 2-pentyldecanoic acid (10-5) as a fatty acid (2.4 mmol).

^1^H NMR (300 MHz, CD_3_OD ppm) δ: 0.90 (m, 12H), 1.25-1.67 (m, 71H), 1.75 (t, 2H), 1.90 (d, 2H), 2.10 (t, 2H), 2.27 (s, 3H), 2.36 (m, 3H), 2.80 (d, 2H), 4.09 (m, 6H).

UPLC/CAD: RT = 24.01 min. MS (ESI): m/z calcd for C_54_H_104_NO_7_ (M+H)^+^, 878.78; found, 878.79.

*Synthesis of CL15F 9-7 (14)*

Using a procedure analogous to that described for the synthesis of CL15F 6-2 (5), CL15F 9-7 (14) (315 mg, 54.6%) was obtained as a colorless oil from 2-heptylnonanoic acid (9-7) as a fatty acid (1.53 mmol).

^1^H NMR (400 MHz, CD_3_OD, ppm) δ: 0.88 (m, 12H), 1.20-1.67 (m, 75H), 1.73 (t, 2H), 1.90 (d, 2H), 2.10 (t, 2H), 2.26 (s, 3H), 2.33 (m, 3H), 2.80 (d, 2H), 4.08 (m, 6H).

UPLC/CAD: RT = 25.92 min. MS (ESI): m/z calcd for C_56_H_108_NO_7_ (M+H)^+^, 906.81; found, 906.80.

*Synthesis of CL15F 12-4 (15)*

Using a procedure analogous to that described for the synthesis of CL15F 6-2 (5), CL15F 12-4 (15) (390 mg, 64.9%) was obtained as a colorless oil from 2-butyldodecanoic acid (12-4) as a fatty acid (1.59 mmol).

^1^H NMR (400 MHz, CD_3_OD, ppm) δ: 0.88 (m, 12H), 1.20-1.67 (m, 75H), 1.73 (t, 2H), 1.91 (d, 2H), 2.18 (t, 2H), 2.31 (m, 6H), 2.86 (d, 2H), 4.07 (m, 6H).

UPLC/CAD: RT = 26.19 min. MS (ESI): m/z calcd for C_56_H_108_NO_7_ (M+H)^+^, 906.81; found, 906.80.

*Synthesis of CL15F 11-5 (16)*

Using a procedure analogous to that described for the synthesis of CL15F 6-2 (5), CL15F 11-5 (16) (351 mg, 58.8%) was obtained as a colorless oil from 2-pentylundecanoic acid (11-5) as a fatty acid (1.58 mmol).

^1^H NMR (400 MHz, CD_3_OD, ppm) δ: 0.89 (m, 12H), 1.20-1.65 (m, 75H), 1.73 (t, 2H), 1.90 (d, 2H), 2.09 (t, 2H), 2.26 (s, 3H), 2.32 (m, 3H), 2.80 (d, 2H), 4.08 (m, 6H).

UPLC/CAD: RT = 25.65 min. MS (ESI): m/z calcd for C_56_H_108_NO_7_ (M+H)^+^, 906.81; found, 906.80.

*Synthesis of CL15F 16-0 (17)*

Using a procedure analogous to that described for the synthesis of CL15F 6-2 (5), CL15F 16-0 (17) (485 mg, 75.2%) was obtained as a white powder from hexadecanoic acid (16-0) as a fatty acid (1.71 mmol).

^1^H NMR (400 MHz, CD_3_OD, ppm) δ: 0.88 (t, 6H), 1.20-1.65 (m, 79H), 1.72 (t, 2H), 1.90 (d, 2H), 2.15 (t, 2H), 2.25-2.40 (s, 8H), 2.85 (d, 2H), 4.07 (m, 6H).

UPLC/CAD: RT = 27.32 min. MS (ESI): m/z calcd for C_56_H_108_NO_7_ (M+H)^+^, 906.81; found, 906.80.

*Synthesis of CL15F 13-3 (18)*

Using a procedure analogous to that described for the synthesis of CL15F 6-2 (5), CL15F 13-3 (18) (230 mg, 45.6%) was obtained as a colorless oil from 2-propyltridecanoic acid (13-3) as a fatty acid (1.33 mmol).

^1^H NMR (400 MHz, CD_3_OD, ppm) δ: 0.88 (m, 12H), 1.20-1.65 (m, 75H), 1.79 (m, 2H), 1.99 (d, 2H), 2.34 (m, 2H), 2.47 (m, 6H), 3.02 (d, 2H), 4.08 (m, 6H).

UPLC/CAD: RT = 26.26 min. MS (ESI): m/z calcd for C_56_H_108_NO_7_ (M+H)^+^, 906.81; found, 906.80.

*Synthesis of CL15F 11-6 (19)*

Using a procedure analogous to that described for the synthesis of CL15F 6-2 (5), CL15F 11-6 (5) (396 mg, 52.9%) was obtained as a colorless oil from 2-hexylundecanoic acid (11-6) as a fatty acid (1.92 mmol).

^1^H NMR (300 MHz, CD_3_OD, ppm) δ: 0.90 (m, 12H), 1.20-1.65 (m, 79H), 1.75 (t, 2H), 1.90 (d, 2H), 2.10 (t, 2H), 2.27 (s, 3H), 2.34 (m, 3H), 2.81 (d, 2H), 4.09 (m, 6H).

UPLC/CAD: RT = 27.59 min. MS (ESI): m/z calcd for C_58_H_112_NO_7_ (M+H)^+^, 934.84; found, 934.89.

*Synthesis of CL15F 16-1 (20)*

Using a procedure analogous to that described for the synthesis of CL15F 6-2 (5), CL15F 16-1 (20) (250 mg, 40.8%) was obtained as a colorless oil from 2-methylhexadecanoic acid (16-1) as a fatty acid (1.57 mmol).

^1^H NMR (400 MHz, CD_3_OD, ppm) δ: 0.88 (t, 6H), 1.11 (d, 6H), 1.22-1.65 (m, 79H), 1.73 (t, 2H), 1.90 (d, 2H), 2.10 (t, 2H), 2.26 (s, 3H), 2.30-2.43 (m, 3H), 2.80 (d, 2H), 4.08 (m, 6H).

UPLC/CAD: RT = 29.33 min. MS (ESI): m/z calcd for C_58_H_112_NO_7_ (M+H)^+^, 934.84; found, 934.85.

*Synthesis of CL15F 10-8 (21)*

Using a procedure analogous to that described for the synthesis of CL15F 6-2 (5), CL15F 10-8 (21) (520 mg, 69.6%) was obtained as a colorless oil from 2-octyldecanoic acid (10-8) as a fatty acid (1.86 mmol).

^1^H NMR (400 MHz, CD_3_OD, ppm) δ: 0.89 (m, 12H), 1.21-1.65 (m, 83H), 1.73 (t, 2H), 1.90 (d, 2H), 2.10 (t, 2H), 2.26 (s, 3H), 2.32 (m, 3H), 2.80 (d, 2H), 4.08 (m, 6H).

UPLC/CAD: RT = 29.64 min. MS (ESI): m/z calcd for C_60_H_116_NO_7_ (M+H)^+^, 962.88; found, 962.90.

*Synthesis of CL15F 11-7 (22)*

Using a procedure analogous to that described for the synthesis of CL15F 6-2 (5), CL15F 11-7 (22) (199 mg, 37.6%) was obtained as a colorless oil from 2-heptylundecanoic acid (11-7) as a fatty acid (1.32 mmol).

^1^H NMR (300 MHz, CD_3_OD, ppm) δ: 0.90 (m, 12H), 1.21-1.65 (m, 83H), 1.75 (t, 2H), 1.90 (d, 2H), 2.11 (t, 2H), 2.27 (s, 3H), 2.34 (m, 3H), 2.81 (d, 2H), 4.09 (m, 6H).

UPLC/CAD: RT = 29.75 min. MS (ESI): m/z calcd for C_60_H_116_NO_7_ (M+H)^+^, 962.88; found, 962.90.

*Synthesis of CL15F 11-9 (23)*

Using a procedure analogous to that described for the synthesis of CL15F 6-2 (5), CL15F 11-9 (23) (290 mg, 53.4%) was obtained as a colorless oil from 2-nonanoylundecanoic acid (11-9) as a fatty acid (1.28 mmol).

^1^H NMR (400 MHz, CD_3_OD, ppm) δ: 0.88 (m, 12H), 1.21-1.67 (m, 91H), 1.73 (t, 2H), 1.90 (d, 2H), 2.10 (t, 2H), 2.26 (s, 3H), 2.32 (m, 3H), 2.80 (d, 2H), 4.08 (m, 6H).

UPLC/CAD: RT = 37.15 min. MS (ESI): m/z calcd for C_64_H_124_NO_7_ (M+H)^+^, 1018.94; found, 1018.92.

*Synthesis of CL15F 12-10 (24)*

Using a procedure analogous to that described for the synthesis of CL15F 6-2 (5), CL15F 12-10 (24) (185 mg, 36.1%) was obtained as a colorless oil from 2-decanoyldodecanoic acid (12-10) as a fatty acid (1.15 mmol).

^1^H NMR (400 MHz, CD_3_OD, ppm) δ: 0.88 (m, 12H), 1.20-1.67 (m, 99H), 1.73 (t, 2H), 1.90 (d, 2H), 2.12 (t, 2H), 2.26 (s, 3H), 2.32 (m, 3H), 2.81 (d, 2H), 4.08 (m, 6H).

^13^C NMR (125 MHz, CDCl_3_) δ: 24.20, 22.76, 23.54, 26.09, 27.54, 28.81, 29.42, 29.57, 29.64, 29.68, 30.01, 31.99, 32.59, 39.28, 45.90, 46.42, 55.03, 64.12, 64.28, 74.18, 175.12, 176.76.

UPLC/CAD: RT = 28.06 min. MS (ESI): m/z calcd for C_68_H_132_NO_7_ (M+H)^+^, 1075.00; found, 1075.08.

*Synthesis of CL15F 14-2 (25)*

Using a procedure analogous to that described for the synthesis of CL15F 6-2 (5), CL15F 14-2 (25) (420 mg, 65.4%) was obtained as a colorless oil from 2-ethyltetradecanoic acid (14-2) as a fatty acid (1.71 mmol).

^1^H NMR (400 MHz, CD_3_OD, ppm) δ: 0.89 (m, 12H), 1.23-1.67 (m, 75H), 1.72 (t, 2H), 1.90 (d, 2H), 2.10 (t, 2H), 2.25-2.39 (m, 6H), 2.80 (d, 2H), 4.08 (m, 6H).

UPLC/CAD: RT = 26.40 min. MS (ESI): m/z calcd for C_56_H_108_NO_7_ (M+H)^+^, 906.81; found, 906.80.

*Synthesis of CL15F 10-6 (26)*

Using a procedure analogous to that described for the synthesis of CL15F 6-2 (5), CL15F 10-6 (26) (485 mg, 53.5%) was obtained as a colorless oil from 2-hexyldecanoic acid (10-6) as a fatty acid (2.4 mmol).

^1^H NMR (400 MHz, CD_3_OD, ppm) δ: 0.88 (m, 12H), 1.22-1.65 (m, 75H), 1.73 (t, 2H), 1.90 (d, 2H), 2.08 (t, 2H), 2.25 (s, 3H), 2.32 (m, 3H), 2.79 (d, 2H), 4.08 (m, 6H).

UPLC/CAD: RT = 25.92 min. MS (ESI): m/z calcd for C_56_H_108_NO_7_ (M+H)^+^, 906.81; found, 906.80.

*Synthesis of CL15F 14-12 (27)*

Using a procedure analogous to that described for the synthesis of CL15F 6-2 (5), CL15F 14-12 (27) (510 mg, 43.0%) was obtained as a white powder from 2-dodecanoyltetradecanoic acid (14-12) as a fatty acid (2.4 mmol).

^1^H NMR (400 MHz, CD_3_OD, ppm) δ: 0.87 (m, 12H), 1.22-1.68 (m, 115H), 1.73 (m, 2H), 1.90 (m, 2H), 2.21-2.45 (m, 8H), 2.89 (m, 2H), 4.08 (m, 6H).

^13^C NMR (125 MHz, CDCl_3_) δ: 24.20, 22.77, 23.55, 26.09, 27.54, 28.81, 29.44, 29.60, 29.65, 29.69, 30.01, 32.00, 32.58, 39.27, 45.90, 46.19, 55.03, 64.11, 64.35, 74.17, 175.12, 176.76.

UPLC/CAD: RT = 29.98 min. MS (ESI): m/z calcd for C_76_H_148_NO_7_ (M+H)^+^, 1187.13; found, 1187.18.

*N-oxidation of ionizable lipids*

*
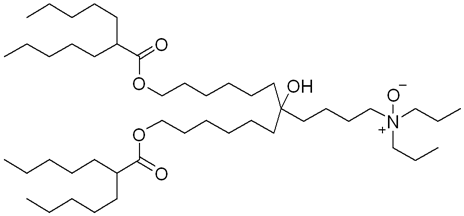
*

*Synthesis of CL4F 7-5 N-oxide (28)*

CL4F 7−5 (0.05 mmol) was dissolved in ethanol (0.5 mL), followed by dropwise addition of hydrogen peroxide (0.2 mmol), and the mixture was stirred at 50 °C for 24 h. The reaction was monitored using ^1^H-NMR spectroscopy. The solvent was removed *in vacuo* to obtain CL4F 7-5 *N*-oxide (28) (39 mg, 101.6%) as a colorless oil.

^1^H NMR (400 MHz, CD_3_OD, ppm) δ: 0.88 (t, 12H), 0.95 (t, 6H), 1.25-1.78 (m, 63H), 2.32 (m, 2H), 3.11 (m, 6H), 4.06 (t, 4H).

UPLC/CAD: RT = 20.91 min. MS (ESI): m/z calculated for C_47_H_94_NO_6_ (M+H)^+^, 768.71; found, 768.61.


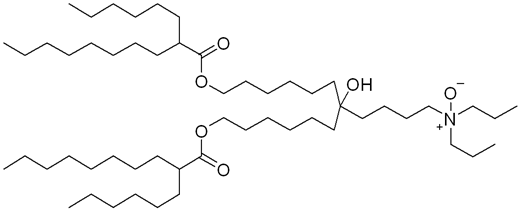


*Synthesis of CL4F 10-6 N-oxide (29)*

Using a procedure analogous to that described for the synthesis of CL4F 7-5 *N*-oxide (5), CL4F 10-6 *N*-oxide (29) (170 mg, 128.7%) was obtained as a colorless oil from CL4F 10-6 as a lipid with a tertiary amine (0.15 mmol).

^1^H NMR (400 MHz, CD_3_OD, ppm) δ: 0.88 (t, 12H), 0.96 (t, 6H), 1.27-1.78 (m, 79H), 2.32 (m, 2H), 3.11 (m, 6H), 4.06 (t, 4H).

UPLC/CAD: RT = 25.62 min. MS (ESI): m/z calcd for C_55_H_110_NO_6_ (M+H)^+^, 880.83; found, 880.83.

*
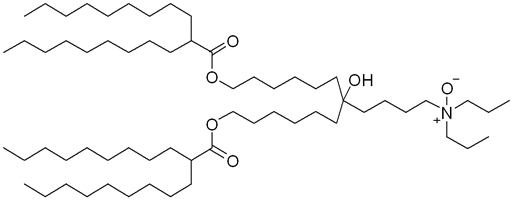
*

*Synthesis of CL4F 11-9 N-oxide (30)*

Using a procedure analogous to that described for the synthesis of CL4F 7-5 *N*-oxide (5), CL4F 11-9 *N*-oxide (30) (49 mg, 98.8%) was obtained as a colorless oil from CL4F 11-9 as a lipid with a tertiary amine (0.05 mmol).

^1^H NMR (400 MHz, CD_3_OD, ppm) δ: 0.88 (t, 12H), 0.95 (t, 6H), 1.27-1.79 (m, 95H), 2.32 (m, 2H), 3.11 (m, 6H), 4.06 (t, 4H).

UPLC/CAD: RT = 36.50 min. MS (ESI): m/z calcd for C_63_H_126_NO_6_ (M+H)^+^, 992.96; found, 992.97.


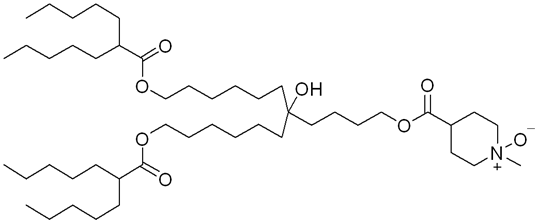


*Synthesis of CL15F 7-5 N-oxide (31)*

Using a procedure analogous to that described for the synthesis of CL4F 7-5 *N*-oxide (5), CL15F 7-5 *N*-oxide (31) (37 mg, 91.4%) was obtained as a colorless oil from CL15F 7-5 as a lipid with a tertiary amine (0.05 mmol).

^1^H NMR (400 MHz, CD_3_OD, ppm) δ: 0.88 (t, 12H), 1.22-1.67 (m, 61H), 1.85-2.02 (m, 2H), 2.33 (m, 4H), 2.50 (m, 1H), 3.16 (s, 3H), 3.43 (m, 2H), 4.05 (t, 4H), 4.10 (t, 2H).

UPLC/CAD: RT = 20.80 min. MS (ESI): m/z calcd for C_48_H_92_NO_8_ (M+H)^+^, 810.68; found, 810.68.


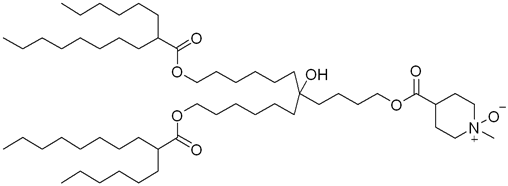


*Synthesis of CL15F 10-6 N-oxide (32)*

Using a procedure analogous to that described for the synthesis of CL4F 7-5 *N*-oxide (5), CL15F 10-6 *N*-oxide (32) (167 mg, 120.3%) was obtained as a colorless oil from CL15F 10-6 as a lipid with a tertiary amine (0.15 mmol).

^1^H NMR (400 MHz, CD_3_OD, ppm) δ: 0.88 (t, 12H), 1.21-1.65 (m, 77H), 1.86-2.05 (m, 2H), 2.32 (m, 4H), 2.51 (m, 1H), 3.19 (s, 3H), 3.43 (m, 2H), 4.05 (t, 4H), 4.11 (t, 2H).

UPLC/CAD: RT = 25.92 min. MS (ESI): m/z calcd for C_56_H_108_NO_8_ (M+H)^+^, 922.81; found, 922.78.

*
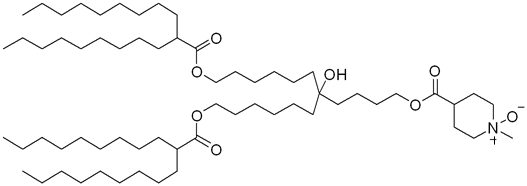
*

*Synthesis of CL15F 11-9 N-oxide (33)*

Using a procedure analogous to that described for the synthesis of CL4F 7-5 *N*-oxide (5), CL15F 11-9 *N*-oxide (33) (48 mg, 92.8%) was obtained as a colorless oil from CL15F 11-9 as a lipid with a tertiary amine (0.05 mmol).

^1^H NMR (400 MHz, CD_3_OD, ppm) δ: 0.88 (t, 12H), 1.21-1.64 (m, 93H), 1.86-2.07 (t, 2H), 2.32 (m, 4H), 2.50 (m, 1H), 3.16 (s, 3H), 3.43 (m, 2H), 4.05 (t, 4H), 4.10 (t, 2H).

UPLC/CAD: RT = 33.74 min. MS (ESI): m/z calcd for C_64_H_124_NO_8_ (M+H)^+^, 1034.93; found, 1034.96.

*Synthesis of CL6F 14-12*

*
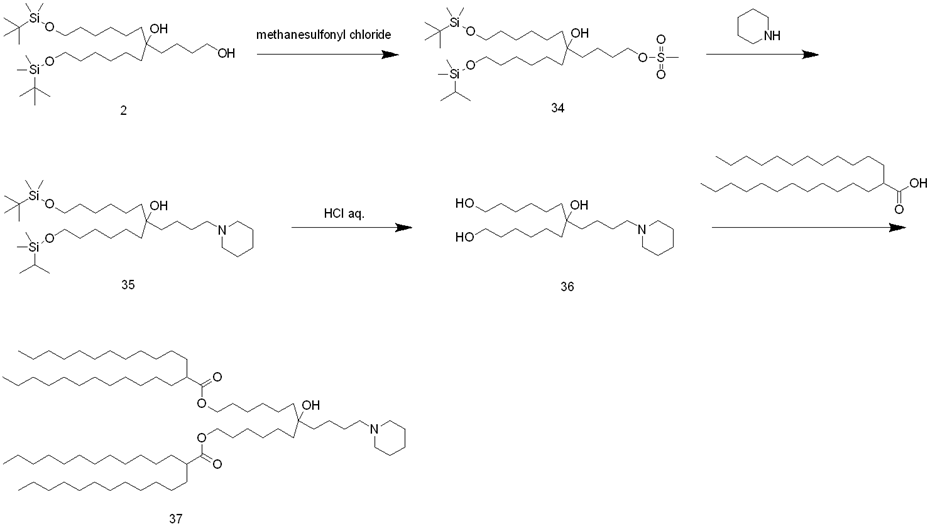
*

*Synthesis of 11-((tert-butyldimethylsilyl)oxy)-5-hydroxy-5-(6-((isopropyldimethylsilyl)oxy)hexyl)undecyl methanesulfonate (34)*

Compound 2 (1.6 g, 3.0 mmol) was dissolved in 9 mL of anhydrous DCM and the mixture was cooled on ice. DMAP (47.7 mg, 0.39 mmol), TEA (0.75 mL, 5.4 mmol), and methanesulfonyl chloride (460 mg, 4.02 mmol) were successively added to the mixture on ice. The reaction mixture was stirred at room temperature overnight. Evaporation of the solvent resulted in a yellow oily residue. The residue was suspended with hexane/ethyl acetate and the suspension was filtered through a pad of celite and Wakogel C-100. The filtrate obtained was washed with saturated citric acid aqueous solution, and then dried over anhydrous Na_2_SO_4_. Evaporation of the solvent gave crude 34 as a pale-yellow oil.

*Synthesis of 2,2,3,3,19,19,20-heptamethyl-11-(4-(piperidin-1-yl)butyl)-4,18-dioxa-3,19-disilahenicosan-11-ol (35)*

Anhydrous THF (2.15 mL) and piperidine (1.5 mL, 15.18 mmol) were added to compound 34 (1.535 g, 2.53 mmol) and the reaction mixture was stirred at 60 °C overnight. Removal of the solvent *in vacuo* gave crude 35 as a pale-yellow oil.

*Synthesis of 7-(4-(piperidin-1-yl)butyl)tridecane-1,7,13-triol (36)*

Compound 35 (1.52 g, 2.53 mmol) was dissolved in ethanol (125 mL) and the solution was cooled on ice. 12 N HCl solution (1.27 mL, 15.2 mmol) was added to the solution and the reaction mixture was stirred at room temperature for 1 h. After evaporation of the reaction mixture, the residue was suspended with water and washed with dichloromethane. After the obtained aqueous phase was diluted with dichloromethane, compound 11 was extracted to the organic phase by basification of the aqueous layer with a 5N NaOH solution. The organic phase was washed with brine and dried over Na_2_SO_4_. After removal of the solvent *in vacuo*, the residue was purified by flash chromatography (SiO_2_, DCM/MeOH). This gave 445 mg (47.4%) of compound 36 as a colorless oil.

^1^H NMR (400 MHz, CDCl_3_, ppm) δ: 1.23-1.68 (m, 35H), 2.35 (m, 3H), 2.43 (m, 3H), 3.60 (m, 4H).

*Synthesis of CL6F 14-12 (37)*

2-Dodecanoyltetradecanoic acid (14-12) as a fatty acid (190.4 mg, 0.48 mmol) was added to 7-(4-(piperidin-1-yl)butyl)tridecane-1,7,13-triol (36) (74.3 mg, 0.2 mmol) in anhydrous DCM (1 mL). DMAP (2.44 mg, 0.02 mmol) and EDCI-HCl (115 mg, 0.6 mmol) were added to the mixture and the reaction mixture was stirred at 25 °C overnight. After evaporation of the solvent, the residue was suspended in ethyl acetate and washed with 0.5N NaOH solution and brine. The organic phase was dried over Na_2_SO_4_. Evaporation of the solvent produced crude as a yellow oily residue. The residue was purified using flash chromatography [ODS, H_2_O (0.1% trifluoroacetic acid)/acetonitrile:isopropanol = 50:50 (0.1% TFA)] and (SiO_2_, DCM/MeOH). This gave 56 mg of CL6F 14-12 (37) as a colorless oil.

^1^H NMR (300 MHz, CD_3_OD, ppm) δ: 0.88 (t, 12H), 1.20-2.90 (m, 122H), 2.32 (m, 2H), 3.06 (m, 5H), 4.06 (t, 4H).

UPLC/CAD: RT = 25.89 in. MS (ESI): m/z calcd for C_74_H_146_NO_5_ (M+H)^+^, 1129.12; found, 1129.09.

*Synthesis of CL16F 14-12*

*
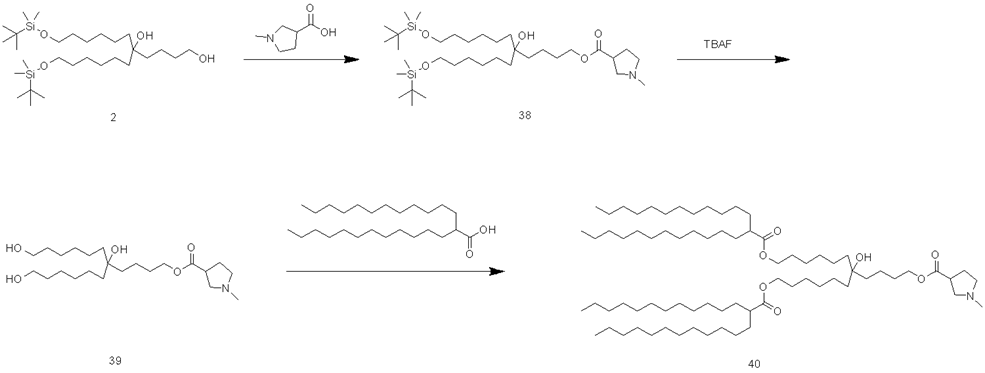
*

*Synthesis of 11-((tert-butyldimethylsilyl)oxy)-5-(6-((tert-butyldimethylsilyl)oxy)hexyl)-5-hydroxyundecyl 1-methylpyrrolidine-3-carboxylate (38)*

Compound 2 (1.09 g, 2.05 mmol) was dissolved in anhydrous DCM (10 mL) and 1-methylpyrrolidine-3-carboxylic acid (291 mg, 2.255 mmol), DMAP (24.4 mg, 0.2 mmol) and EDCI (472 mg, 2.46 mmol) were added to this mixture. The reaction mixture was stirred at ambient temperature for 2 days. After evaporation of the reaction mixture, the residue was suspended with ethyl acetate (200 mL) and washed with NaOH solution (0.5 M, 200 mL) and brine (100 mL). The organic phase was dried over Na_2_SO_4_. Evaporation of the solvent gave 1.36 g compound 38 as a pale-yellow oily residue.

*Synthesis of 5,11-dihydroxy-5-(6-hydroxyhexyl)undecyl 1-methylpyrrolidine-3-carboxylate* *(39)*

Acetic acid (418 µL, 7.32 mmol) and tetrabutylammonium fluoride (TBAF) in THF (1.0 M, 5.87 mL) was successively added to compound 38 (1.36 g, 2.11 mmol) at 0 °C. The reaction mixture was stirred at room temperature overnight. After evaporation of the reaction mixture, the residue was suspended with saturated citric acid aqueous solution and washed with dichloromethane. After the aqueous phase was diluted with ethyl acetate, compound 14 was extracted to the organic phase by basification of the aqueous layer with a 5N NaOH solution. The organic phase was washed with brine and dried over Na_2_SO_4_. Evaporation of the solvent gave 660 mg of crude as a yellow oily residue. The residue was purified by flash chromatography (SiO_2_, dichloromethane/MeOH). This gave 285 mg (32.5%) of compound 39 as a pale-yellow oil.

^1^H NMR (400 MHz, CDCl_3_, ppm) δ: 1.15-1.85 (m, 29H), 2.15 (m, 2H), 2.43 (s, 3H), 2.56 (m, 1H), 2.72-2.85 (m, 2H), 2.95-3.15 (m, 2H), 3.62 (m, 4H), 4.10 (m, 2H).

*Synthesis of CL16F 14-12 (40)*

2-Dodecanoyltetradecanoic acid (14-12) as a fatty acid (190.4 mg, 0.48 mmol) was added to 5,11-dihydroxy-5-(6-hydroxyhexyl)undecyl 1-methylpyrrolidine-3-carboxylate (39) (83.1 mg, 0.2 mmol) in anhydrous DCM (1 mL). DMAP (2.44 mg, 0.02 mmol) and EDCI-HCl (115 mg, 0.6 mmol) were added to the mixture and the reaction mixture was stirred at 25 °C overnight. After evaporation of the solvent, the residue was suspended in ethyl acetate and washed with 0.5 N NaOH solution and brine. The organic phase was dried over Na_2_SO_4_. Evaporation of the solvent produced crude as a yellow oily residue. The residue was purified using flash chromatography [ODS, H_2_O (0.1% trifluoroacetic acid)/acetonitrile:isopropanol = 50:50 (0.1% TFA)] and (SiO_2_, DCM/MeOH). This gave 106 mg of CL16F 14-12 (40) as a colorless oil.

^1^H NMR (400 MHz, CD_3_OD, ppm) δ: 0.88 (t, 12H), 1.23-1.62 (m, 116H), 2.14 (m, 2H), 2.32 (m, 2H), 2.50 (s, 3H), 2.82 (m, 2H), 3.01 (m, 2H), 4.07 (m, 6H).

UPLC/CAD: RT = 25.75 in. MS (ESI): m/z calcd for C_75_H_146_NO_7_ (M+H)^+^, 1173.11; found, 1173.12.

*Synthesis of CL17F 14-12*


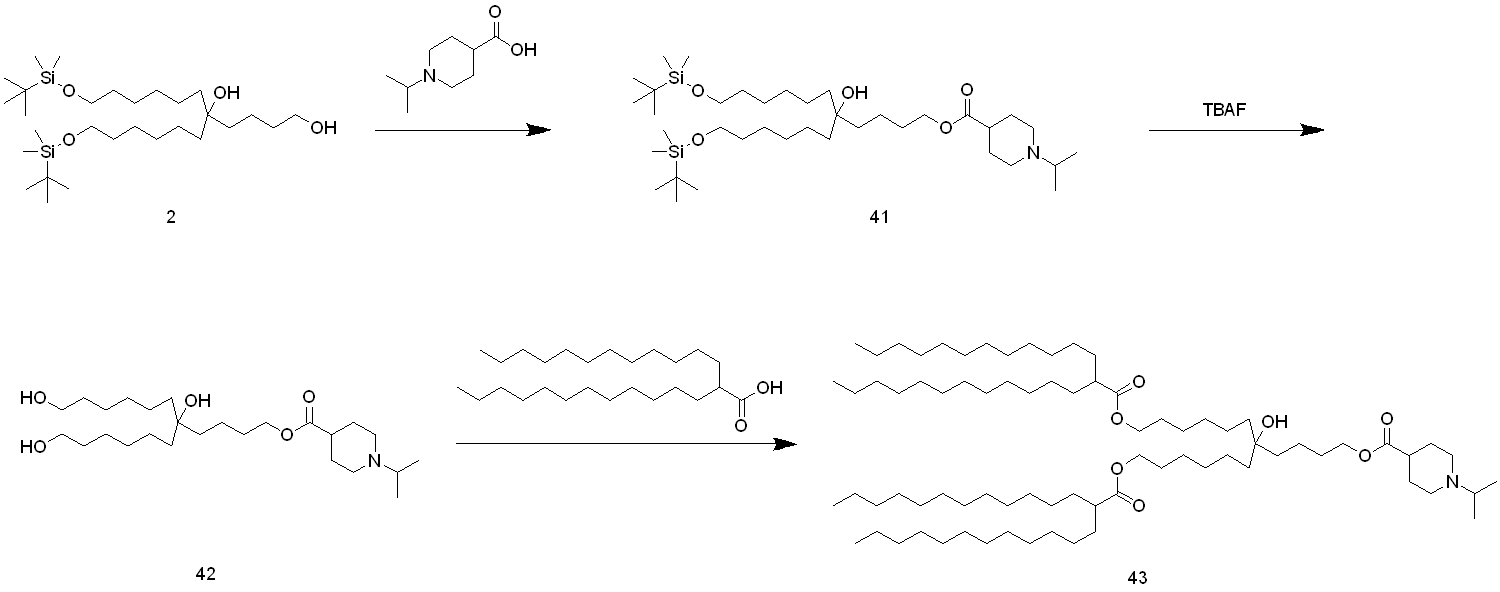


*Synthesis of 11-((tert-butyldimethylsilyl)oxy)-5-(6-((tert-butyldimethylsilyl)oxy)hexyl)-5-hydroxyundecyl 1-isopropylpiperidine-4-carboxylate (41)*

Compound 2 (1.20 g, 2.25 mmol) was dissolved in anhydrous DCM (10 mL) and 1-(propan-2-yl)piperidine-4-carboxylic acid HCl (548 mg, 2.64 mmol), DMAP (29.3 mg, 0.24 mmol), and EDCI (552 mg, 2.88 mmol) were added to this mixture. The reaction mixture was stirred at ambient temperature for 2 days. After evaporation of the reaction mixture, the residue was suspended with ethyl acetate (200 mL) and washed with NaOH solution (0.5 M, 200 mL) and brine (100 mL). The organic phase was dried over Na_2_SO_4_. Evaporation of the solvent gave 1.67 g compound 41 as a pale-yellow oily residue.

*Synthesis of 5,11-dihydroxy-5-(6-hydroxyhexyl)undecyl 1-isopropylpiperidine-4-carboxylate (42)*

Acetic acid (482 µL, 8.43 mmol) and tetrabutylammonium fluoride (TBAF) in THF (1.0 M, 6.75 mL) were successively added to compound 41 (1.67 g, 2.43 mmol) at 0 °C. The reaction mixture was stirred at room temperature overnight. After evaporation of the reaction mixture, the residue was suspended with saturated citric acid aqueous solution and washed with dichloromethane. After the aqueous phase was diluted with ethyl acetate, compound 42 was extracted to the organic phase by basification of the aqueous layer with a 5 N NaOH solution. The organic phase was washed with brine and dried over Na_2_SO_4_. After the evaporation of the solvent, the residue was purified by flash chromatography (SiO2, dichloromethane/MeOH). This gave 628 mg (56.5%) of compound 42 as a pale-yellow oil.

*Synthesis of CL17F 14-12 (43)*

2-Dodecanoyltetradecanoic acid (14-12) as a fatty acid (285.6 mg, 0.72 mmol) was added to 5,11-dihydroxy-5-(6-hydroxyhexyl)undecyl 1-isopropylpiperidine-4-carboxylate (42) (137.3 mg, 0.3 mmol) in anhydrous DCM (1.5 mL). DMAP (3.66 mg, 0.03 mmol) and EDCI-HCl (172.5 mg, 0.9 mmol) were added to the mixture and the reaction mixture was stirred at 25 °C overnight. After evaporation of the solvent, the residue was suspended in ethyl acetate and washed with 0.5 N NaOH solution and brine. The organic phase was dried over Na_2_SO_4_. Evaporation of the solvent produced crude as a yellow oily residue. The residue was purified using flash chromatography [ODS, H_2_O (0.1% trifluoroacetic acid)/acetonitrile:isopropanol = 50:50 (0.1% TFA)] and (SiO_2_, DCM/MeOH). This gave 106 mg of CL17F 14-12 (43) as a colorless oil.

^1^H NMR (400 MHz, CD_3_OD, ppm) δ: 0.88 (t, 12H), 1.07 (d, 6H), 1.27-1.80 (m, 117H), 1.90 (m, 2H), 2.32 (m, 5H), 2.72 (m, 1H), 2.89 (m, 2H), 4.07 (m, 6H).

UPLC/CAD: RT = 25.75 in. MS (ESI): m/z calcd for C_78_H_152_NO_7_ (M+H)^+^, 1215.16; found, 1215.19.

NMR images

CL15F 6-2


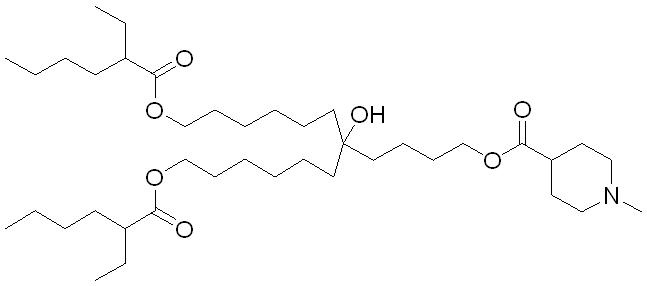


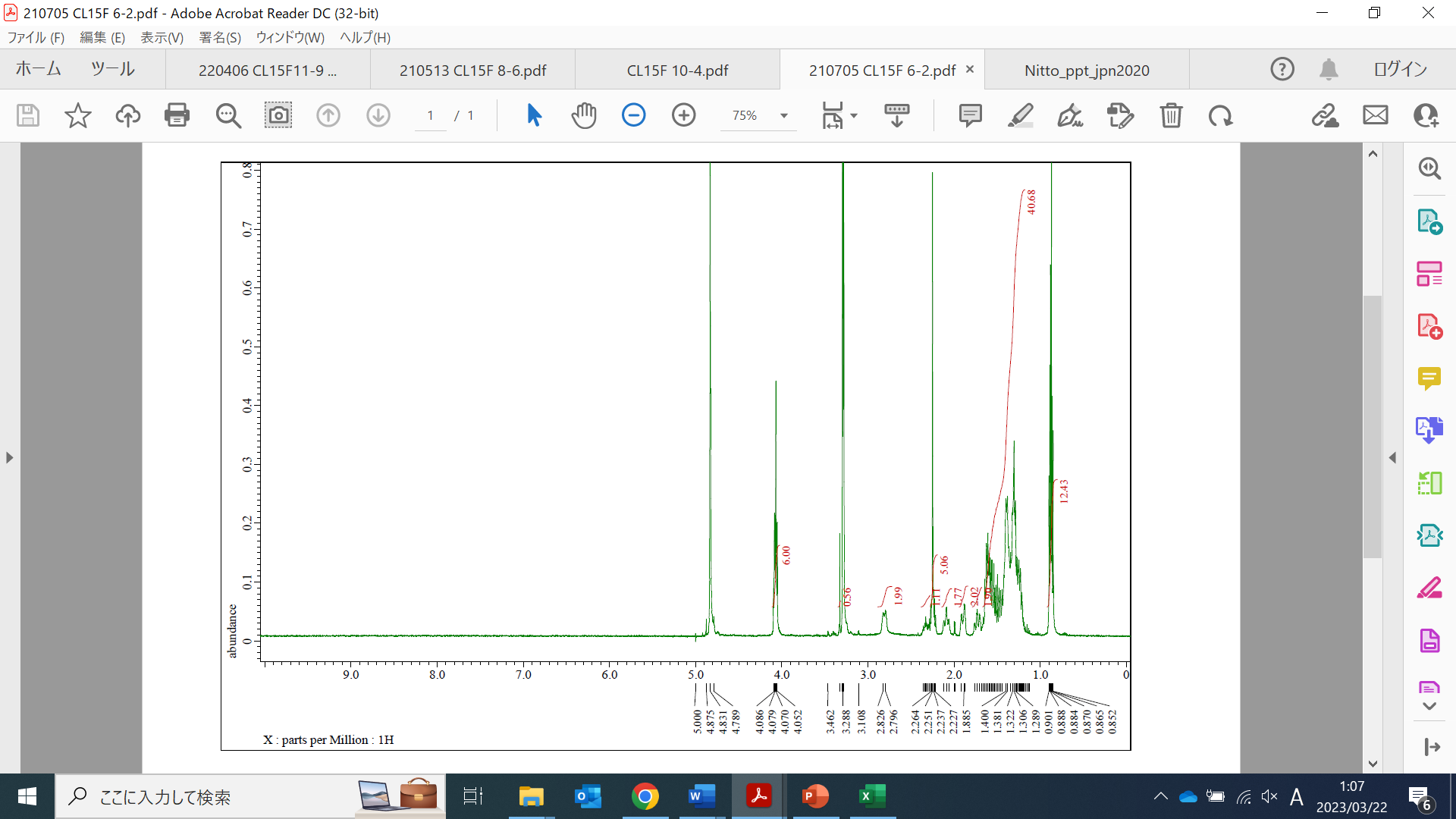


CL15F 6-4


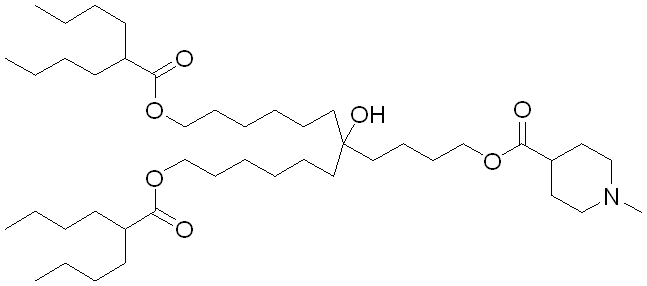


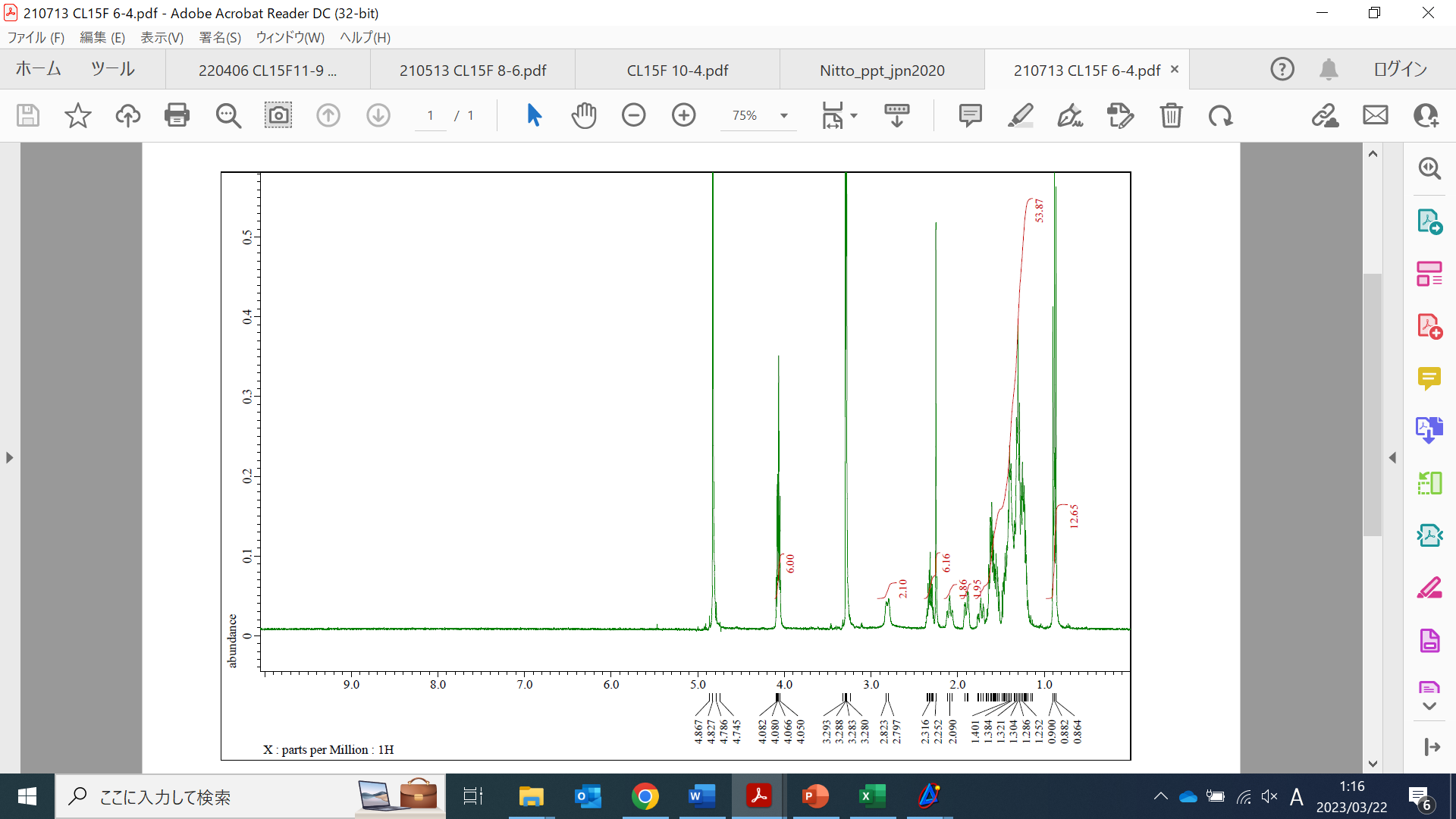


CL15F 7-3


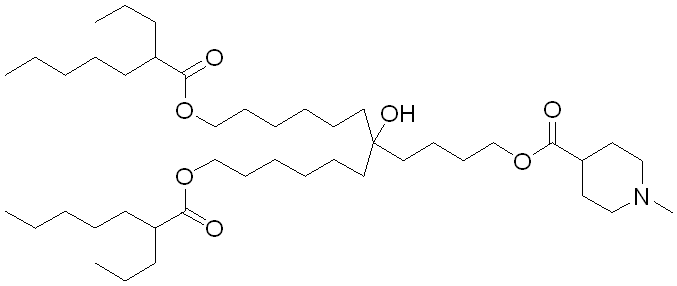


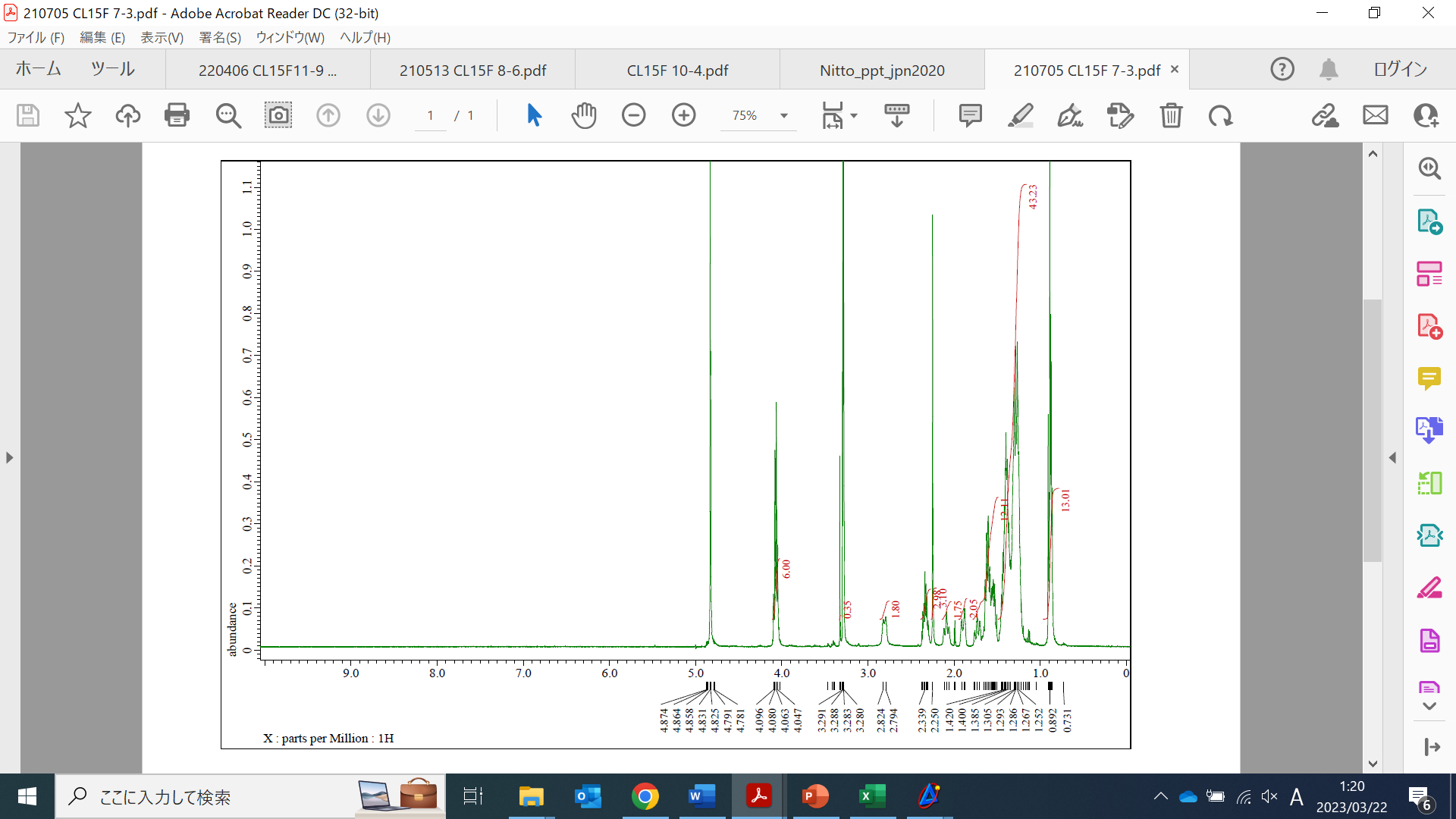


CL15F 7-5


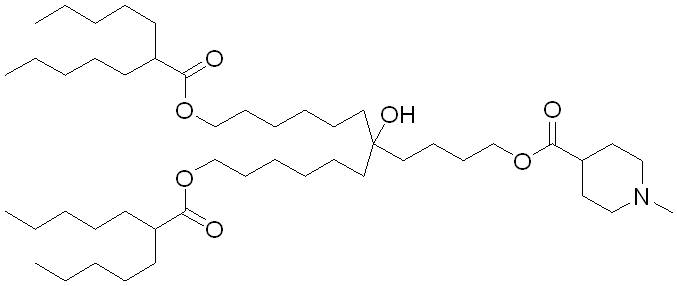


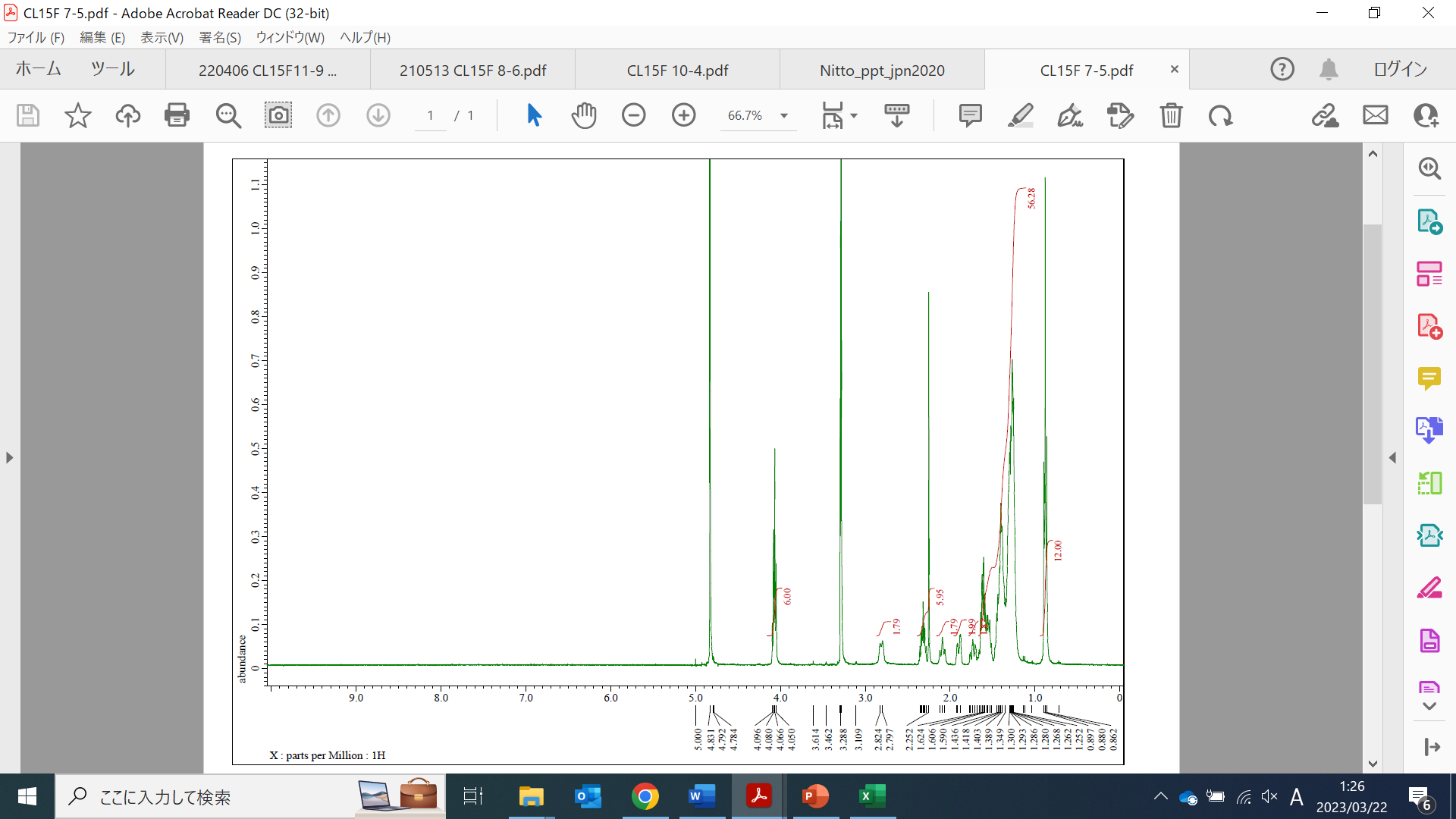


CL15F 9-3


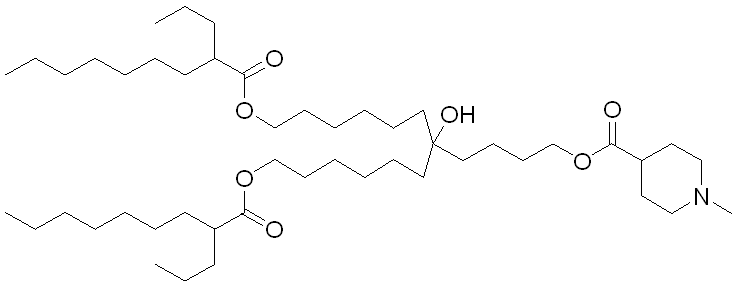


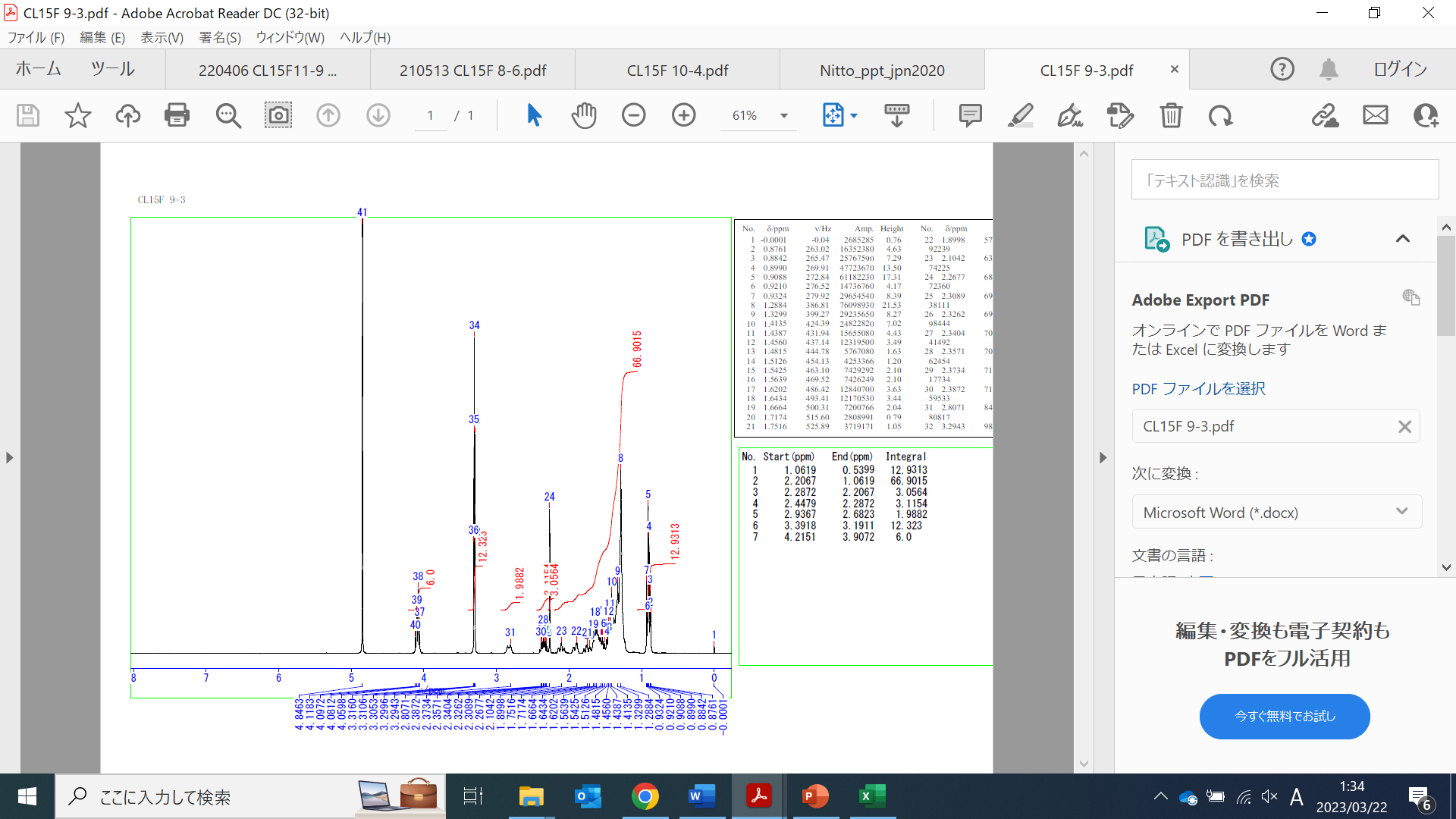


CL15F 8-6


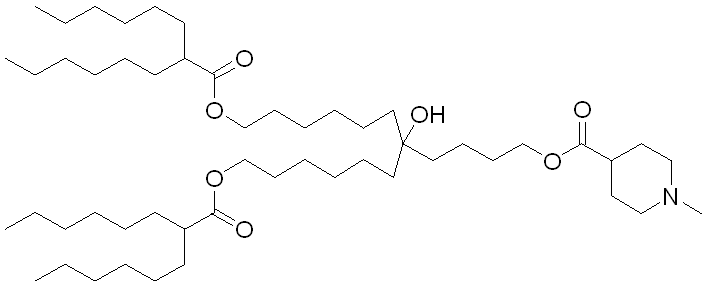


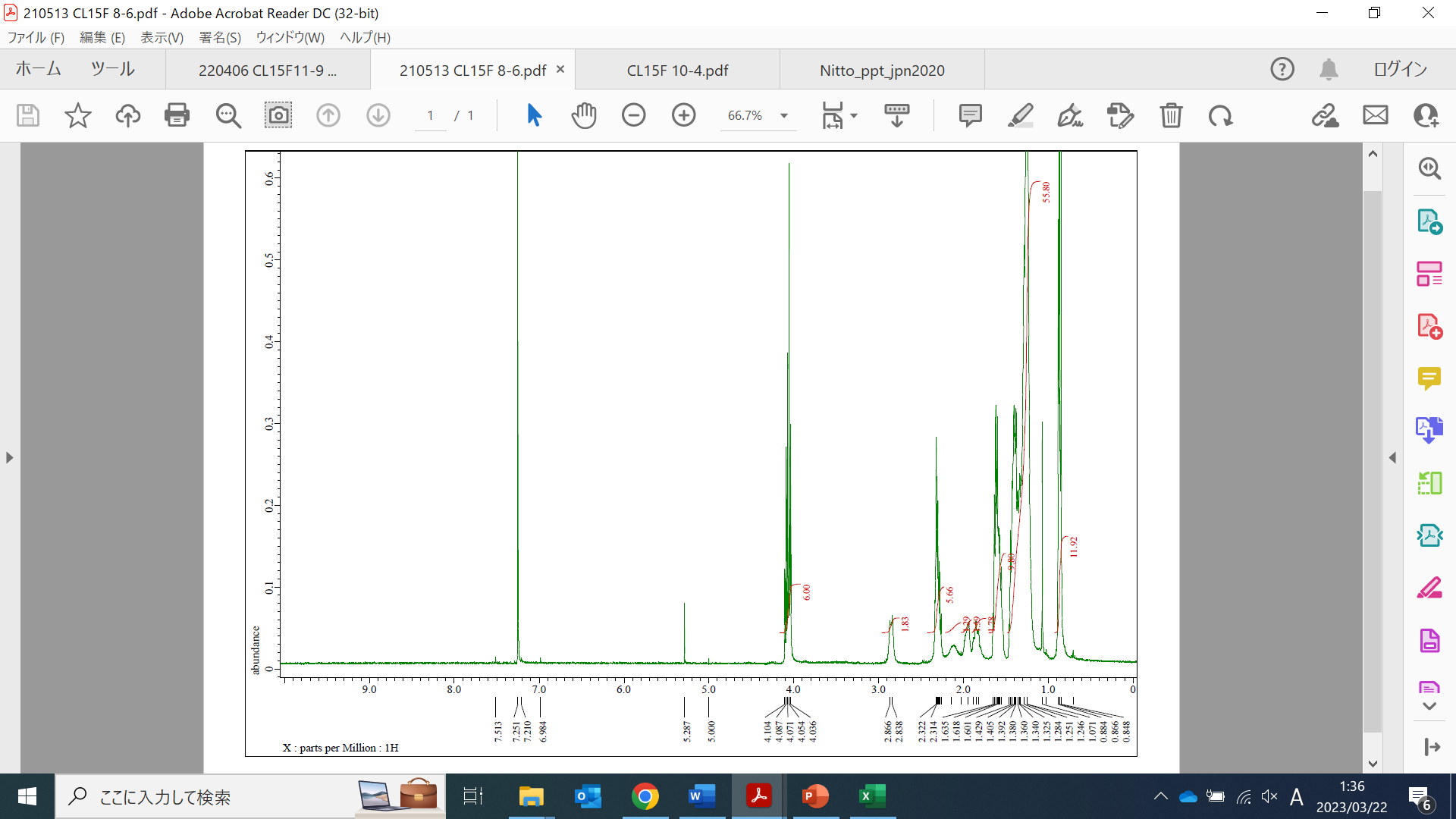


CL15F 9-5


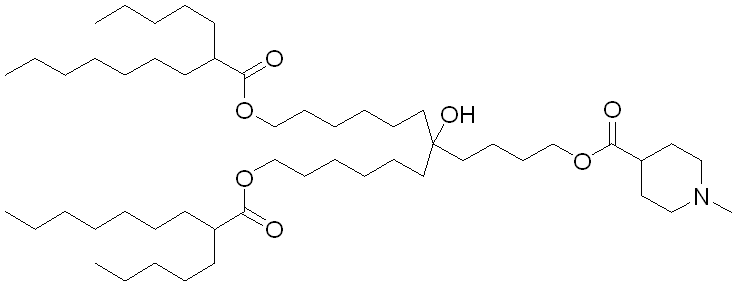


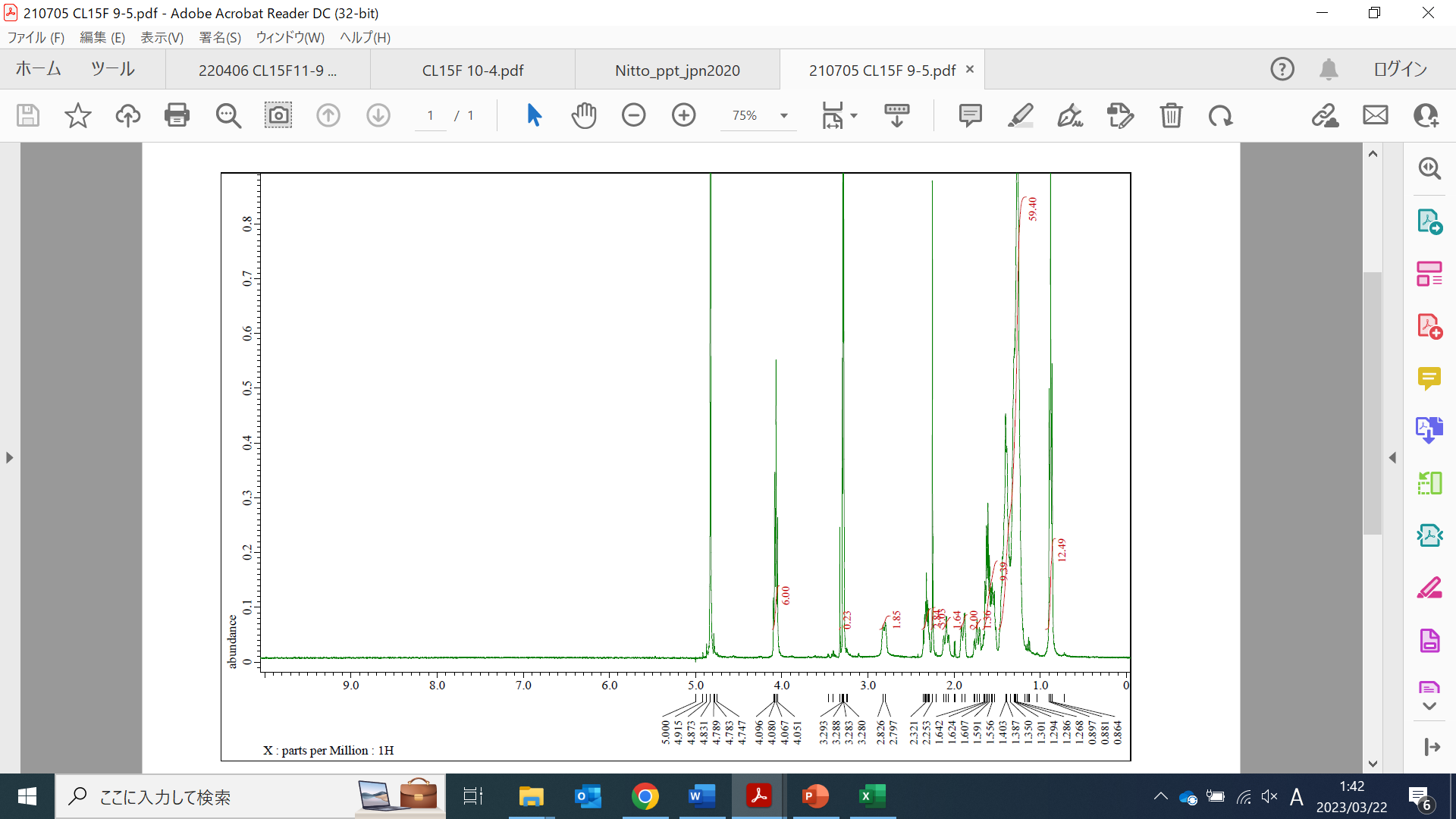


CL15F 10-4


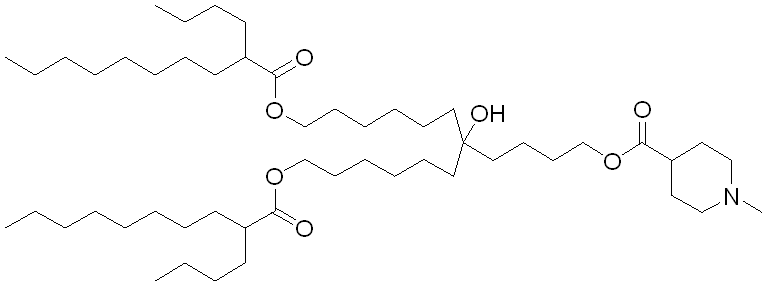


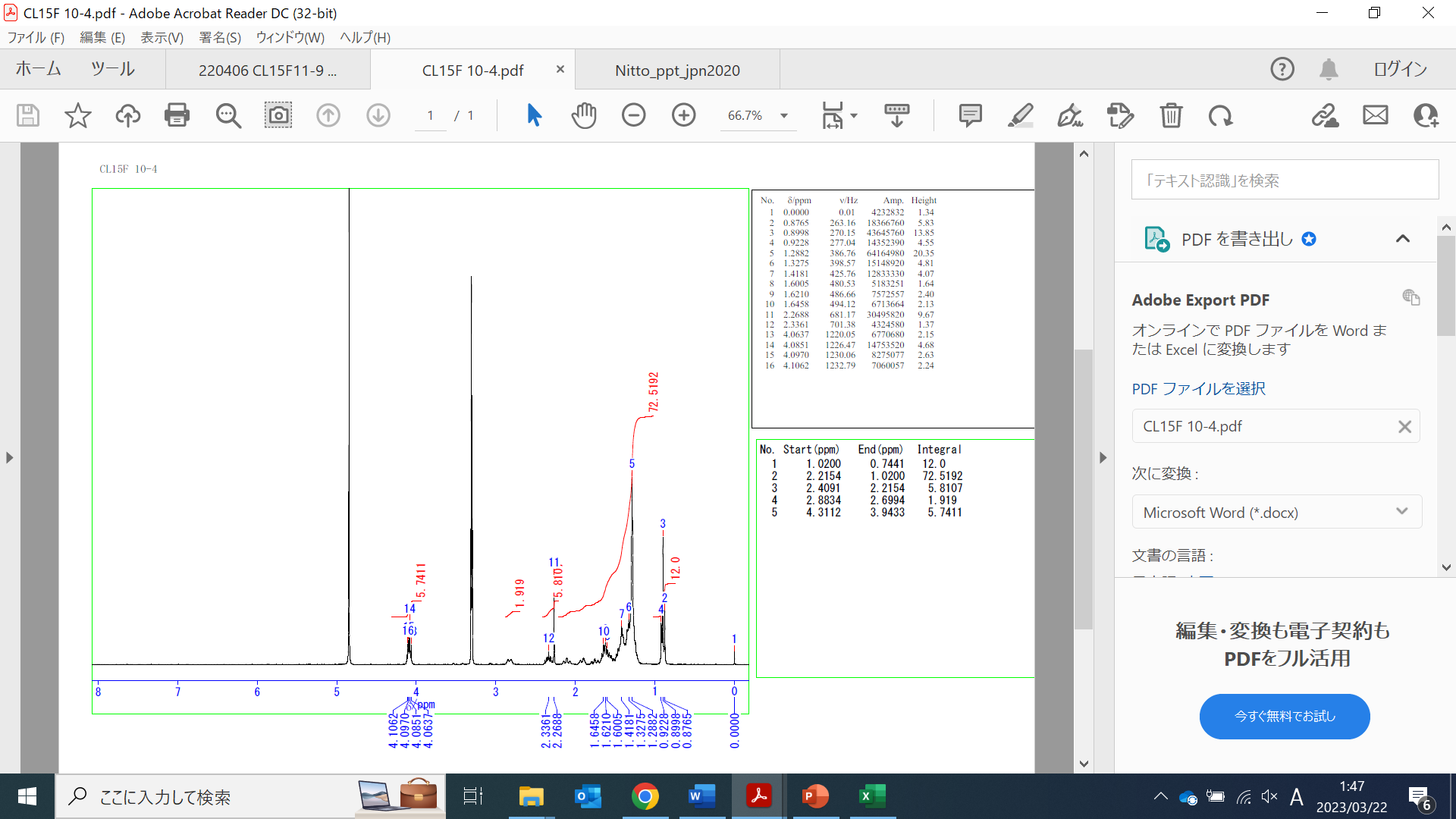


CL15F 10-5


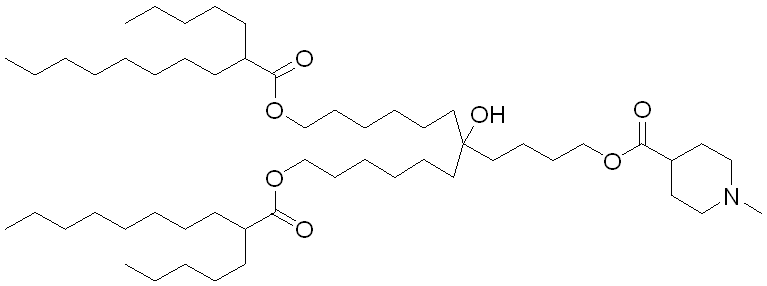


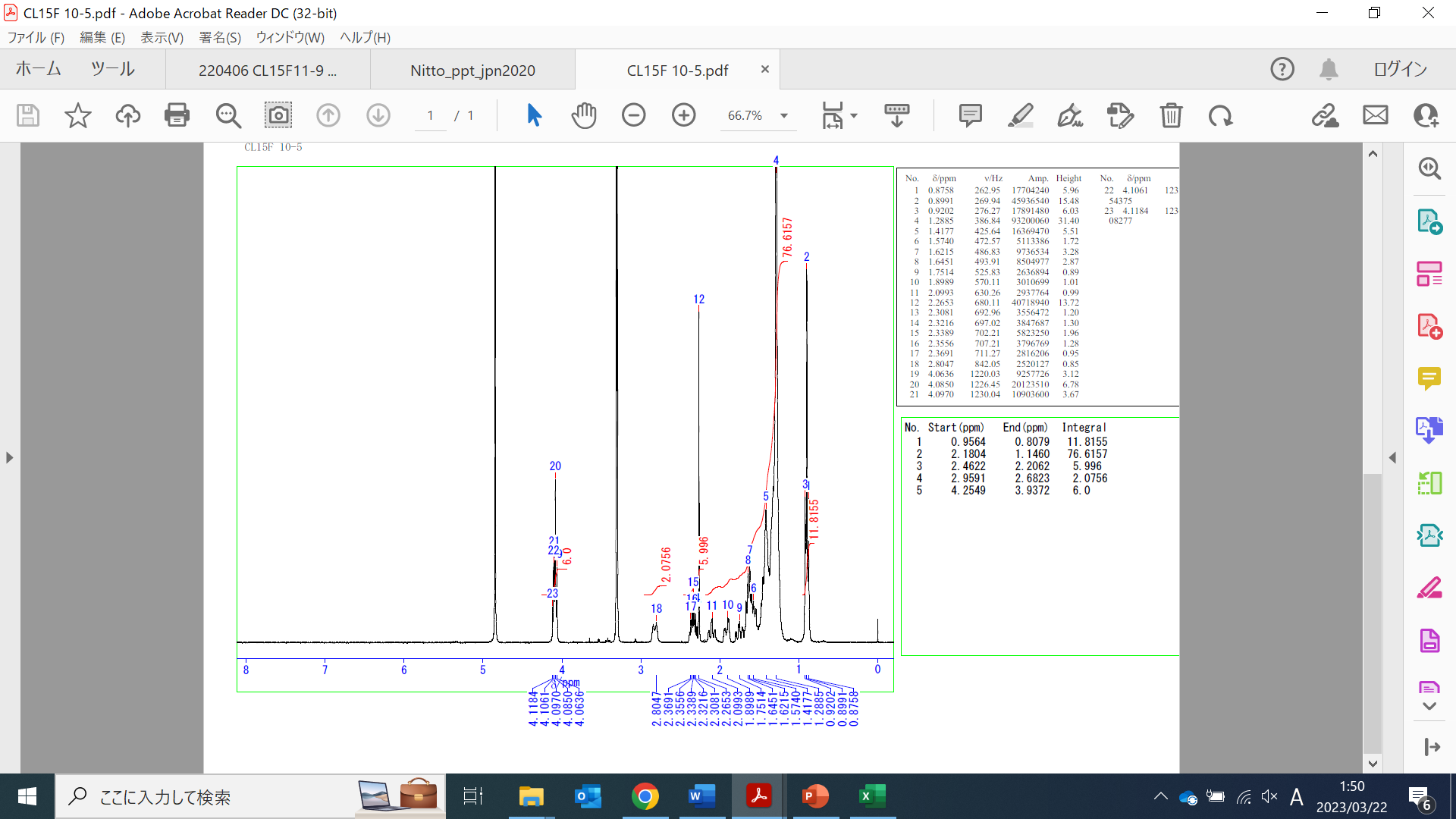


CL15F 9-7


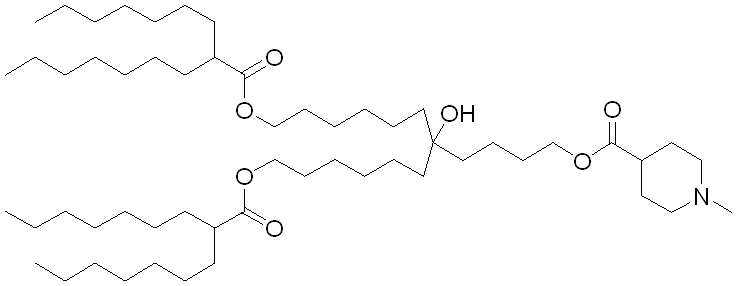


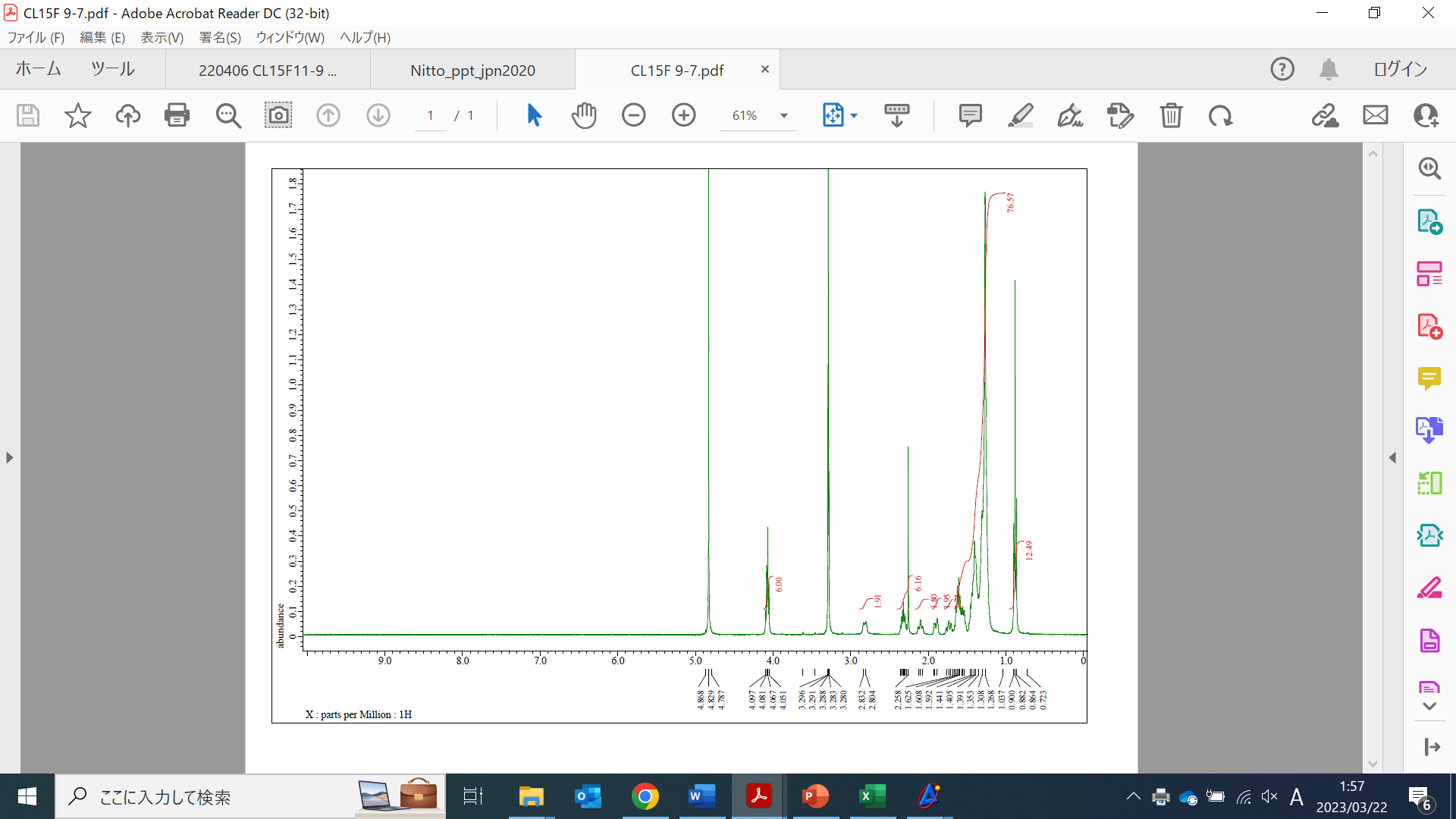


CL15F 11-5


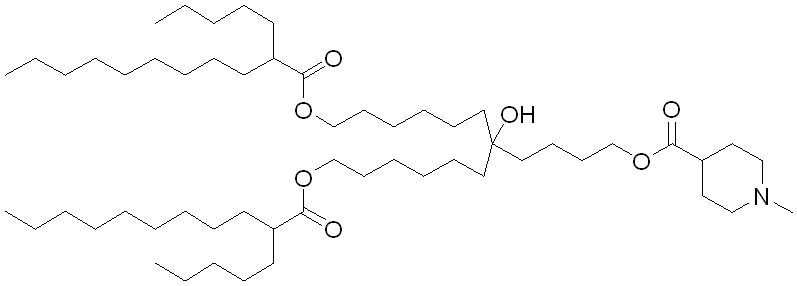


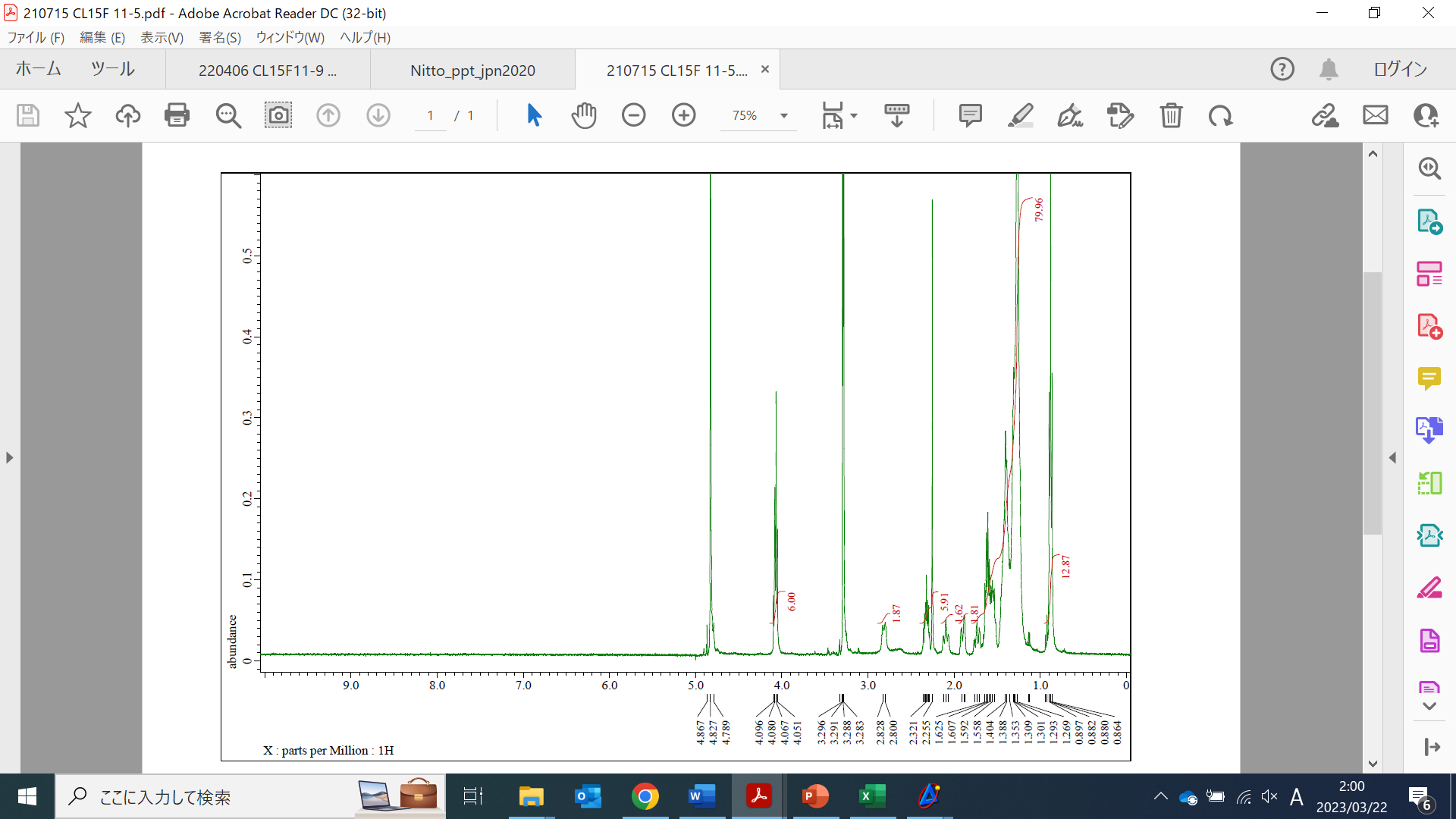


CL15F 12-4


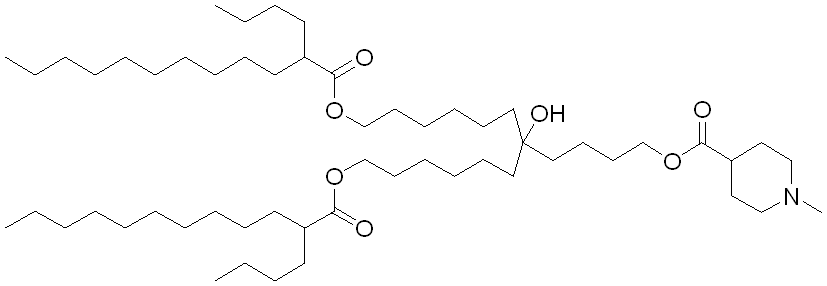


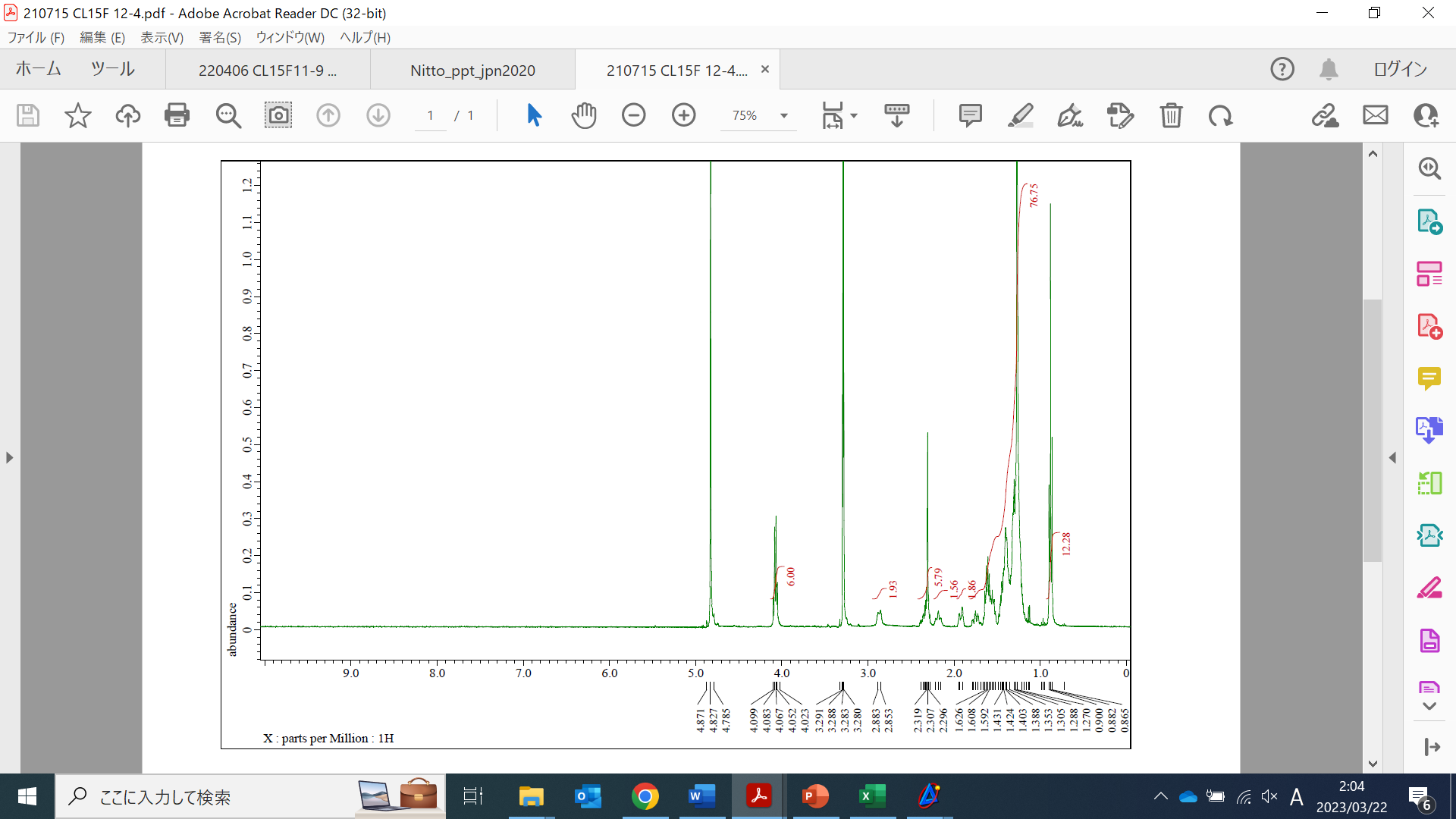


CL15F 16-0


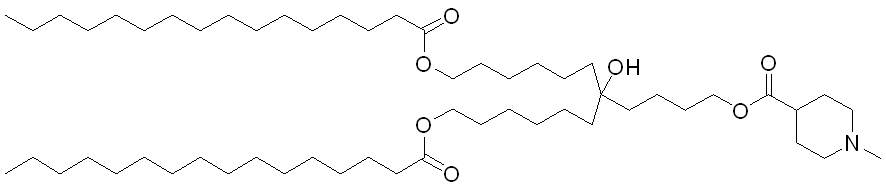


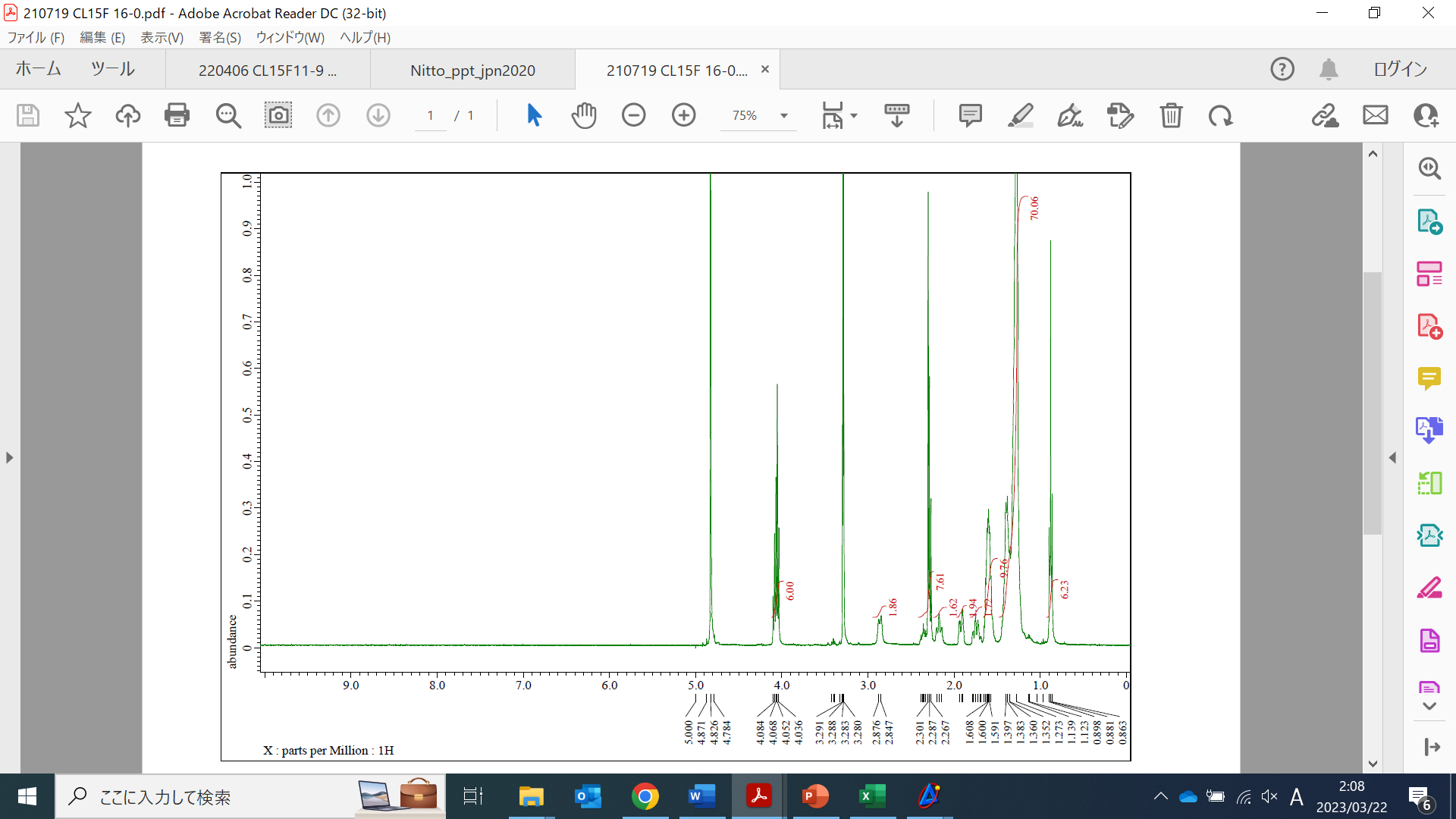


CL15F 13-3


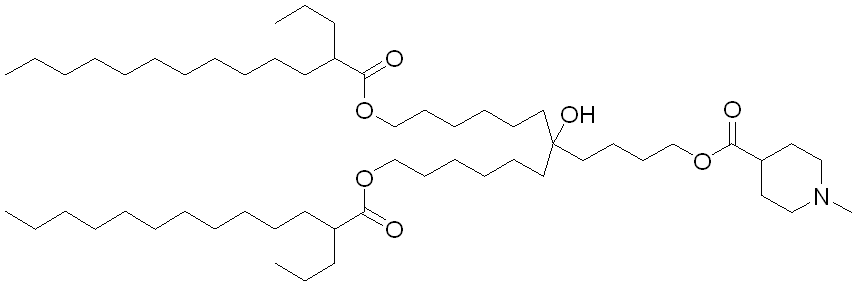


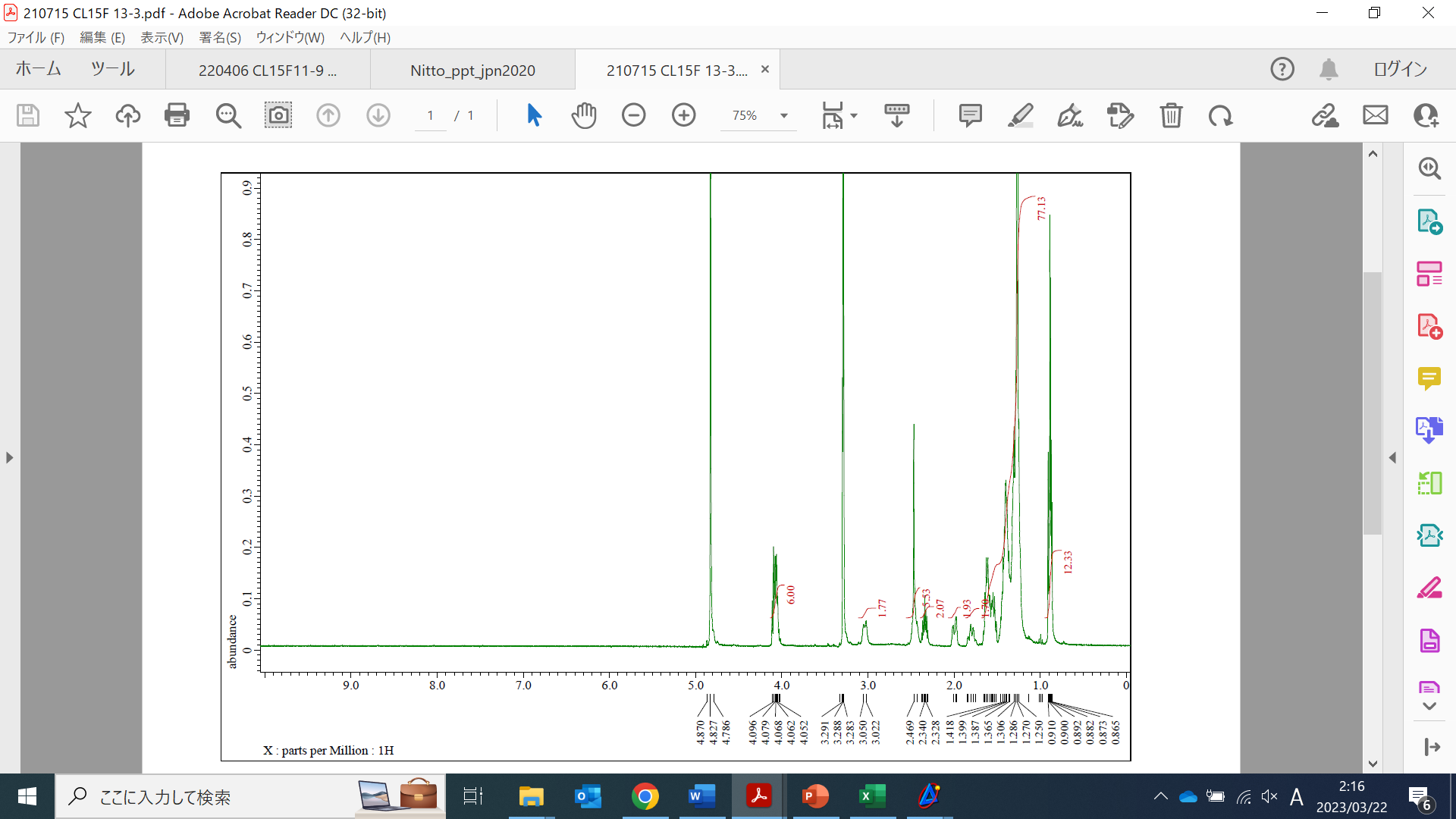


CL15F 11-6


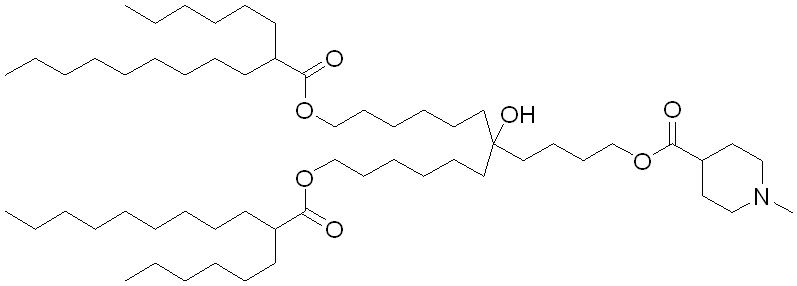


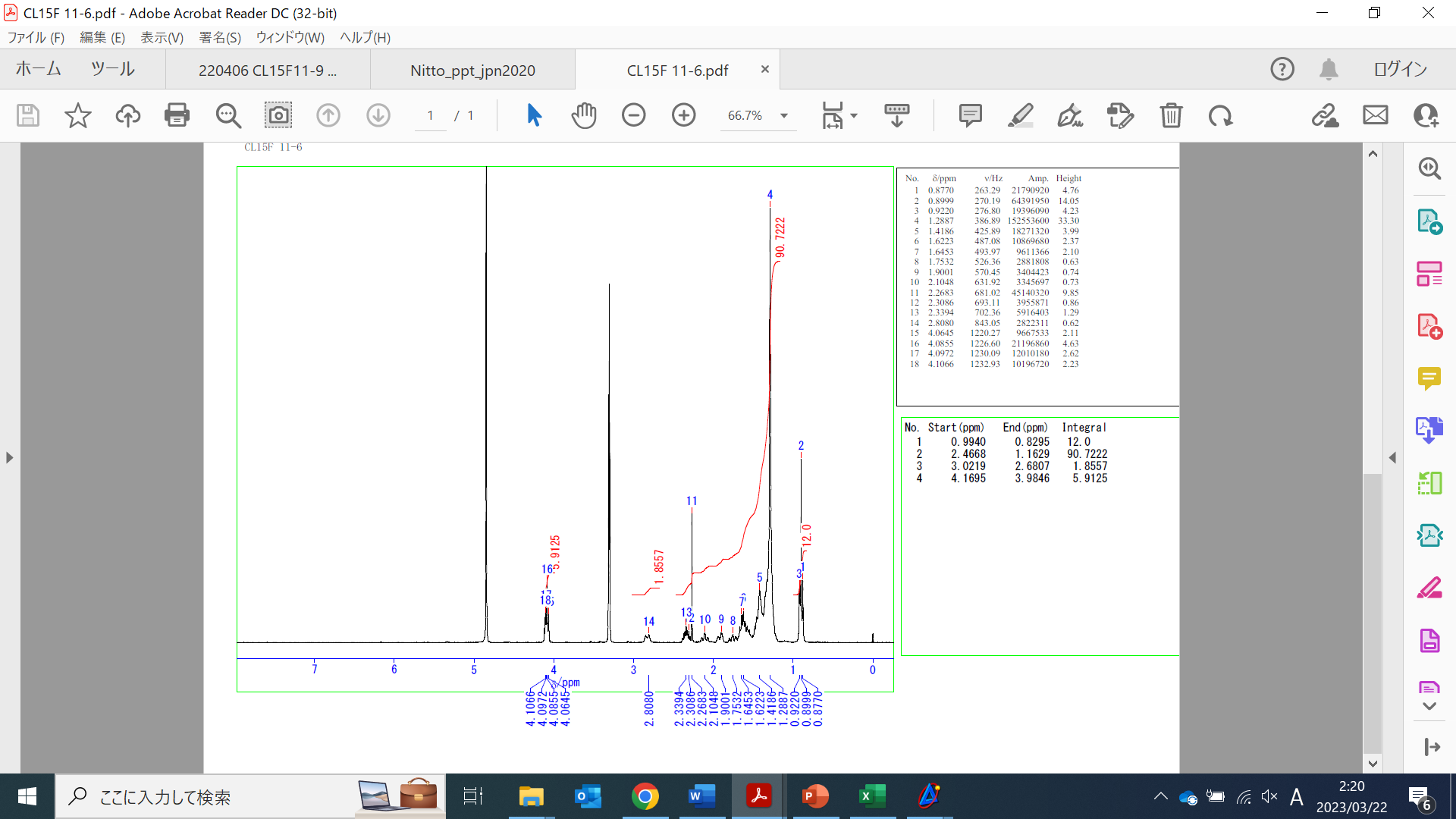


CL15F 16-1


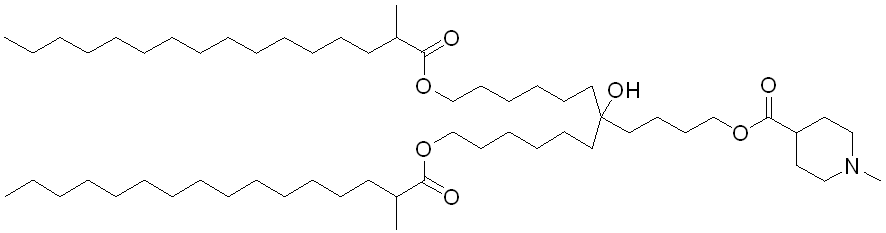


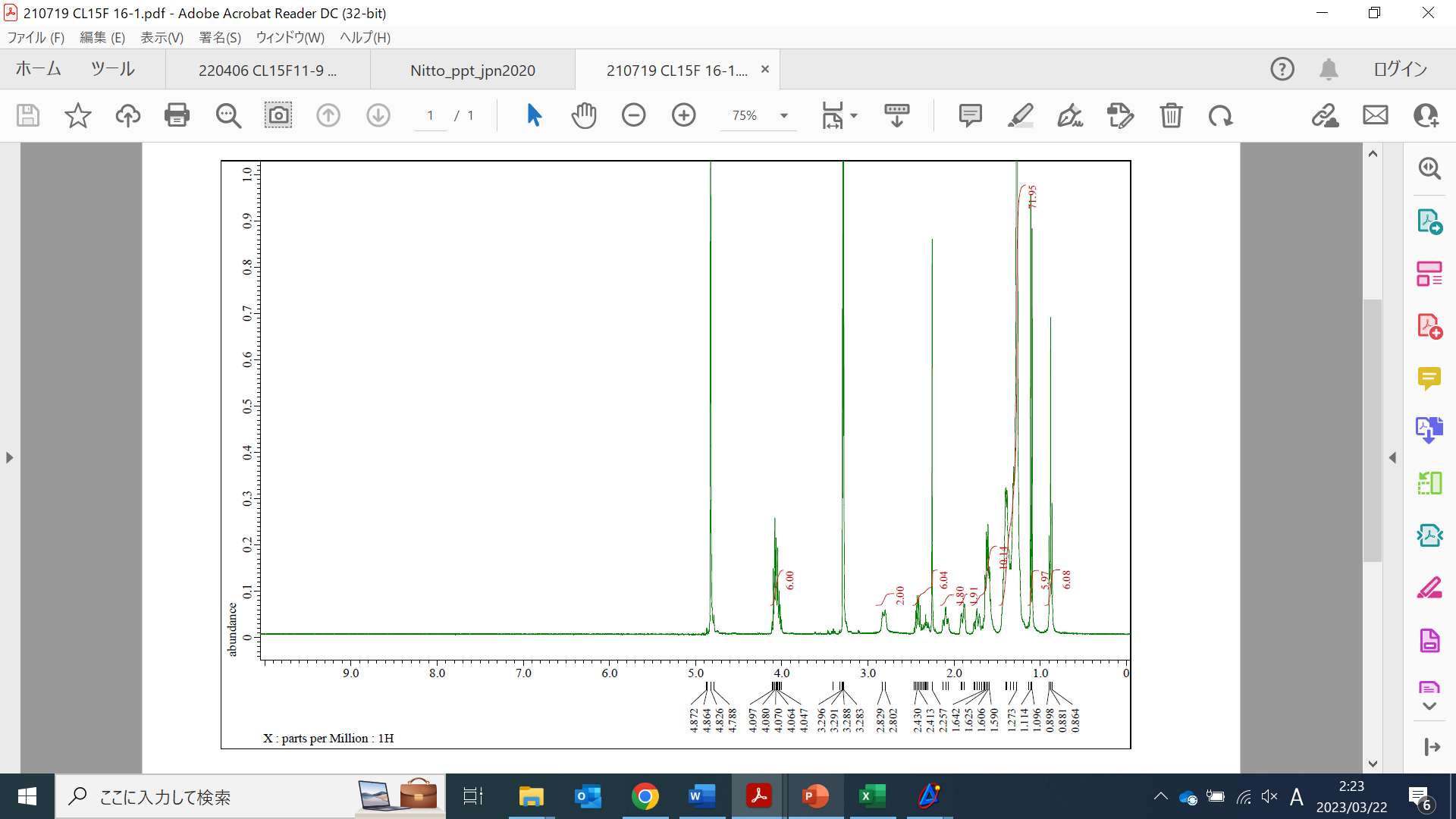


CL15F 10-8


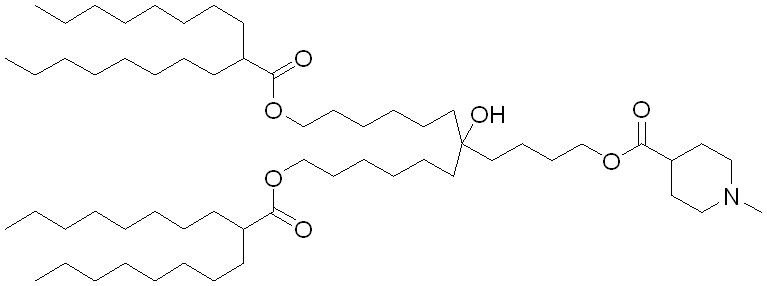


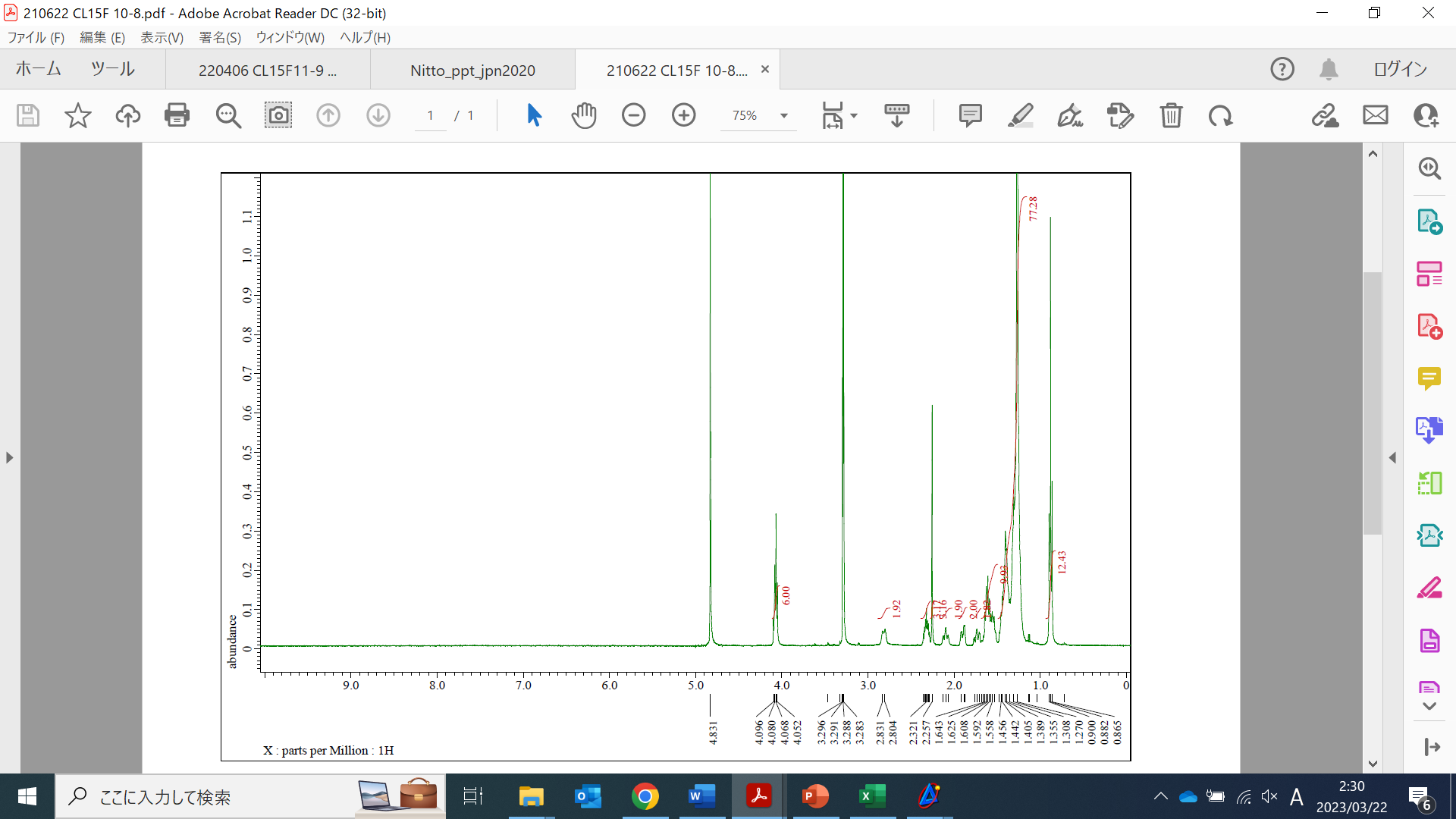


CL15F 11-7


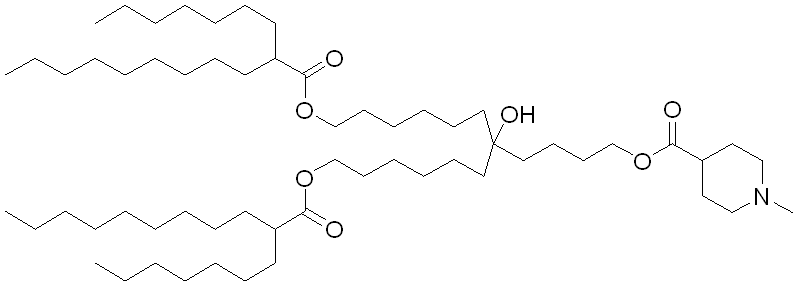


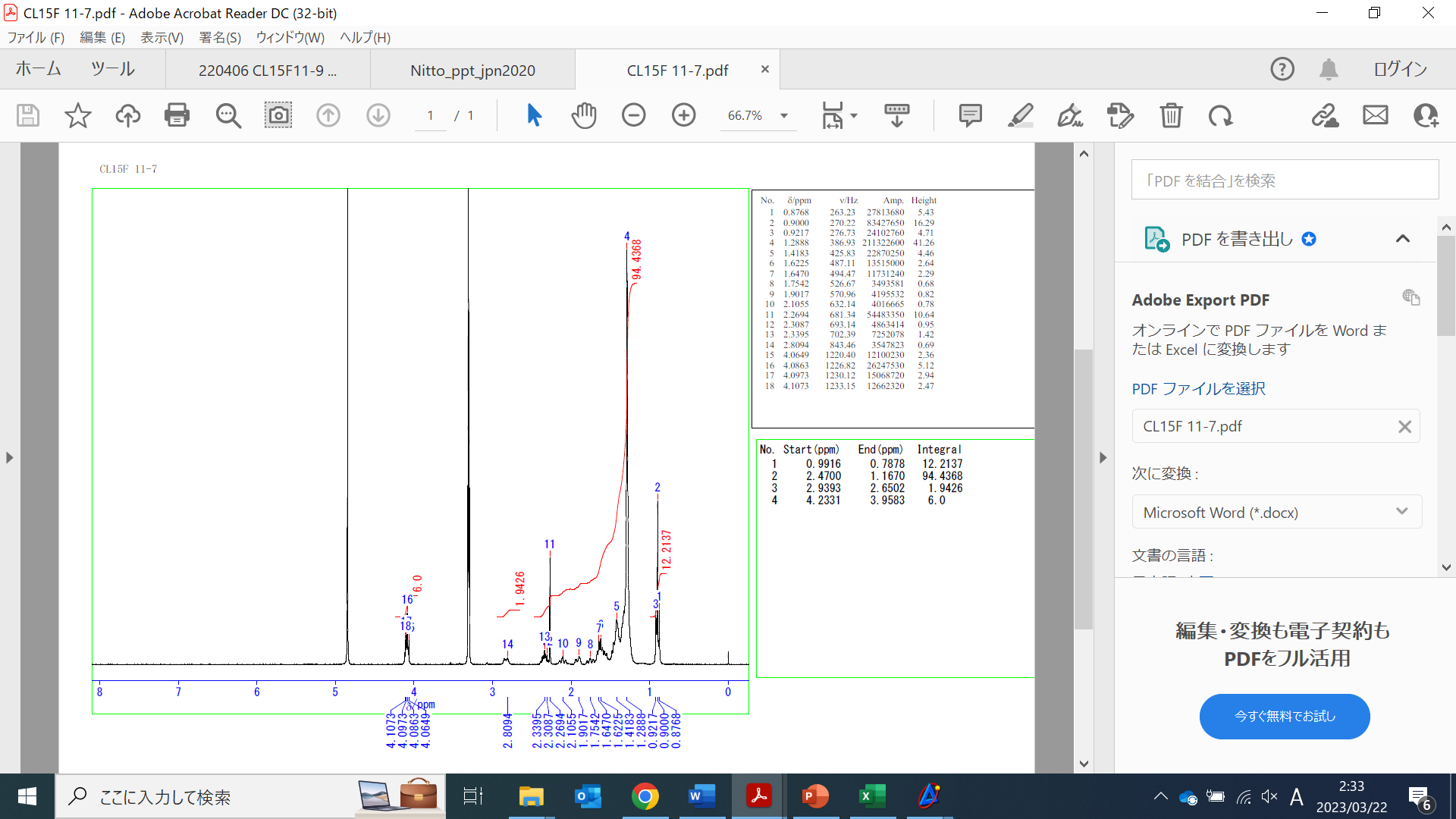


CL15F 11-9


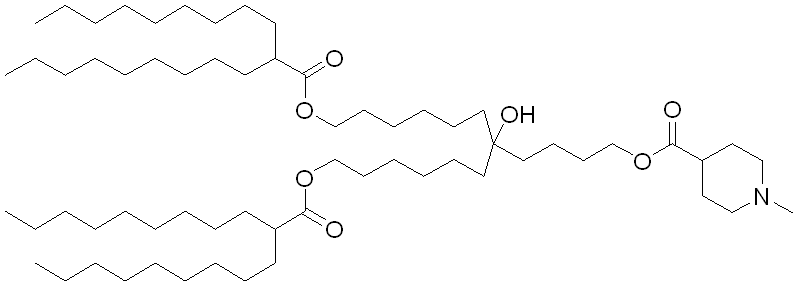


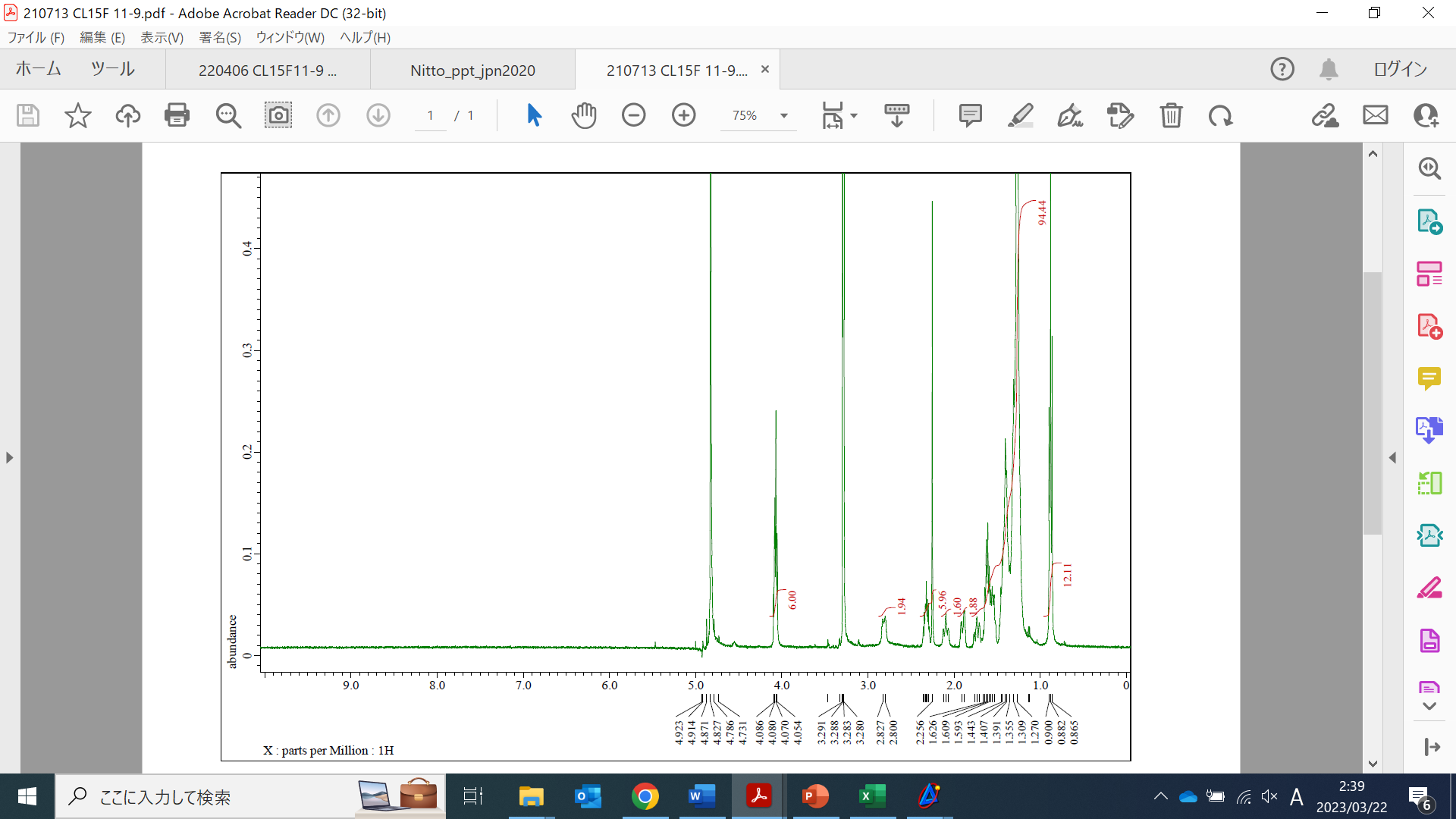


CL15F 12-10


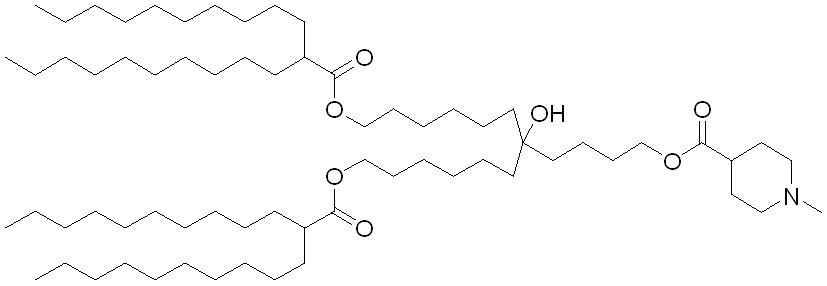


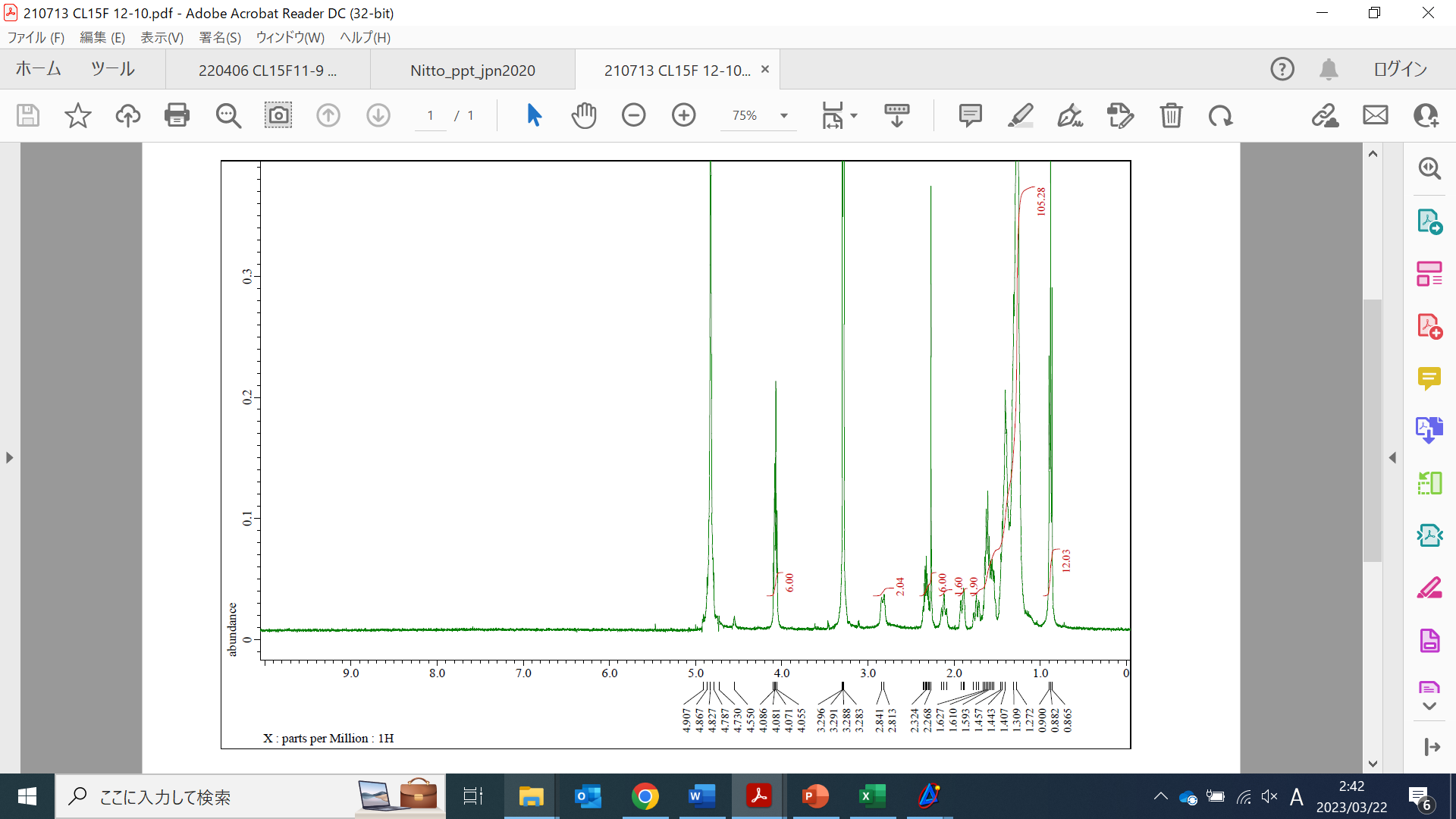

CL15F 14-2


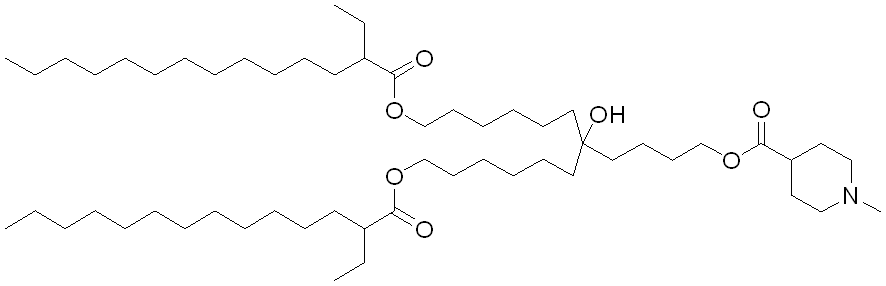


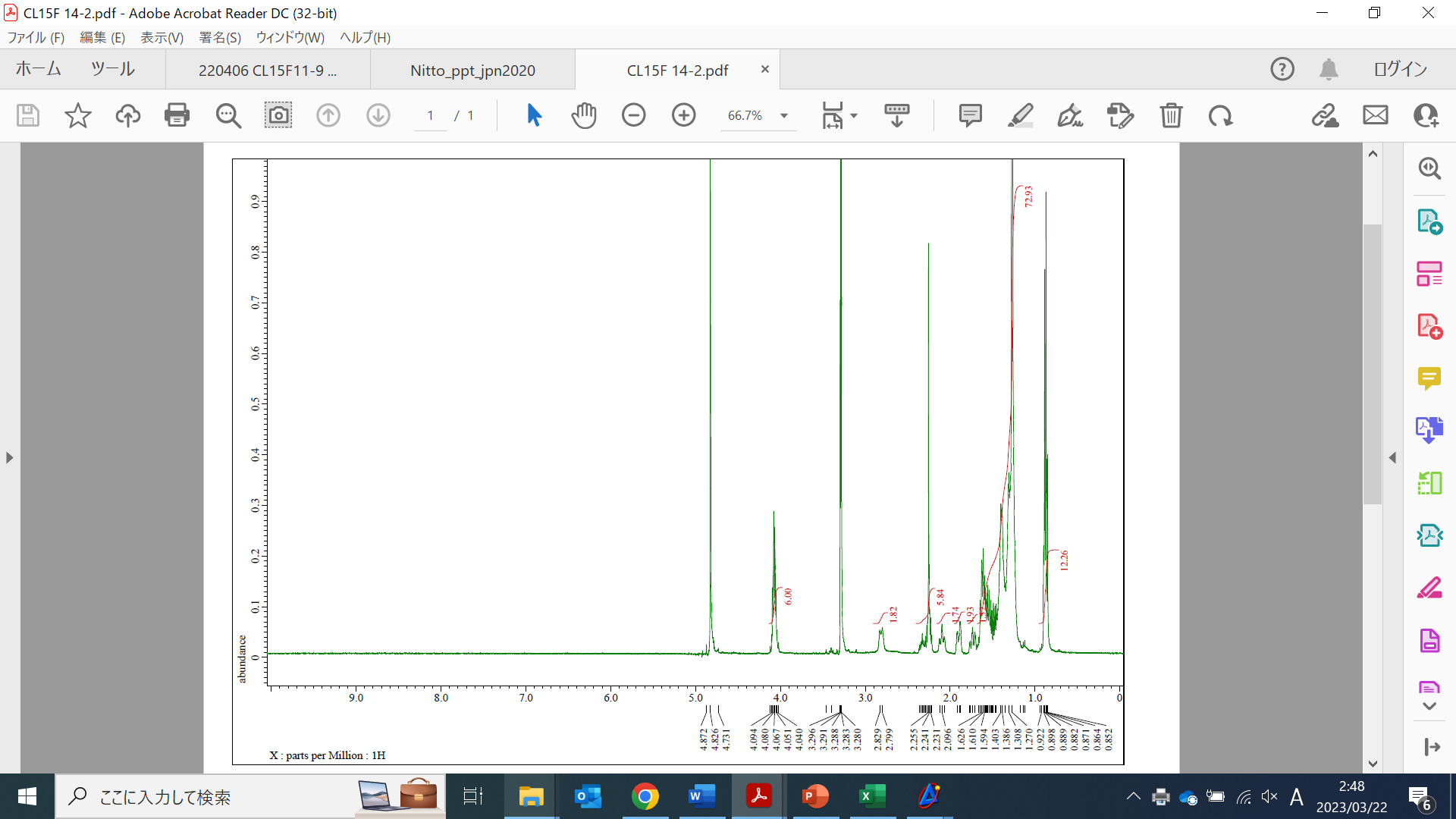


CL15F 10-6


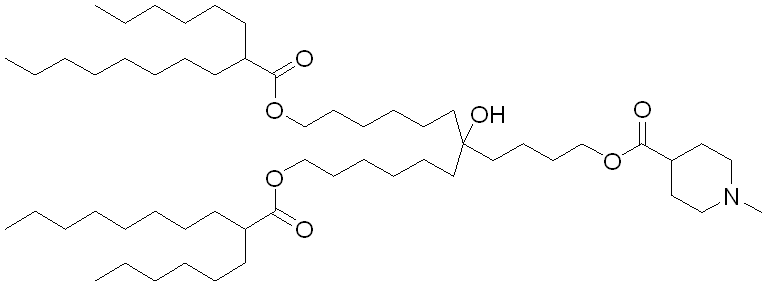


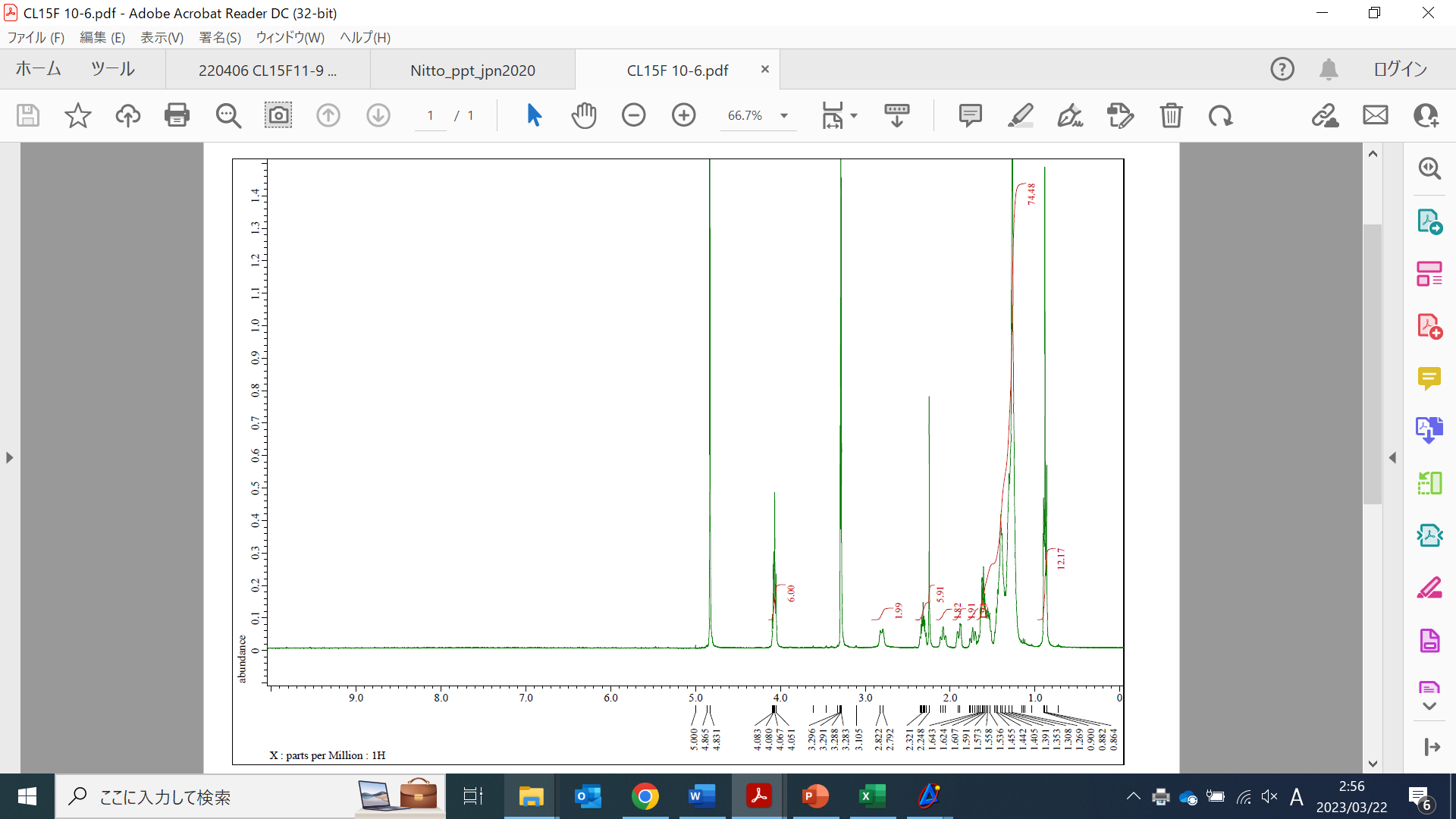


CL15F 14-12


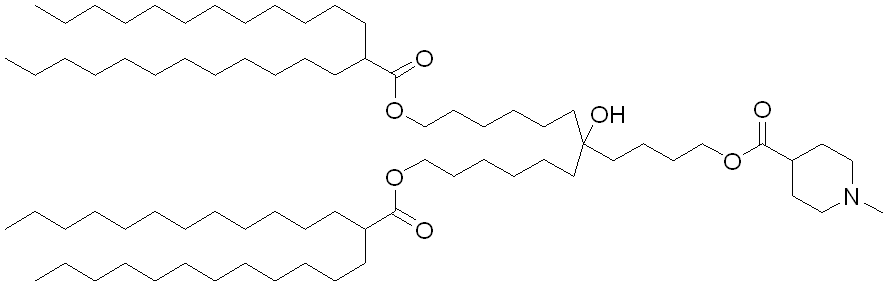


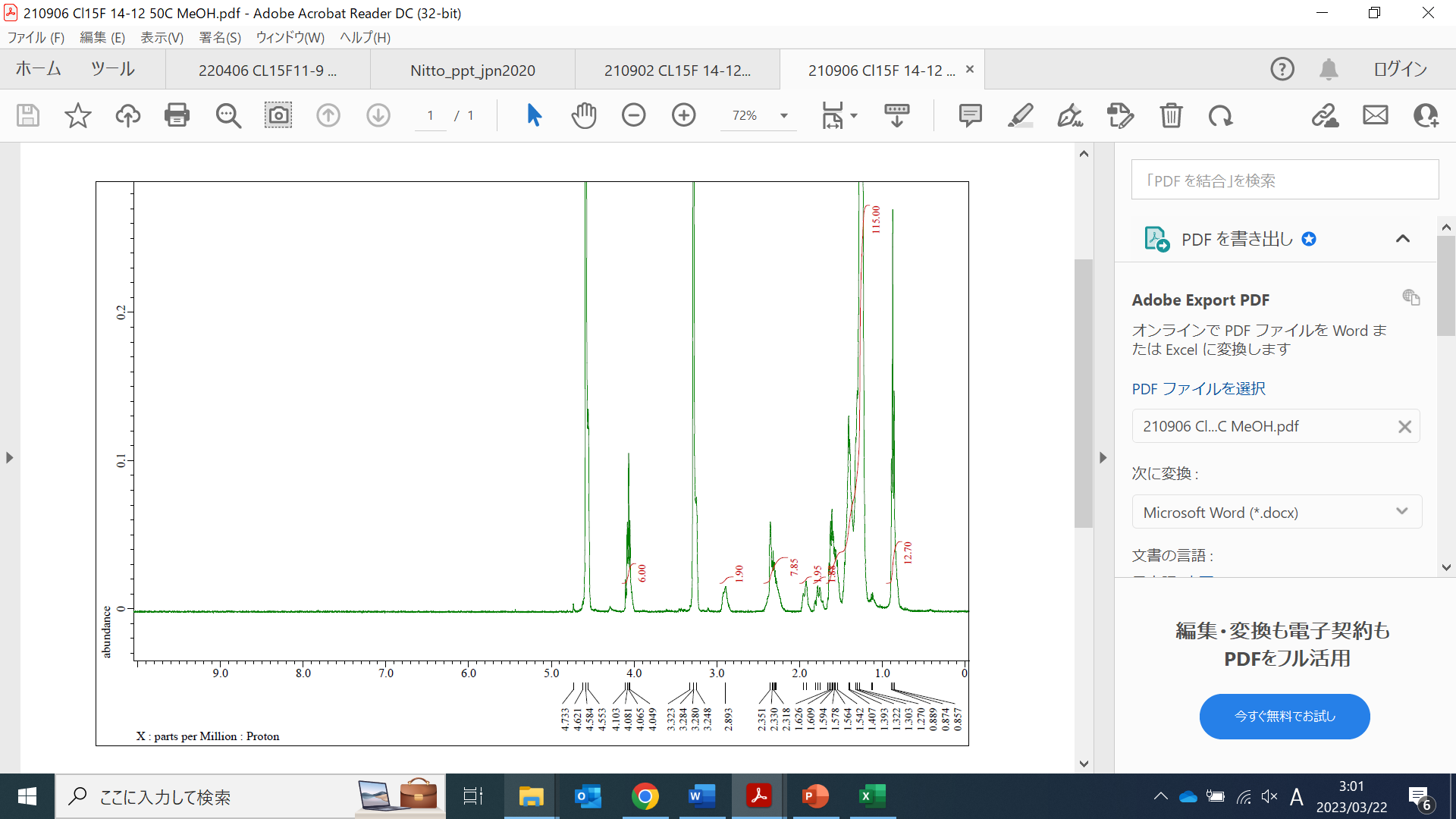

CL6F 14-12


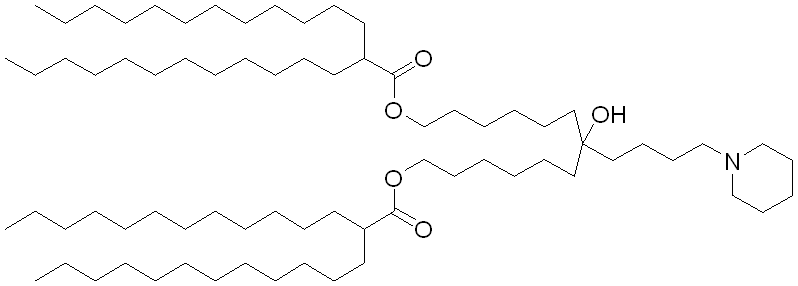


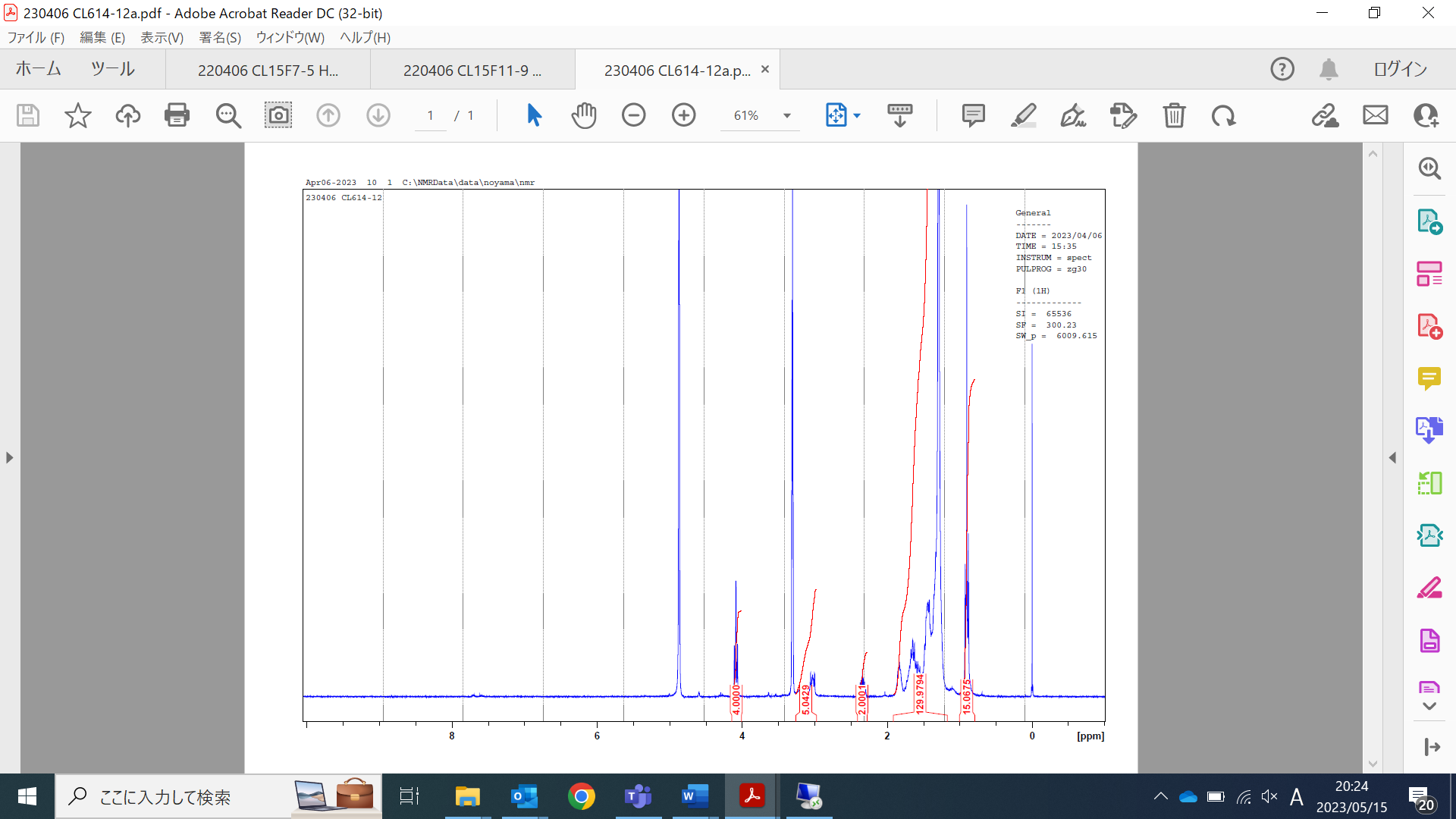


CL16F 14-12


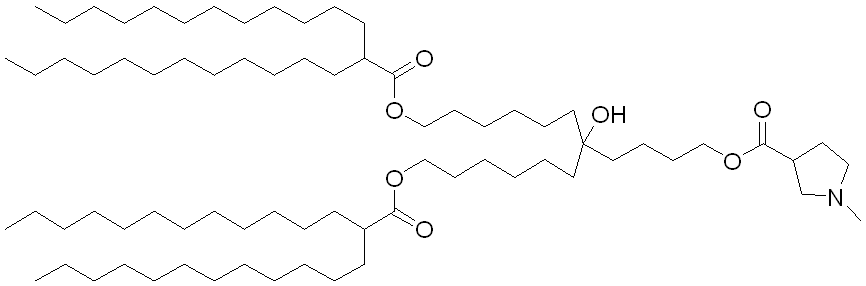


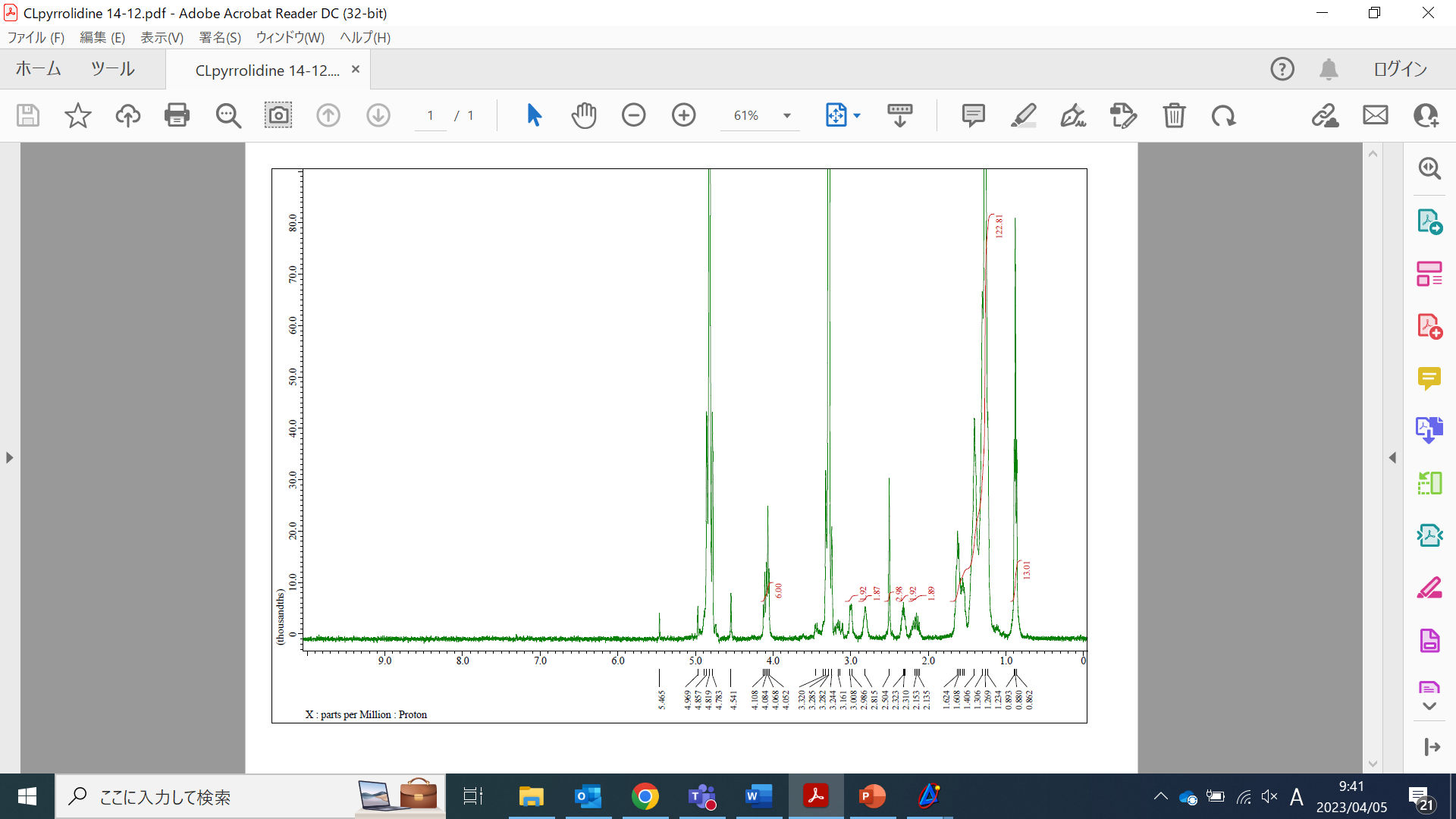


CL17F 14-12


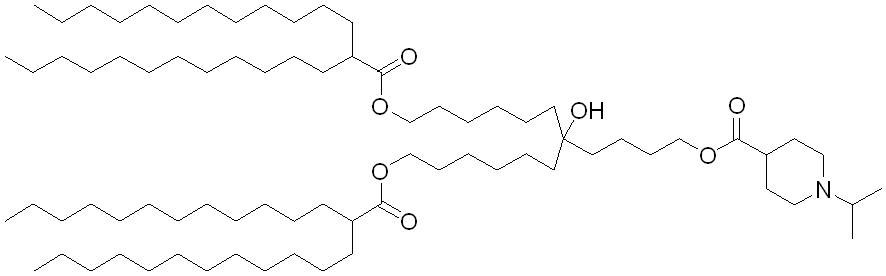


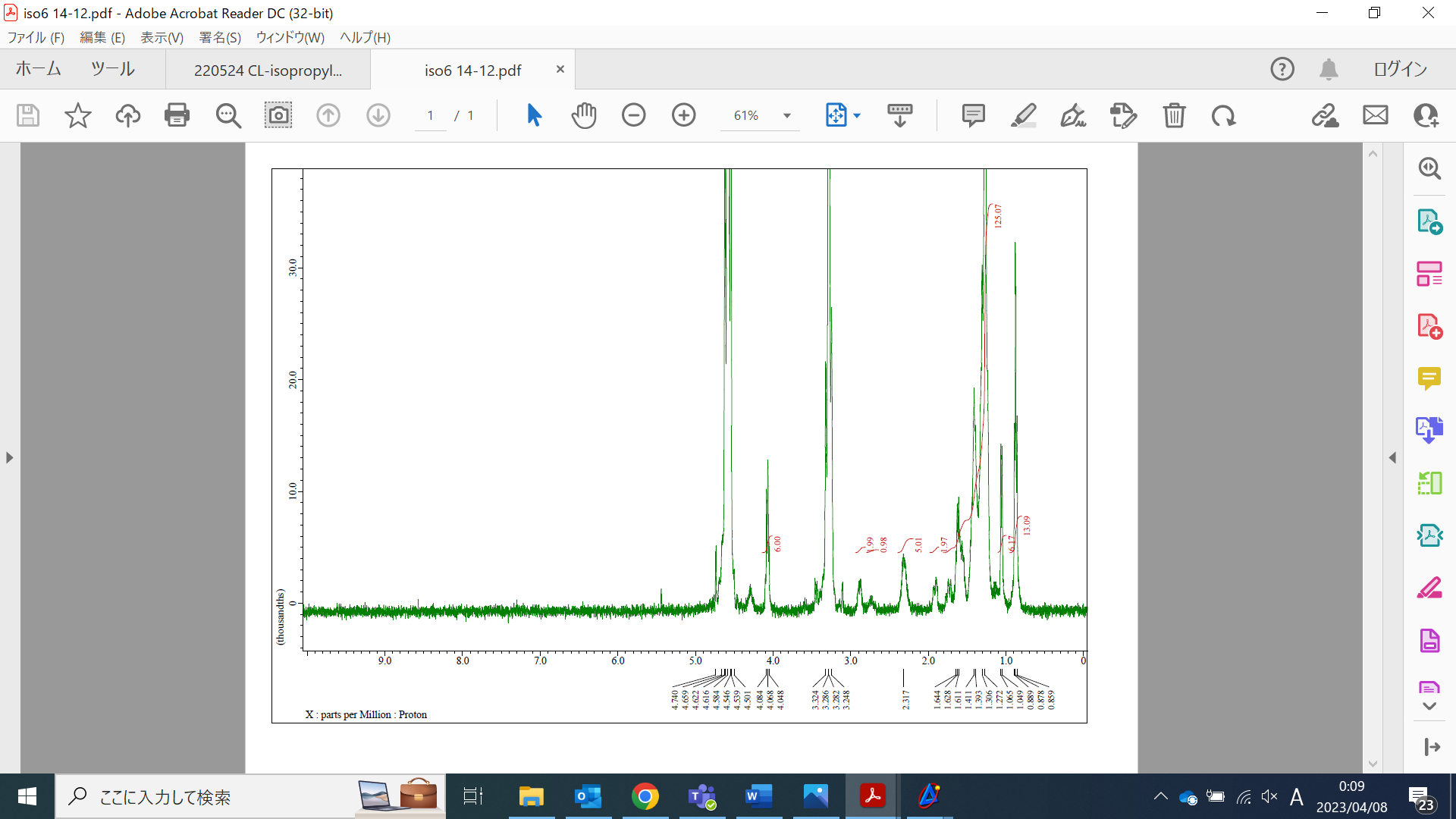


# **References**

1. Imai, K. & Watanabe, Y. Fluorimetric determination of secondary amino acids by 7-fluoro-4-nitrobenzo-2-oxa-1,3-diazole. *Anal. Chim. Acta* **130**, 377–383 (1981).

2. Hashiba, K. *et al.* Branching Ionizable Lipids Can Enhance the Stability, Fusogenicity, and Functional Delivery of mRNA. *Small Sci.* **3**, 2200071 (2023).
