# Supplementary material for: Overcoming thermostability challenges in mRNA–lipid nanoparticle systems with piperidine-based ionizable lipids
Source: Commun Biol. 2024 May 10;7:556. doi: 10.1038/s42003-024-06235-0 (PMC11087515; doi:10.1038/s42003-024-06235-0)
Supplement: Supplementary file 3 — Description of additional supplementary files [file 42003_2024_6235_MOESM3_ESM.docx]

Description of Additional Supplementary Files

**File name:** Supplementary Data 1

**Description:** All source data underlying the graphs and charts presented in the main figures.
